# Chemical imaging reveals diverse functions of tricarboxylic acid metabolites in root growth and development

**DOI:** 10.1038/s41467-023-38150-z

**Published:** 2023-05-04

**Authors:** Tao Zhang, Sarah E. Noll, Jesus T. Peng, Amman Klair, Abigail Tripka, Nathan Stutzman, Casey Cheng, Richard N. Zare, Alexandra J. Dickinson

**Affiliations:** 1 Cell and Developmental Biology, University of California San Diego, La Jolla, CA 92093 USA; 2 Department of Chemistry, Stanford University, Stanford, CA 94305 USA; 3 Department of Chemistry, Pomona College, Claremont, CA 91711 USA

**Keywords:** Plant development, Small molecules, Plant biotechnology

## Abstract

Understanding how plants grow is critical for agriculture and fundamental for illuminating principles of multicellular development. Here, we apply desorption electrospray ionization mass spectrometry imaging (DESI-MSI) to the chemical mapping of the developing maize root. This technique reveals a range of small molecule distribution patterns across the gradient of stem cell differentiation in the root. To understand the developmental logic of these patterns, we examine tricarboxylic acid (TCA) cycle metabolites. In both Arabidopsis and maize, we find evidence that elements of the TCA cycle are enriched in developmentally opposing regions. We find that these metabolites, particularly succinate, aconitate, citrate, and α-ketoglutarate, control root development in diverse and distinct ways. Critically, the developmental effects of certain TCA metabolites on stem cell behavior do not correlate with changes in ATP production. These results present insights into development and suggest practical means for controlling plant growth.

## Abstract (web summary)

Chemical imaging of maize roots reveals differential patterns of TCA cycle metabolites along the axis of development. Root growth is affected in distinct ways by exogenous treatments and tissue-specific genetic manipulation of TCA metabolites.

## Introduction

**Fig. 1:**
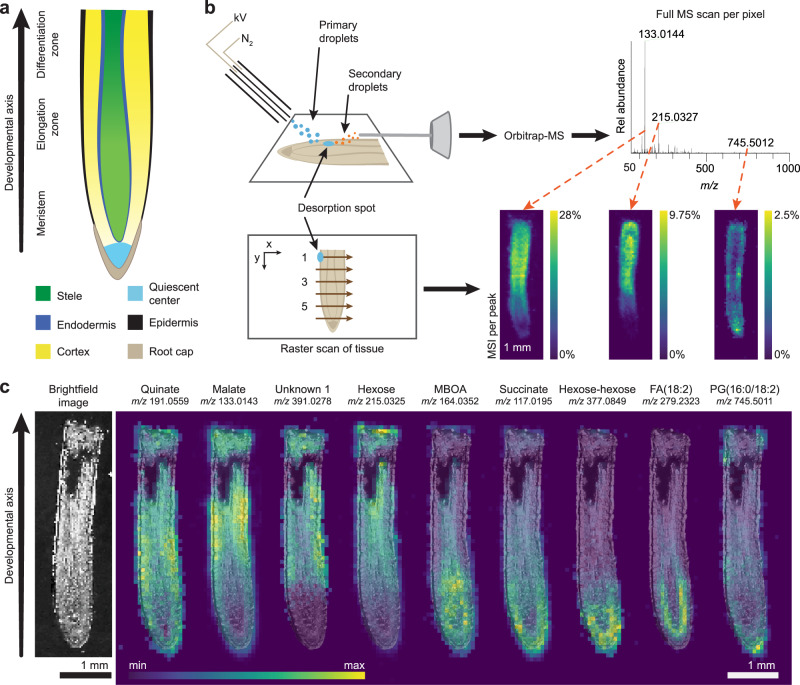
DESI-MSI captures the distinct distribution patterns of endogenous molecules within the developing maize root. **a** Maize root tip with developmental zones and tissue types. **b** Schematic of DESI-MSI shows the mechanism of ambient desorption and ionization by continuous spray of charged droplets, where one full scan is captured per pixel. The desorption spot is moved in a raster scan across the tissue to produce a pixelated map. Each peak in the full spectrum corresponds to one MS image. **c** Brightfield image of a maize root section prior to DESI imaging. DESI-MS images show that certain metabolites and lipids are most intense in distinct tissues and developmental zones. MS images are in the negative ionization mode and are overlaid with the brightfield image. The maximum intensity of each ion as a percentage of total ion current is: quinate, 1.40%; malate, 21.8%; unknown 1, 1.13%; hexose, 12.3%; 6-methoxy-2-benzoxazolinone (MBOA), 2.74%; succinate, 5.07%; hexose-hexose, 1.47%; fatty acid (18:2), 3.83%; and phosphatidylglycerol (16:0/18:2), 2.61%. This experiment was replicated ten times across three biological samples (Supplementary Table 1). Data from one representative tissue section are shown here. MS images of all ten sections are available in Supplementary Figs. 11–17, 23, 25.

In the study of multicellular development, plant roots present a unique opportunity. The longitudinal axis of a root transitions through multiple stages of development, from the birth of new stem cells in the rapidly dividing meristem to formation of mature cells in the differentiation zone. This permits the observation of continuous developmental gradients within a single slice of tissue (Fig. 1a). Elucidation of mechanisms that regulate root growth have relied extensively on visualization of transcriptional and translational reporters along this developmental gradient. Although small molecules are essential regulators of root development, it has remained a challenge to visualize them in their native context^1^.

Mass spectrometry imaging, a technique capable of addressing this challenge, is emerging as a powerful tool in the study of plant biology. Desorption electrospray ionization mass spectrometry imaging (DESI-MSI)^2^ has been previously employed to image various plant tissues via indirect imprinting^3–10^, direct surface measurement^3,5,10–14^, and, less commonly, thin cryosections^13,14^. In DESI-MSI of biological samples, a stream of charged droplets of a selected solvent mixture is directed at the surface of a tissue section under ambient conditions, forming a thin film known as the desorption spot (Fig. 1b)^2^. Continued spraying carries sample material dissolved into this spot to the mass spectrometer for subsequent detection. By moving the desorption spot in a raster scan across the tissue section, a pixelated image is formed, where each pixel (picture element) contains a full mass spectrum.

Here, we apply DESI-MSI to map the relative intensities of molecules along the developmental gradient of the maize root. Horizontal cross-sections of maize root have been imaged by matrix-assisted laser desorption/ionization mass spectrometry imaging (MALDI-MSI)^15–17^, which can achieve finer spatial resolution using laser desorption than DESI-MSI can with liquid desorption. While these maize studies provide excellent spatial detail in the cross-sections, they do not capture the developmental gradients within a single root section, as we attempt here and has been previously shown in barley root by MALDI-MSI^18^. Compared to MALDI-MSI, DESI-MSI has the advantage of requiring no matrix application or derivatization to capture a wide class of molecules, although derivatization can be beneficial for targeting specific analytes^11^.

Our approach to chemically image the developmental axis of the maize root using DESI-MSI reveals a range of molecules with differential enrichment patterns. In particular, we applied this technique to map lipids, carbohydrates, and metabolites across the developing root. We then explored the chemical logic of these patterns in TCA metabolites, a subset of primary metabolites with distinctive enrichment patterns in the root.

## Results

### DESI-MSI reveals small molecule distribution patterns along the developmental axis of the maize root

We imaged thin cryosections of maize (*Zea mays*) root tissue after careful adaptation of the DESI imaging technique to resolve the fine features within each root. Specifically, we modified the DESI sprayer and imaging parameters, which doubled the resolution of our method compared to what is commonly found in DESI-MSI^19^ (see Methods and Supplementary Figs. 1–3). We coupled this technique to a high-resolution accurate mass (HRAM) orbitrap mass spectrometer for semi-quantitative spatiochemical characterization.

**Fig. 2:**
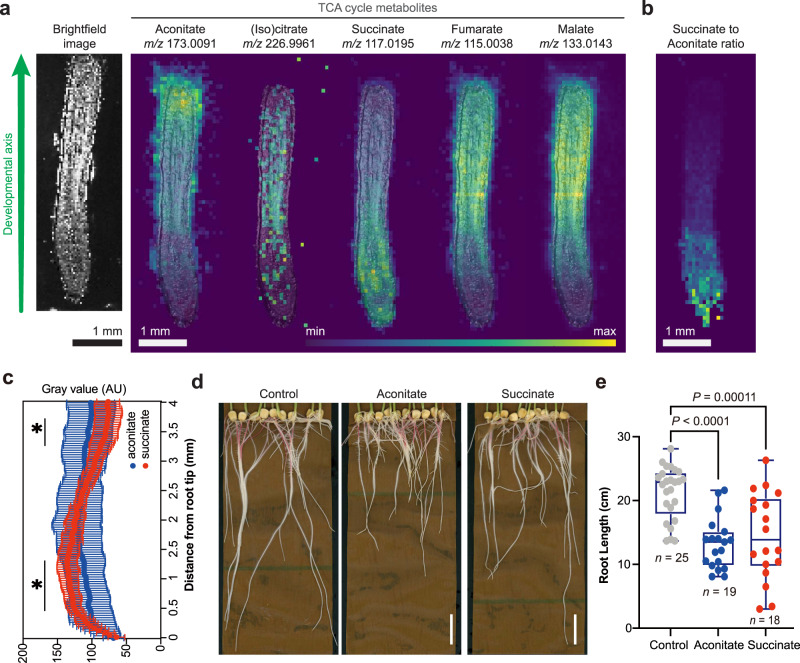
The TCA cycle metabolites are differentially distributed along the developmental axis of the maize root. **a** DESI-MS images of the detectable TCA cycle metabolites overlaid with a brightfield image captured prior to MS imaging. The maximum ion intensity as % TIC is different for each ion and is: aconitate, 2.99%; citrate/isocitrate, 0.271%; succinate, 3.12%; fumarate, 4.55%; and malate, 28.0%. **b** MS image of the intensity ratio of succinate to aconitate per pixel. Maximum of ratio scale is 11 (yellow). Imaging experiments were replicated ten times across three biological samples (Supplementary Table 1). Data from one representative tissue section are shown here. MS images of all ten sections are available in Supplementary Figs. 21–30. **c** Normalized intensity of aconitate and succinate along the root axis. The data are presented as means ± s.d. (*n* = 10 root sections across three biological replicates, Supplementary Table 1). Asterisks indicate statistical significance by two-tailed unpaired Student’s *t*-test (*P < 0.05, detailed *P*-values for each point can be seen in the Source Data file). All MS data were acquired in the negative ionization mode. **d**, **e** Maize root phenotypes under 10 mM aconitate and succinate treatment, respectively. Scale bars, 3 cm. For the boxplots, each dot represents the datapoint of one biological replicate; the central line indicates the median; the bounds of the box show the 25th and 75th percentiles; and the whiskers indicate maximum and minimum values. All *P*-values were determined by one-way ANOVA (all *n* and *P*-values are indicated in the graphs). Source data are provided as a Source Data file.

MS images revealed a wealth of lipids, carbohydrates, and primary and secondary metabolites, including benzoxazinoids, a class of plant defense molecules (Fig. 1c, Supplementary Figs. 11–17, 23, 25)^20–22^. Between 20 to 30 small molecules were localized per developmental zone (Supplementary Figs. 18–20). We hypothesized that these enrichment patterns might predict developmental functions. Particularly striking were the observed differential gradients in the normalized intensity of TCA cycle metabolites along the developmental axis (Fig. 2a–c, Supplementary Figs. 21–29, odd-numbered). In particular, succinate was most intense in the meristem. In contrast, aconitate, malate, and fumarate signals were typically enriched toward the region of root differentiation. We also observed an *m/z* signature consistent with both citrate and isocitrate, which are isomers and cannot be distinguished by mass spectrometry alone (Fig. 2a, Supplementary Table 2). To understand the developmental reasoning behind differential small molecule distribution patterns, we focused subsequent studies on determining whether individual TCA metabolites have specific effects on stem cell behavior.

### TCA metabolites cause diverse developmental phenotypes in maize and Arabidopsis

We applied exogenous TCA metabolites to maize to explore the roles of these compounds in maize root development and growth. We found 10 mM aconitate, succinate, and malate inhibited primary root length (Fig. 2d, e, Supplementary Fig. 4e, f) compared to control plants. Broadly, except for aconitate treatment, which increased citrate/isocitrate levels, none of these treatments led to significant changes in the overall profile of TCA metabolites compared to control plants (Supplementary Fig. 4h), suggesting that the pathway is not highly sensitive to influx of these metabolites.

**Fig. 3:**
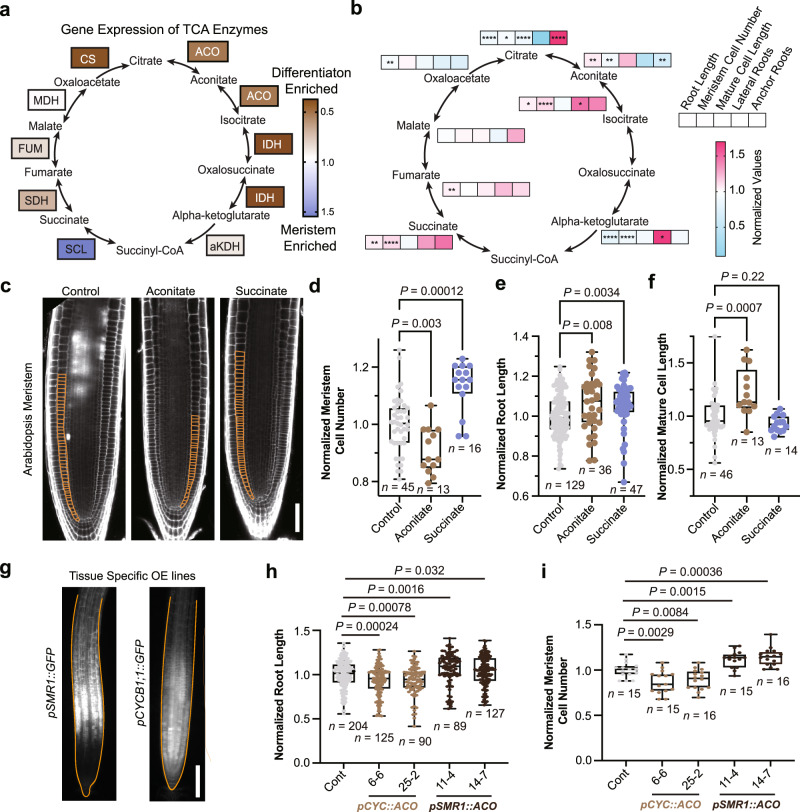
Succinate and aconitate have opposing effects on meristem cell divisions in Arabidopsis. **a** Enrichment of the expression levels (log[3xFPKM]) of TCA biosynthesis genes in the root meristem. Data were quantified from Li S. et al.^24^. Enrichment was calculated by dividing the average expression in the meristem by the average expression in the differentiation zone. **b** Heat map of Arabidopsis root phenotypes under 1 mM TCA metabolite treatments. **c** Confocal images of root meristems treated with 1 mM succinate or aconitate, respectively. Scale bar, 100 μm. Meristematic cortex cells are outlined in orange. **d**–**f** Quantification of the effects of 1 mM treatments on the number of meristematic cells (**d**), the total primary root length (**e**), and the average cell length of the mature root (**f**), normalized to the control. **g** Transcriptional reporters for *SMR*, a promoter specific to the early differentiation zone, and *CYCB1;1*, a meristem promoter. Scale bar, 150 μm. Root outlines are highlighted in orange. **h**, **i** Quantification of primary root length and number of meristem cells in genetically engineered lines, respectively. (*CYC* = *CYCB1;1*, *ACO* = *ACONITASE 1*). Data were normalized to the control treatment for each genotype. For the boxplots, each dot represents the datapoint of one biological replicate; the central line indicates the median; the bounds of the box show the 25th and 75th percentiles; and the whiskers indicate maximum and minimum values. All *P*-values were determined by one-way ANOVA in (**d**–**f**) and by two-tailed unpaired Student’s *t*-tests in (**b**, **h**, **i**). All *P*-values are shown in the plots, except for (**b**) which are provided in the Source Data (*P < 0.05, **P < 0.01, ***P < 0.001, ****P < 0.0001). Source data are provided as a Source Data file.

To delve more deeply into the developmental functions of TCA metabolites in the root, we characterized their effects on the tractable model plant, Arabidopsis (*Arabidopsis thaliana*). Previous work has shown that TCA cycle intermediates have different effects on growth in the dark-grown Arabidopsis roots and in the hypocotyl, suggesting that there are tissue-specific responses to the TCA cycle metabolites in this plant^23^. However, Arabidopsis roots, typically ~100 μm in width, are too small to image with our current DESI-MSI capabilities; so, to explore whether TCA metabolites have developmental distribution patterns in Arabidopsis, we used a previously published RNA-Seq data set^24^ to calculate the expression levels of TCA biosynthesis genes in the meristem and maturation zones (Fig. 3a). We found spatially distinctive distribution patterns of several classes of TCA biosynthesis genes (Fig. 3a). In particular, *ACONITASE* (*ACO*), *SUCCINATE-COENZYME A LIGASE* (*SCL*), *SUCCINATE DEHYDROGENASE* (*SDH*), and *FUMARASE* (*FUM*) gene expression levels in Arabidopsis correlated with the metabolite distributions observed in maize roots.

Because most of the TCA biosynthesis genes are knockout-lethal, we applied exogenous TCA metabolite treatments to determine if these compounds induce changes in development. We chose to test 1 mM and 5 mM concentrations of each metabolite, based on exogenous treatment concentrations typically used in mammalian cell research (1–50 mM)^25,26^ and published endogenous concentration levels of TCA metabolites in plants (1–50 mM)^27–31^. We identified distinct and concentration-dependent effects of several TCA metabolites on root development (Fig. 3 and Supplementary Fig. 5). Treatment with each TCA metabolite generates different changes in root growth, formation of branching roots (lateral roots and anchor roots), and root hair development, which is a hallmark feature of tissue differentiation. For example, treatment with either α-ketoglutarate or citrate (buffered to a pH of 5.7) inhibits primary root growth (Supplementary Fig. 5). However, these treatments have opposite effects on root branching. Citrate decreases lateral root number, but increases anchor root growth, whereas α-ketoglutarate significantly increases lateral root number and has no effect on anchor roots. Overall, investigation of the full TCA metabolite pathway reveals numerous distinct functions in root development and growth. Due to the complexity of the phenotypes induced by TCA metabolites, we chose to focus on well-characterized root stem cell behaviors: (1) divisions in the root meristem, (2) cell elongation upon exiting the meristem, and (3) differentiation into root hairs.

### TCA metabolite treatments alter meristematic cell divisions in Arabidopsis roots

To determine how TCA metabolites affect root growth, we measured meristem activity using confocal microscopy of exogenously treated roots (Fig. 3b-c, Supplementary Fig. 5). Citrate, α-ketoglutarate, and aconitate significantly reduce meristem cell numbers. Isocitrate and succinate have the opposite effect, increasing meristem cell numbers significantly. Aconitate was the only metabolite that increases cell elongation, resulting in longer differentiated cells (Fig. 3d–f, Supplementary Fig. 5). Together, these results demonstrate that TCA metabolites lead to distinct root phenotypes in stem cell divisions and growth. Furthermore, these phenotypes typically correlate with their localization in the root. For instance, meristem-localized succinate is a meristem growth promoter whereas aconitate, which is typically enriched in the differentiation zone, inhibits meristem growth and promotes cell elongation. These correlations indicate that meristem enrichment can be predictive of molecular functions in stem cell behavior.

To explore the roles of TCA metabolite distribution patterns with more spatial precision, we investigated the effect of changing them genetically. We ectopically overexpressed TCA metabolite biosynthesis genes in specific developmental zones. We chose the meristem promoter *pCYCB1;1*^32^ and the differentiation zone promoter *pSMR1*^33^ to drive tissue-specific expression of TCA-related genes (Fig. 3g–i, Supplementary Fig. 6). We found that disrupting the normal expression patterns of aconitate biosynthesis induced significant changes in root growth and development. Overexpressing *ACO1* in the meristem using the *CYCB1;1* promoter significantly decreases primary root length, which was due to fewer meristem cells and shorter mature cells compared to controls (Fig. 3h–i, Supplementary Fig. 6). In contrast, overexpressing *ACO1* in the differentiation zone significantly increases root growth compared to controls, due to enhances in meristematic cell number (Fig. 3h–i, Supplementary Fig. 6). This finding is consistent with exogenous treatment results that show that aconitate promotes elongation in differentiating cells and inhibits cell divisions in the meristem (Fig. 3d–f). To explore TCA distribution patterns in sensitized conditions, we treated the transgenic plants with different stresses. We found that changing the location of *ACO* overexpression from the meristem to the differentiation zone resulted in a different response to phosphate deprivation stress (-P), although not to osmotic stress (Supplementary Fig. 7). Overall, *pCYC::ACO* lines are more resistant to -P stress conditions compared to *pSMR1::ACO*. These results indicate that changing the distribution pattern of a single aconitate biosynthesis gene is sufficient to alter root development and stress response.

### ATP levels are not affected by TCA metabolite treatment in Arabidopsis

One question these experiments raised is whether the phenotypes we observe result from a change in cellular energy, as the TCA cycle is a major source of ATP production. To determine whether these treatments affect ATP balance in the roots, we analyzed the effects of TCA metabolites on a fluorescent ATP sensor^34^. We found that the localization pattern and intensity of the ATP sensor was fairly stable in response to changing TCA cycle inputs (Supplementary Fig. 8). Significant changes were induced by oxaloacetate and α-ketoglutarate treatments, which both increased the intensity of the ATP sensor in the meristem. Isocitrate was the only metabolite tested that significantly increased the intensity of the ATP sensor in the meristem and differentiation zone. This is consistent with the positive effect that isocitrate has on cell divisions (Supplementary Fig. 5d). These results suggest that isocitrate may provide a useful new starting point for biotechnology focused on increasing root ATP production and growth. Interestingly, these results demonstrated that changes in ATP levels are not sufficient to fully understand the differential phenotypes caused by individual TCA metabolite treatments.

### TCA metabolites had differential effects on the balance of reactive oxygen species (ROS) in Arabidopsis roots

Previous work on Arabidopsis roots has demonstrated that the spatial balance between hydrogen peroxide (H_2_O_2_) and superoxide (O_2_^•−^) is important for regulating the transition between stem cell proliferation and differentiation in the root^35–38^. The alterations to cellular redox states caused by increases in H_2_O_2_ and O_2_^•−^ levels have been linked to the inhibition and maintenance of meristem cell divisions, respectively. SDH is a mitochondrial source of ROS in plants and animals^39,40^. To test whether TCA metabolite treatments, particularly succinate treatment, regulate growth through ROS, we measured H_2_O_2_ and O_2_^•−^ in the root (Supplementary Fig. 9). We found that all eight of the TCA metabolites tested increase O_2_^•−^ in the lower meristem, upper meristem, and/or differentiation zone (Supplementary Fig. 9a, b). Succinate increases O_2_^•−^ in the lower meristem and differentiation zone, which is consistent with the role of O_2_^•−^ in promoting meristem divisions. However, given the general induction of O_2_^•−^ by the TCA metabolites, this result is not sufficient to explain the specific promotional effect of succinate in cell divisions. Succinate also significantly increases H_2_O_2_ levels in the lower meristem (Supplementary Fig. 9c, d), which would be expected to inhibit growth. This result further indicates that succinate’s effect on ROS is not sufficient to fully understand its role in root growth. The only other TCA metabolite that alters the levels of H_2_O_2_ in the plant root at 1 mM concentration is α-ketoglutarate, which dramatically reduces H_2_O_2_ in the meristem (Supplementary Fig. 9c, d). Notably, α-ketoglutarate treatment alters the balance of H_2_O_2_ and O_2_^•−^ in a way that would be predicted to stimulate cell divisions, but instead it causes the opposite effect. Overall, ROS levels were highly sensitive to the TCA metabolite treatments, but the changes we measured did not fully explain the different effects of treatments on the root meristem. A hypothesis consistent with these results and previous literature^36,37^ is that the meristematic response to TCA metabolites is occurring through a different signaling pathway that overrides ROS regulation of meristem activity.

### TCA metabolites regulate root hair growth in maize and Arabidopsis via H_2_O_2_

We observed consistent effects of the TCA metabolites on root hair development in maize and Arabidopsis. Root hairs are essential for collecting water, absorbing nutrients, promoting beneficial microorganismal interactions, and increasing carbon sequestration. Previous studies have shown that root hair development is tightly regulated by H_2_O_2_^35^. Specifically, increases in H_2_O_2_ levels result in longer, more dense root hairs, whereas decreasing H_2_O_2_ has the opposite effect.

**Fig. 4:**
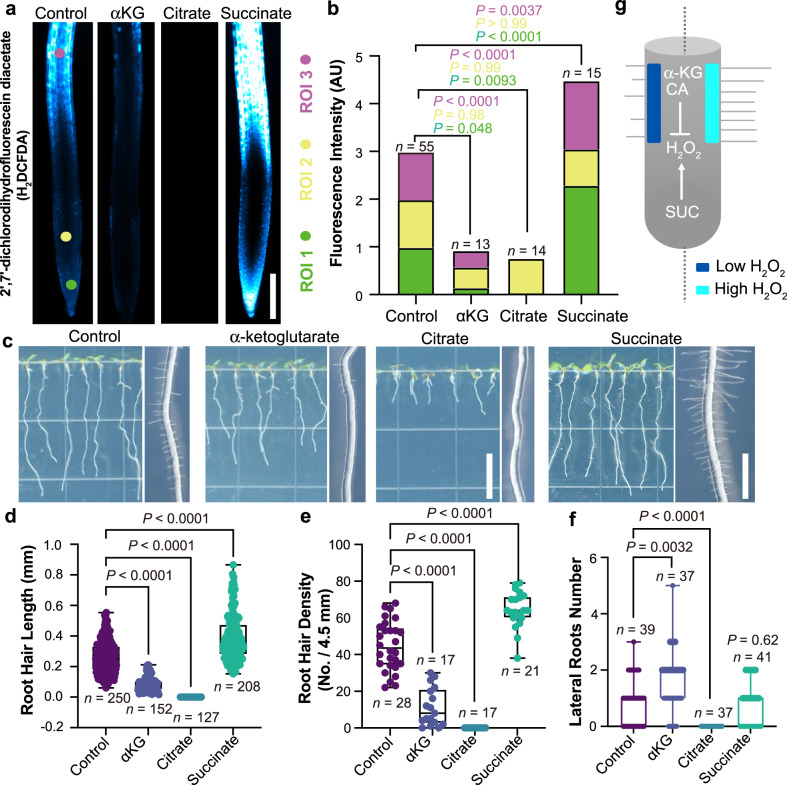
TCA metabolites alter ROS levels and root hair development in Arabidopsis. **a** Images of H_2_DCFDA, a biological reporter for H_2_O_2_, in roots treated with 5 mM TCA metabolites. Scale bar, 200 μm. **b** Quantification of the H_2_DCFDA fluorescence intensity in three regions of interest (ROIs). Data represent means. All *P-*values were determined by one-way ANOVA. **c** Images of Arabidopsis roots treated with 5 mM TCA metabolites. Scale bar for primary root (left), 1 cm. Scale bar for root hair (right), 1 mm. **d**, **e** Quantification of the effects of 5 mM TCA metabolite treatments on root hair phenotypes. All P-values were determined by one-way ANOVA. For the boxplots, the central line indicates the median; the bounds of the box show the 25th and 75th percentiles; and the whiskers indicate maximum and minimum values. **f** Effect of TCA metabolites on lateral root number. All *P*-values were determined by two-tailed unpaired Student’s *t*-test. **g** A model for how succinate (SUC), citrate (CA), and α-ketoglutarate (αKG) influence root hair development through their effects on H_2_O_2_ levels. Source data are provided as a Source Data file.

In Arabidopsis, we found that 5 mM succinate treatment dramatically increases H_2_O_2_ levels throughout the root (Fig. 4a, b). In addition, succinate also increases root hair length and density compared to control seedlings (Fig. 4c–e), which is the expected phenotype based on its effect on H_2_O_2_. In roots treated with 5 mM α-ketoglutarate and 5 mM citrate, respectively, the difference between lateral root number is greatly enhanced compared to 1 mM treatment (Fig. 4c, f, Supplementary Fig. 5). However, both treatments significantly reduce H_2_O_2_ and lead to smooth roots with shorter and less dense root hairs (Fig. 4b–e). We also observed the same effect in maize under 10 mM TCA treatments (Supplementary Fig. 10). These results are also consistent with a model wherein TCA metabolites alter root hair development by changing H_2_O_2_ levels (Fig. 4g). Overall, these results suggest that optimizing TCA metabolite levels and distribution patterns is a potential new strategy for improving agricultural sustainability.

## Discussion

TCA metabolites are increasingly being studied for their roles in signaling in a range of different organisms^41–44^. However, analyzing TCA metabolite signaling has been highly challenging because most biosynthetic genes are essential and cannot be inhibited or knocked out without causing severe pleiotropic effects or lethality^45–47^. In this study, spatial imaging of maize root chemistry using DESI-MSI revealed different patterns of TCA metabolite distributions along the axis of development. We found that exogenous treatments and tissue-specific genetic manipulation of TCA metabolites affected root growth in distinct ways. Certain phenotypes were readily predictable given the DESI-MSI localization data. For example, succinate and aconitate, which are enriched in the meristem and differentiation zone, respectively, have opposing effects on meristematic cell divisions. Additionally, tissue-specific overexpression of an aconitate biosynthetic gene results in changes in plant growth in control and stress conditions. We also identified phenotypes that we would not have predicted based on the DESI-MSI results—including citrate’s promotion of anchor root growth, α-ketoglutarate’s promotion of lateral root number, and a range of effects from multiple metabolites on root hair development. We found that TCA-induced phenotypes were largely uncorrelated with changes in ATP localization or production. The exception was isocitrate treatment, which increased ATP levels and stimulated root growth. ROS levels, in contrast, are highly susceptible to TCA-cycle perturbations. The effects of TCA-cycle metabolites on ROS levels are consistent with the well-characterized regulatory roles of ROS in root hair development. Overall, this work suggests that multiple TCA metabolites have noncanonical roles in root development, providing interesting opportunities to improve our knowledge of how plant stem cells divide, differentiate, and perform organogenesis.

These results suggest a promising avenue for agricultural research, where a major challenge is applying the information that we learn in model species to genetically divergent crops. Control of TCA metabolites may be particularly amenable for engineering desirable phenotypes in diverse crops because the TCA cycle is highly conserved across different kingdoms of life. Furthermore, improving strategies for crop engineering are especially important given the urgent need to improve plant growth in the increasingly harsh environmental conditions caused by climate change.

## Methods

### Mass spectrometry materials

Dimethyl-formamide (certified ACS grade), acetonitrile (optima), methanol (HPLC grade) and water (HPLC grade) were purchased from Fisher Scientific (Waltham, MA). Fused silica capillaries were obtained from Polymicro Technologies, a subsidiary of Molex (Phoenix, AZ). 1/16'' stainless steel T-junction and reducing unions was purchased from Swagelok (Solon, OH), while PEEK union assemblies, NanoTight sleeves, PFA tubing, and 1/16'' stainless steel tubing were acquired from IDEX Health & Sciences (Oak Harbor, WA). Graphite and graphite/Vespel ferrules were sourced from Restek (Bellefonte, PA). A 500 μL syringe with a 22 gauge, point type 3 (blunt) removable needle was purchased from Hamilton (Reno, NV). Fisherbrand^TM^ Superfrost Plus^TM^ slides, Fisherbrand^TM^ 5 mL sterile, single-use syringes, and 0.2 μm PES Thermo Scientific^TM^ Nalgene^TM^ Syringe Filters were purchased from Thermo Fisher Scientific (Waltham, MA).

### Plant growth conditions

For the Arabidopsis treatments, compounds were added to sterile 1/2X Murashige and Skoog (MS) plant growth media, and the pH was adjusted to 5.7 using potassium hydroxide. Seeds were sterilized using 20% bleach and sowed on the treated plant growth media. Plants were incubated in a 16 h/8 h day/night cycle growth chamber at 22 °C for 7–14 days prior to phenotyping. For the maize treatments, compounds were added to water, and the pH was adjusted to 5.7 using potassium hydroxide. Maize seeds were sterilized using 20% bleach and grown in large size seed germination pouches (https://mega-international.com) with the treatment. Plants were incubated in a 16 h/8 h day/night cycle growth chamber at 28 °C night/21 °C day for 5 days prior to phenotyping.

### RNA seq analysis

RNA-seq data was analyzed using data published in “High resolution expression map of the Arabidopsis root reveals alternative splicing and lincRNA regulation”^24^. In this work, RNA was extracted and sequenced from three regions of the developing Arabidopsis root: the meristem, elongation zone, and differentiation zone.

### Constructs

The backbone of ectopic expression constructs is the plasmid *pHDE*, given by Prof. Yunde Zhao’s lab^48^. The promoter sequences of *pSMR1* and *pCYCB1*, which are 1962 bp and 1179 bp respectively, were obtained from genome DNA by PCR. The CDS sequence of *AtACO1* was obtained from cDNA which was transcribed from RNA. Those fragments with eGFP were assembled by Gibson assembly at the *Pme*I site. The plasmids were transformed into Arabidopsis Columbia plants by Agrobacterium-mediated floral dipping. Transgenic seedlings were selected on 1/2X MS medium containing 16.7 mg/L hygromycin. We used primers synthesized by Eton Bioscience Inc. for this study. A list of these primers can be found in Supplementary Table 3. The transgenic seeds generated for this manuscript will be made available from the Arabidopsis Biological Resource Center.

### ROS staining

H_2_O_2_ was detected in roots using the 2',7'-dichlorodihydrofluorescein diacetate (H_2_DCFDA) staining method. The roots from 7-day-old seedlings were incubated in H_2_O containing 50 µM H_2_DCFDA for 30 min under dark conditions. Then, the roots were rinsed with sterilized water three times. The fluorescent signals were observed with an Echo Revolution microscope (Echo). The excitation and emission wavelengths used for detection of the signals were 488 nm and 520 nm, respectively.

To detect superoxide, seedlings were stained for 2 min in a solution of 2.5 mM Nitroblue tetrazolium (NBT) in 50 mM phosphate buffer (pH 6.1) in the dark and then rinsed three times with distilled water^49^. Images for NBT staining were obtained using a 1× objective with a Nikon SMZ1270 Stereo microscope.

### Confocal images

For *A. thaliana*, most of the roots were stained by PI (Propidium iodide) and observed under a Zeiss LSM880 confocal microscope (PI, 561 nm; GFP, 488 nm). Some of the roots were stained by PI and observed under a Leica SP8 confocal microscope (PI, 561 nm; GFP, 488 nm), thanks to the UCSD Microscopy Core—NS047101. ATP sensor lines^34^ were observed under a Leica SP8 confocal microscope (CFP, 439 nm; VENUS, 516 nm).

### HPLC-MS/MS

For *A. thaliana*, each sample contained 10 whole roots which were collected 7 days after germination. For maize, each sample had a single root tip, 0.5 cm in length, which was collected 5 days after germination. We used sterilized deionized water as solvent. Root samples were flash frozen using liquid nitrogen and ground into a powder, which was then dissolved in water. The aqueous solution was placed in a 4 °C refrigerator overnight, after which it was centrifuged at 3000 rpm for 3 min. The supernatant was filtered using a syringe with a 0.22 μm membrane filter. The filtered solution was used for final analysis.

The parameters of the HPLC-MS/MS method are as follows: Analysis was performed on a Thermo Scientific Vanquish UHPLC system coupled with an Orbitrap-MS (Orbitrap Elite, Thermo Scientific) with a heated electrospray ionization source. Chromatographic separation was carried out on a Kinetex® C18 column (150 × 2.1 mm, 1.7 mm) with a Kinetex® C18 guard column maintained at 35 °C. The mobile phases were A (H_2_O/formic acid (FA), 1000/1, v/v) and B (acetonitrile (ACN)/FA, 1000/1, v/v) with the gradient program: 0–6 min, 1% B; 6–7 min, 1% B to 90% B; 7–9 min, 90% B; 9–9.01 min, 90% B to 1% B; 9.01–12 min, 1% B. MS parameters included sheath gas flow rate of 50 arbitrary units, auxiliary gas flow rate of 20 arbitrary units, spray voltage of −3.5 kV, capillary temperature of 350 °C, S-lens RF level of 30, and mass resolution of 30,000. Measured SRM transitions (*m/*z 133→ 115, 173→ 85, 145→ 101, 191→ 111, 117→ 73, 131→ 85, and 115→ 71).

### Maize tissue samples for DESI-MSI

Maize B73 seeds were prepared for germination by soaking in distilled water for 4 h at room temperature. Tip caps were then excised using a scalpel, and the seeds were sterilized with a 20% bleach solution. Seeds were incubated on damp germination paper inside a plastic petri dish.

Two days after germination, the primary root tip was cut, flash-frozen in liquid nitrogen, and mounted in optimal cutting temperature (O.C.T.) compound. 20 μm-thick tissue sections were cut with a cryostat and thaw-mounted onto a glass slide. While use of O.C.T. has been reported to cause ion suppression due to polyethylene glycol (PEG) and benzalkonium salts in the positive ion mode^50,51^, such suppression effects in the negative ion mode have not been observed by us or others^52^ (Supplementary Fig. 3). Slides were stored in a −80 °C freezer until imaging. Prior to imaging, slides were thawed in a vacuum desiccator.

### Optical microscopy images for DESI-MSI

Brightfield microscopy images of tissue sections were acquired prior to DESI-MS imaging at 250x magnification using a Pluggable USB 2.0 Digital Microscope with LED illumination (Redmond, WA). The contrast of brightfield images has been adjusted for ease of visualization and interpretation.

### DESI-MS imaging

There were two probes and methods used to acquire DESI images of roots in this study: an initial lower resolution system and a subsequent higher resolution system. Initial, lower resolution DESI images of maize roots were acquired with a method and probe described previously^19,53^ and in detail below. However, that probe and imaging parameters poorly resolved lateral features of the root (Supplementary Fig. 1c). To address this, modifications to the DESI probe (Supplementary Fig. 1a, b, d) were made, along with adjustments to the pixel size and ion injection time to obtain higher resolution images. All ten root sections imaged for biological analysis in this study were acquired with the new probe and parameters.

The first, lower resolution images were acquired as follows. The DESI probe was lab-built with a 1/16'' stainless-steel T-junction (Supplementary Fig. 1a). The nebulizer tip consisted of two concentric fused silica capillaries: an outer 350/250 μm (O.D./I.D.) sheath gas capillary and an inner solvent capillary of 150/50 μm (O.D./I.D.). Capillaries were hand-cut with a ceramic stone, and great care was taken to ensure an even, blunt end. The sheath gas and solvent capillaries were both secured in the T-junction using graphite or graphite/Vespel ferrules (Supplementary Figs. 1a, c). The solvent capillary extended continuously from the nebulizer tip, through the T-junction, and ended in a high-pressure PEEK union assembly with Fingertight ferrules (1/16'' tubing O.D. with 0.010'' I.D. thru hole, 10-32). The solvent capillary was secured into one end of the PEEK union with a NanoTight sleeve (1/16'' O.D. x 0.007'' I.D.). A short length of PFA tubing (1/16'' O.D. x 0.030'' I.D.) was secured in the other end of the union and served as the inlet for the solvent syringe.

A histologically compatible solvent mixture of 1:1 (v/v) dimethyl-formamide:acetonitrile at a flow rate of 1 μL/min was used^54^. This solvent mixture provided good signal to background ratios while also preserving tissue integrity. Nitrogen sheath gas was set at 160 psi to aid nebulization. Images were acquired in the negative ion mode with a spray voltage of −5 kV applied at the syringe needle. The probe tip was angled at 56° from the horizontal plane and positioned ~2 mm above the slide surface and ~8 mm away from the extended ion transfer tube.

Images were acquired using a custom-built moving stage coupled to a high-resolution LTQ Orbitrap XL mass spectrometer (Thermo Fisher). Mass resolution was 60,000 (FWHM at m/z 400) with the orbitrap as the mass analyzer, scanning over the mass range 50–1000. The ion injection time was 100 ms per microscan, and the automatic gain control was off, as was previously established by Wiseman et al.^2,55^ and Ifa et al.^56^. One full scan corresponded to one pixel. The pixel size was 114.6 × 130 μm. The pixel height was determined by the vertical step size, and the pixel width was calculated from the cycle time per scan and the lateral speed of the moving stage (120.37 μm/s). The capillary voltage was −65 V; the tube lens voltage was −120 V; and the capillary temperature was 275 °C.

To obtain the higher resolution images reported in this study for the ten root sections (across three biological replicates, Supplementary Table 1), the following modifications to the above-described probe and imaging method were made. The modified DESI-MS probe used an inner solvent capillary of 150/20 μm (O.D./I.D.). The sheath gas capillary was stabilized with a NanoTight sleeve (1/16'' O.D. x 0.0155'' I.D.) and stainless-steel ferrule, whereas the solvent capillary was secured with a graphite ferrule (Supplementary Fig. 1a, b, d). The nitrogen sheath gas was set to 140 psi. The ion injection time was 500 ms per microscan, and the automatic gain control remained off. The pixel size was 72.6 × 80 μm, calculated as described above with a lateral speed of the moving stage of 53.69 μm/sec. The probe angle and height were the same, but the distance to the extended ion transfer tube was ~6 mm.

In order to reduce batch effects, the ten B73 root tissues were imaged over two sequential days. The DESI probe was set-up and the spray optimized for the 1st day of imaging. The sprayer remained in place on the moving stage through the 2nd day of imaging for continuity. The orbitrap was calibrated the day before imaging to ensure mass accuracy.

### DESI-MSI data analysis

Spectra were acquired in Xcalibur 2.5.5 (Thermo Fisher Scientific), where each row of the image was a separate .raw file. The .raw files were converted into .mzML files using MSConvert (a ProteoWizard tool)^57,58^. All .mzML files from an individual tissue section were then compiled into one .imzML file using imzMLConverter (version 1.3, provided with MSiReader)^59^. DESI-MS images were visualized in MSiReader v1.03^60,61^. All images were normalized as a percentage of the total ion current (TIC). The viridis color scheme was used, where deep purple is the least intense and yellow is the most intense. The *m/z* tolerance of the images was set to ±5 ppm to match the expected mass accuracy of the orbitrap. Brightfield microscopy images were overlaid onto the MS images using MSiReader. The transparency was adjusted in MSiReader, and the black background of the optical image was removed in Paint3D (Microsoft) so as not to overly obscure the MS data. This method provided semi-quantitative analysis of intra-root variation in the TIC normalized intensity of various small metabolites and lipids. The TIC can be susceptible to matrix effects, so we have provided reference MS images of the TIC by pixel (Supplementary Fig. 31). Unnormalized MSI of all observed TCA metabolites are also included to demonstrate that normalization did not alter the observed distribution patterns from those observed without normalization (Supplementary Figs. 21–30; odd TIC normalized, even unnormalized).

To calculate the average intensity of aconitate and succinate along the developmental axis, grayscale DESI-MS images of each ion were uploaded to FIJI (open source package, ImageJ)^62^. All images were normalized to the total ion current and scaled to the same maximum intensity. A slice was drawn through the center of each root, and the average intensity across a width of ten pixels was calculated for the length of the root. The average slices from the ten root sections were then averaged and reported (Fig. 2c).

The average spectra of the root tip, meristem, and differentiating zone were obtained by averaging scans across one row of one tissue in each respective developmental zone and subtracting the background (Supplementary Fig. 3a–d). The background spectrum, averaged from eight scans on the adjacent glass slide in each row, was subtracted from each on-tissue average spectrum in Thermo Xcalibur Qual Browser 2.2 (Thermo Scientific).

### DESI-MSI resolution calculations

Lateral resolution was calculated using the 80-20% rule, which has been previously described for MS imaging^63–65^. Briefly, this method defines resolution as the distance over which the signal rises from 20 to 80% of the maximum at an edge. A single ion chromatogram is extracted for these calculations. Here, phosphatidylglycerol (16:0/18:2) was chosen for the resolution calculations based on its signal strength and proximity to the tissue edge. It should be noted that any biological gradient in the selected ion may broaden the calculated resolution.

Single ion chromatograms were exported to Microsoft Excel. Time was converted to distance using the lateral speed of the moving stage, and a straight line was fit to the rising- and falling-edge using measured data points (Supplementary Fig. 2a)^19^. The point-slope formula was then used to calculate the *x*-coordinates of the 20% and 80% intensities. The difference in these coordinates represents the lateral spatial resolution. We calculated the rising- and falling-edge resolution and found there to be no significant difference in the means at a significance level of 0.05 using a two-tailed, two-sample Student’s *t*-test with equal variance (Supplementary Fig. 2b). There was much intra-tissue variability in the measured resolution (Supplementary Fig. 2c, d), which suggested a lack of correlation in calculated values from each tissue section. To account for this, we included data from four rows of each of the ten tissue sections when calculating the average for each edge, resulting in an *n*-value of 40 for the rising- and falling-edge, respectively. This encompasses the total number of calculated resolution values, including multiple per tissue section. Statistical analysis, box plots, and half-violin plots were constructed in OriginPro 2021.

### Identification of molecules by ESI-MS/MS

To definitively identify peaks of interest, we used collision-induced dissociation (CID) of tissue extract. Primary root tips, 0.5 cm in length, were excised from B73 maize shoots 5 days after germination. After patting dry, two root tips were placed in each Eppendorf tube on dry ice. Roots were vacuum desiccated for 18 min, and then ground with a plastic pestle. 350 μL of methanol were added, and then tubes were shaken for 45 min (Analog Vortex Mixer, Ohaus) and subsequently spun down at 3300 rpm. The supernatant was removed and filtered through a 0.2 μm PES filter into a new Eppendorf tube. The filter was rinsed with an additional 100 μL of methanol. Tissue extracts were prepared on the same day as analysis and kept on dry ice until CID was performed.

For electrospray ionization (ESI), a commercial Ion Max source with heated ESI spray tip was used with a high-resolution Orbitrap Elite mass spectrometer (Thermo Fisher). The solution flow rate was set to 5 μL/min. The heater temperature was set to 70 °C, the sheath and auxiliary gas flow rates to 12 AU, the capillary temperature to 325 °C, and the S-Lens RF Level to 60%. Data were acquired in the negative ion mode with a spray voltage of −3 kV. The orbitrap was used as mass analyzer with a mass resolution of 120,000. Normalized collision energy ranged from 15–30%.

These parameters were used for all collected MS/MS spectra, except the fragmentation patterns of PG(16:0/16:0) and PG(16:0/18:2), which were obtained on a different day and instrument^19^. These spectra were acquired from a single flash-frozen maize primary B73 primary root tip. The root was thawed in a vacuum desiccator in an opened Eppendorf tube. A disposable scalpel was used to smash the root tip, and 400 μL methanol were added to the Eppendorf, rinsing the scalpel blade. The sample was allowed to stand for 45 min with periodic vortexing. After centrifuging the sample for 1 min at 3300 rpm, the supernatant was carefully drawn into a syringe without filtering. The sample was sprayed through a lab-built ESI sprayer. In brief, this spray probe consisted of a 1/16'' Swagelok T-junction, where the spray capillary was 365/100 μm (O.D./I.D.) fused silica and the sheath gas capillary was stainless steel. The spray tip was adjusted ~13 mm from the inlet. The nitrogen sheath gas pressure was set to 120 psi, and the solution flow rate to 5 μL/min. Collision-induced dissociation spectra were acquired on the LTQ Orbitrap XL (Thermo Fisher) using the instrument settings described above for the lower resolution DESI imaging. The normalized collision energy was 25-30%.

Collected fragmentation patterns were compared to database values obtained from METLIN^66,67^, METLIN Gen2, LipidMaps^68^, and PubChem^69^, as well as previously published literature data^21,70^. A table of major fragment peaks for all MSI in the main text is provided in Supplementary Table 2. Full fragmentation spectra are provided in the Supplementary Information (Supplementary Figs. 32–50).

### Statistics and reproducibility

DESI-MSI experiments were conducted on three biological replicates, with two to four technical replicates each, for a total of ten sections. These were imaged over two sequential days. A table describing the replicates and sequencing of imaging is provided (Supplementary Table 1). All statistical analysis methods for individual experiments are described in the text and in the figures. For all experiments described, no data were excluded from the analyses. No statistical method was used to predetermine sample size. Randomization and blinding were not used during experiments and outcome assessment. All statistics were analyzed using GraphPad Prism (version 9) or Origin Pro 2021.

### Reporting summary

Further information on research design is available in the Nature Portfolio Reporting Summary linked to this article.

## Supplementary information

Supplementary Information

Reporting Summary

## Data Availability

All mass spectrometry imaging files (raw and derived), associated ESI-MS/MS files, and HPLC-MS/MS files generated in this study have been deposited in the Figshare database under accession code 10.6084/m9.figshare.22350886^71^. Source data are provided with this paper.

## Source data

Source Data

## References

[1] Moussaieff A (2013). High-resolution metabolic mapping of cell types in plant roots. Proc. Natl Acad. Sci. USA.

[2] Wiseman JM, Ifa DR, Song Q, Cooks RG (2006). Tissue imaging at atmospheric pressure using Desorption Electrospray Ionization (DESI) mass spectrometry. Angew. Chem. Int. Ed..

[3] Ifa DR (2011). Tissue imprint imaging by desorption electrospray ionization mass spectrometry. Anal. Methods.

[4] Thunig J, Hansen SH, Janfelt C (2011). Analysis of secondary plant metabolites by indirect desorption electrospray ionization imaging mass spectrometry. Anal. Chem..

[5] Li B, Bjarnholt N, Hansen SH, Janfelt C (2011). Characterization of barley leaf tissue using direct and indirect desorption electrospray ionization imaging mass spectrometry. J. Mass Spectrom..

[6] Hemalatha RG, Pradeep T (2013). Understanding the molecular signatures in leaves and flowers by Desorption Electrospray Ionization Mass Spectrometry (DESI MS) imaging. J. Agric. Food Chem..

[7] Freitas JRLE, Vendramini PH, Melo JOF, Eberlin MN, Augusti R (2019). Assessing the spatial distribution of key flavonoids in *Mentha × piperita* leaves: an application of Desorption Electrospray Ionization Mass Spectrometry Imaging (DESI-MSI). J. Braz. Chem. Soc..

[8] Kumara PM (2016). Desorption Electrospray Ionization (DESI) mass spectrometric imaging of the distribution of rohitukine in theseedling of dysoxylum binectariferum hook. F. PLoS One.

[9] Mohana Kumara P, Uma Shaanker R, Pradeep T (2019). UPLC and ESI-MS analysis of metabolites of Rauvolfia tetraphylla L. and their spatial localization using desorption electrospray ionization (DESI) mass spectrometric imaging. Phytochemistry.

[10] Liao Y (2019). Visualized analysis of within-tissue spatial distribution of specialized metabolites in tea (Camellia sinensis) using desorption electrospray ionization imaging mass spectrometry. Food Chem..

[11] Müller T, Oradu S, Ifa DR, Cooks RG, Kräutler B (2011). Direct plant tissue analysis and imprint imaging by Desorption Electrospray Ionization Mass Spectrometry. Anal. Chem..

[12] Li B, Hansen SH, Janfelt C (2013). Direct imaging of plant metabolites in leaves and petals by desorption electrospray ionization mass spectrometry. Int. J. Mass Spectrom..

[13] Li B (2013). Visualizing metabolite distribution and enzymatic conversion in plant tissues by desorption electrospray ionization mass spectrometry imaging. Plant J..

[14] Gerbig S, Brunn HE, Spengler B, Schulz S (2015). Spatially resolved investigation of systemic and contact pesticides in plant material by desorption electrospray ionization mass spectrometry imaging (DESI-MSI). Anal. Bioanal. Chem..

[15] Feenstra AD, Dueñas ME, Lee YJ (2017). Five micron high resolution MALDI mass spectrometry imaging with simple, interchangeable, multi-resolution optical system. J. Am. Soc. Mass Spectrom..

[16] O’Neill KC, Lee YJ (2020). Visualizing genotypic and developmental differences of free amino acids in maize roots with mass spectrometry imaging. Front. Plant Sci..

[17] Forsman TT, Dueñas ME, Lee YJ (2021). On-tissue boronic acid derivatization for the analysis of vicinal diol metabolites in maize with MALDI-MS imaging. J. Mass Spectrom..

[18] Sarabia LD (2018). High-mass-resolution MALDI mass spectrometry imaging reveals detailed spatial distribution of metabolites and lipids in roots of barley seedlings in response to salinity stress. Metabolomics.

[19] Noll, S. E. *Insights Into Metabolism And Signaling at Small Scale*. https://purl.stanford.edu/dx038hq9888 (2021).

[20] Zhou S, Richter A, Jander G (2018). Beyond defense: multiple functions of Benzoxazinoids in maize metabolism. Plant Cell Physiol..

[21] de Bruijn WJC, Vincken J-P, Duran K, Gruppen H (2016). Mass spectrometric characterization of Benzoxazinoid glycosides from Rhizopus-elicited wheat (Triticum aestivum) seedlings. J. Agric. Food Chem..

[22] Hieta J-P, Sipari N, Räikkönen H, Keinänen M, Kostiainen R (2021). Mass spectrometry imaging of Arabidopsis thaliana leaves at the single-cell level by infrared laser ablation atmospheric pressure photoionization (LAAPPI). J. Am. Soc. Mass Spectrom..

[23] Tang M (2021). A genome‐scale TF–DNA interaction network of transcriptional regulation of *Arabidopsis* primary and specialized metabolism. Mol. Syst. Biol..

[24] Li S, Yamada M, Han X, Ohler U, Benfey PN (2016). High-resolution expression map of the Arabidopsis root reveals alternative splicing and lincRNA regulation. Dev. Cell.

[25] Carey BW, Finley LWS, Cross JR, Allis CD, Thompson CB (2015). Intracellular α-ketoglutarate maintains the pluripotency of embryonic stem cells. Nature.

[26] Zhao Y (2018). Malate transported from chloroplast to mitochondrion triggers production of ROS and PCD in Arabidopsis thaliana. Cell Res..

[27] Sato S, Yanagisawa S (2014). Characterization of metabolic states of Arabidopsis thaliana under diverse carbon and nitrogen nutrient conditions via targeted metabolomic analysis. Plant Cell Physiol..

[28] Mora-Macías J (2017). Malate-dependent Fe accumulation is a critical checkpoint in the root developmental response to low phosphate. Proc. Natl Acad. Sci. USA.

[29] Zheng Z, Wang Z, Wang X, Liu D (2019). Blue light-triggered chemical reactions underlie phosphate deficiency-induced inhibition of root elongation of Arabidopsis seedlings grown in petri dishes. Mol. Plant.

[30] Zhang Y (2021). Two mitochondrial phosphatases, PP2c63 and Sal2, are required for posttranslational regulation of the TCA cycle in Arabidopsis. Mol. Plant.

[31] Tal L (2022). A conformational switch in the SCF-D3/MAX2 ubiquitin ligase facilitates strigolactone signalling. Nat. Plants.

[32] Colón-Carmona A, You R, Haimovitch-Gal T, Doerner P (1999). Spatio-temporal analysis of mitotic activity with a labile cyclin–GUS fusion protein. Plant J..

[33] Yi D (2014). The Arabidopsis SIAMESE-RELATED cyclin-dependent kinase inhibitors SMR5 and SMR7 regulate the DNA damage checkpoint in response to reactive oxygen species. Plant Cell.

[34] De Col V (2017). ATP sensing in living plant cells reveals tissue gradients and stress dynamics of energy physiology. eLife.

[35] Dunand C, Crèvecoeur M, Penel C (2007). Distribution of superoxide and hydrogen peroxide in Arabidopsis root and their influence on root development: possible interaction with peroxidases. N. Phytol..

[36] Tsukagoshi H, Busch W, Benfey PN (2010). Transcriptional regulation of ROS controls transition from proliferation to differentiation in the root. Cell.

[37] Yamada M, Han X, Benfey PN (2020). RGF1 controls root meristem size through ROS signaling. Nature.

[38] Zhou L (2018). Exogenous hydrogen peroxide inhibits primary root gravitropism by regulating auxin distribution during Arabidopsis seed germination. Plant Physiol. Biochem.

[39] Jardim-Messeder D (2015). Succinate dehydrogenase (mitochondrial complex II) is a source of reactive oxygen species in plants and regulates development and stress responses. N. Phytologist.

[40] Quinlan CL (2012). Mitochondrial complex II can generate reactive oxygen species at high rates in both the forward and reverse reactions. J. Biol. Chem..

[41] Detraux D, Renard P (2022). Succinate as a new actor in pluripotency and early development?. Metabolites.

[42] Ko SH (2017). Succinate promotes stem cell migration through the GPR91-dependent regulation of DRP1-mediated mitochondrial fission. Sci. Rep..

[43] Grimolizzi F, Arranz L (2018). Multiple faces of succinate beyond metabolism in blood. Haematologica.

[44] Martínez-Reyes I, Chandel NS (2020). Mitochondrial TCA cycle metabolites control physiology and disease. Nat. Commun..

[45] Pracharoenwattana I, Cornah JE, Smith SM (2005). Arabidopsis peroxisomal citrate synthase is required for fatty acid respiration and seed germination. Plant Cell.

[46] Pracharoenwattana I (2010). Arabidopsis has a cytosolic fumarase required for the massive allocation of photosynthate into fumaric acid and for rapid plant growth on high nitrogen. Plant J..

[47] Hooks MA (2014). Selective induction and subcellular distribution of ACONITASE 3 reveal the importance of cytosolic citrate metabolism during lipid mobilization in Arabidopsis. Biochem. J..

[48] Gao X, Chen J, Dai X, Zhang D, Zhao Y (2016). An effective strategy for reliably isolating heritable and Cas9-free Arabidopsis mutants generated by CRISPR/Cas9-mediated genome editing. Plant Physiol..

[49] Kumar D, Yusuf MA, Singh P, Sardar M, Sarin NB (2014). Histochemical detection of superoxide and H2O2 accumulation in Brassica juncea seedlings. Bio-Protoc..

[50] Schwartz SA, Reyzer ML, Caprioli RM (2003). Direct tissue analysis using matrix-assisted laser desorption/ionization mass spectrometry: practical aspects of sample preparation. J. Mass Spectrom..

[51] Berry KAZ (2011). MALDI imaging MS of phospholipids in the mouse lung[S]. J. Lipid Res..

[52] Goto T (2014). The expression profile of phosphatidylinositol in high spatial resolution imaging mass spectrometry as a potential biomarker for prostate cancer. PLoS One.

[53] Armstrong N (2022). SDHB knockout and succinate accumulation are insufficient for tumorigenesis but dual SDHB/NF1 loss yields SDHx-like pheochromocytomas. Cell Rep..

[54] Eberlin LS (2011). Nondestructive, histologically compatible tissue imaging by desorption electrospray ionization mass spectrometry. ChemBioChem.

[55] Wiseman JM (2008). Desorption electrospray ionization mass spectrometry: Imaging drugs and metabolites in tissues. Proc. Natl Acad. Sci..

[56] Ifa DR, Wiseman JM, Song Q, Cooks RG (2007). Development of capabilities for imaging mass spectrometry under ambient conditions with desorption electrospray ionization (DESI). Int. J. Mass Spectrom..

[57] Kessner D, Chambers M, Burke R, Agus D, Mallick P (2008). ProteoWizard: open source software for rapid proteomics tools development. Bioinformatics.

[58] Chambers MC (2012). A cross-platform toolkit for mass spectrometry and proteomics. Nat. Biotechnol..

[59] Race AM, Styles IB, Bunch J (2012). Inclusive sharing of mass spectrometry imaging data requires a converter for all. J. Proteom..

[60] Bokhart MT, Nazari M, Garrard KP, Muddiman DC (2018). MSiReader v1.0: Evolving open-source mass spectrometry imaging software for targeted and untargeted analyses. J. Am. Soc. Mass Spectrom..

[61] Robichaud G, Garrard KP, Barry JA, Muddiman DC (2013). MSiReader: an open-source interface to view and analyze high resolving power MS imaging files on Matlab platform. J. Am. Soc. Mass Spectrom..

[62] Schindelin J (2012). Fiji: an open-source platform for biological-image analysis. Nat. Methods.

[63] Laskin J, Heath BS, Roach PJ, Cazares L, Semmes OJ (2012). Tissue imaging using nanospray desorption electrospray ionization mass spectrometry. Anal. Chem..

[64] Luxembourg SL, Mize TH, McDonnell LA, Heeren RMA (2004). High-spatial resolution mass spectrometric imaging of peptide and protein distributions on a surface. Anal. Chem..

[65] Yin R, Burnum-Johnson KE, Sun X, Dey SK, Laskin J (2019). High spatial resolution imaging of biological tissues using nanospray desorption electrospray ionization mass spectrometry. Nat. Protoc..

[66] Smith CA (2005). METLIN: a metabolite mass spectral database. Ther. Drug Monit..

[67] Guijas C (2018). METLIN: A technology platform for identifying knowns and unknowns. Anal. Chem..

[68] Fahy E, Sud M, Cotter D, Subramaniam S (2007). LIPID MAPS online tools for lipid research. Nucleic Acids Res.

[69] Kim S (2021). PubChem in 2021: new data content and improved web interfaces. Nucleic Acids Res.

[70] Bonnington LS, Barcelò D, Knepper TP (2003). Utilisation of electrospray time-of-flight mass spectrometry for solving complex fragmentation patterns: application to benzoxazinone derivatives. J. Mass Spectrom..

[71] Zhang, T. et al. *Datasets for “Chemical Imaging Reveals Diverse Functions of Tricarboxylic Acid Metabolites in Root Growth and Development”*. 10.6084/m9.figshare.22350886 (2023). 10.1038/s41467-023-38150-z PMC10160030 37142569

