# Supplementary material for: Chemical imaging reveals diverse functions of tricarboxylic acid metabolites in root growth and development
Source: Nat Commun. 2023 May 4;14:2567. doi: 10.1038/s41467-023-38150-z (PMC10160030; doi:10.1038/s41467-023-38150-z)
Supplement: Supplementary file 1 — Supplementary Information [file 41467_2023_38150_MOESM1_ESM.pdf]

Supplementary Information for

Chemical Imaging Reveals Diverse Functions of Tricarboxylic Acid Metabolites in Root Growth and Development

Tao Zhang[1#], Sarah E. Noll[2, 3#], Jesus T. Peng[1], Amman Klair[1], Abigail Tripka[1], Nathan Stutzman[1], Casey Cheng[1], Richard N. Zare[2*], Alexandra J. Dickinson[1*]

1. Cell and Developmental Biology, University of California San Diego, La Jolla, CA 92093, USA.
2. Department of Chemistry, Stanford University, Stanford, CA 94305, USA.
3. Department of Chemistry, Pomona College, Claremont, CA 91711, USA.

* To whom correspondence should be addressed: zare@stanford.edu and adickinson@ucsd.edu

# These authors contributed equally to this work.

This PDF file includes:

Supplemental Figures 1-50
Supplemental Tables 1-3

Other supplementary materials include:

Source Data file
Mass spectrometry data files are accessible as described in the Data Availability statement

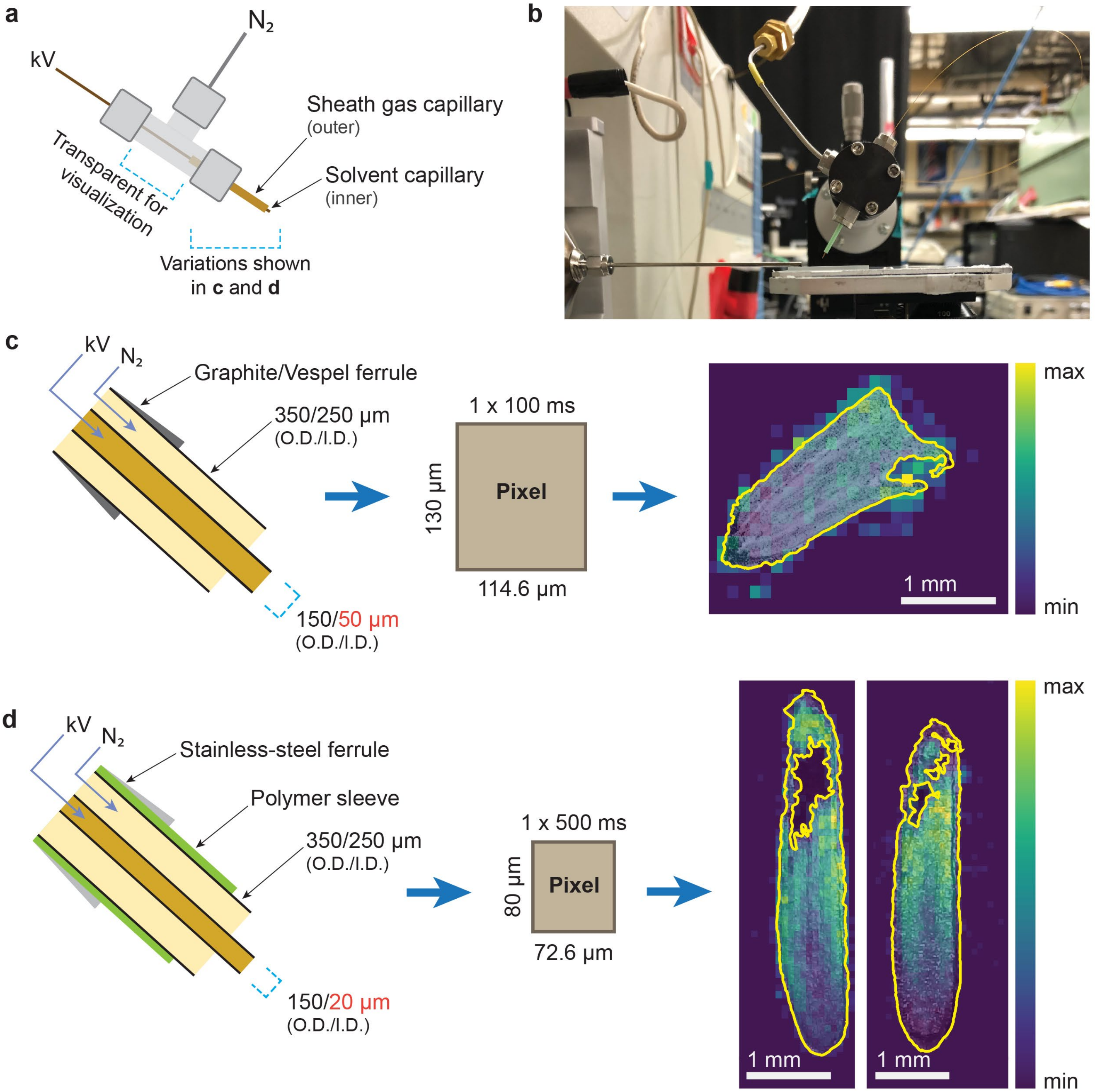

Supplementary Fig. 1 | DESI-MSI probe design and imaging parameters for improved spatial resolution. a Generic schematic of the DESI probe shows the coaxial capillary construction at the tip, where the outer capillary is for the nitrogen sheath gas and the inner capillary runs continuously from the spray tip backward to the solvent syringe. **b** Photograph of the DESI imaging set-up for maize root acquisition. **c, d** Details of the coaxial probe tip, pixel size, ion injection time, and sample DESI-MS images of fumarate are shown for the initial, lower resolution maize imaging attempts (**c**) and the modified probe and parameters used for all higher resolution image acquisition in this study (**d**). MS images are of fumarate in the negative ionization mode with brightfield microscopy image overlay. The outer borders and holes of the brightfield image are outlined in yellow to demonstrate the improved resolution with the modified set-up. Each imaging experiment, resulting in an MS image with microscopy overlay, was replicated three times for (**c**) and ten times for (**d**); one and two representative images are shown, respectively. O.D. is outer diameter, and I.D. is inner diameter.

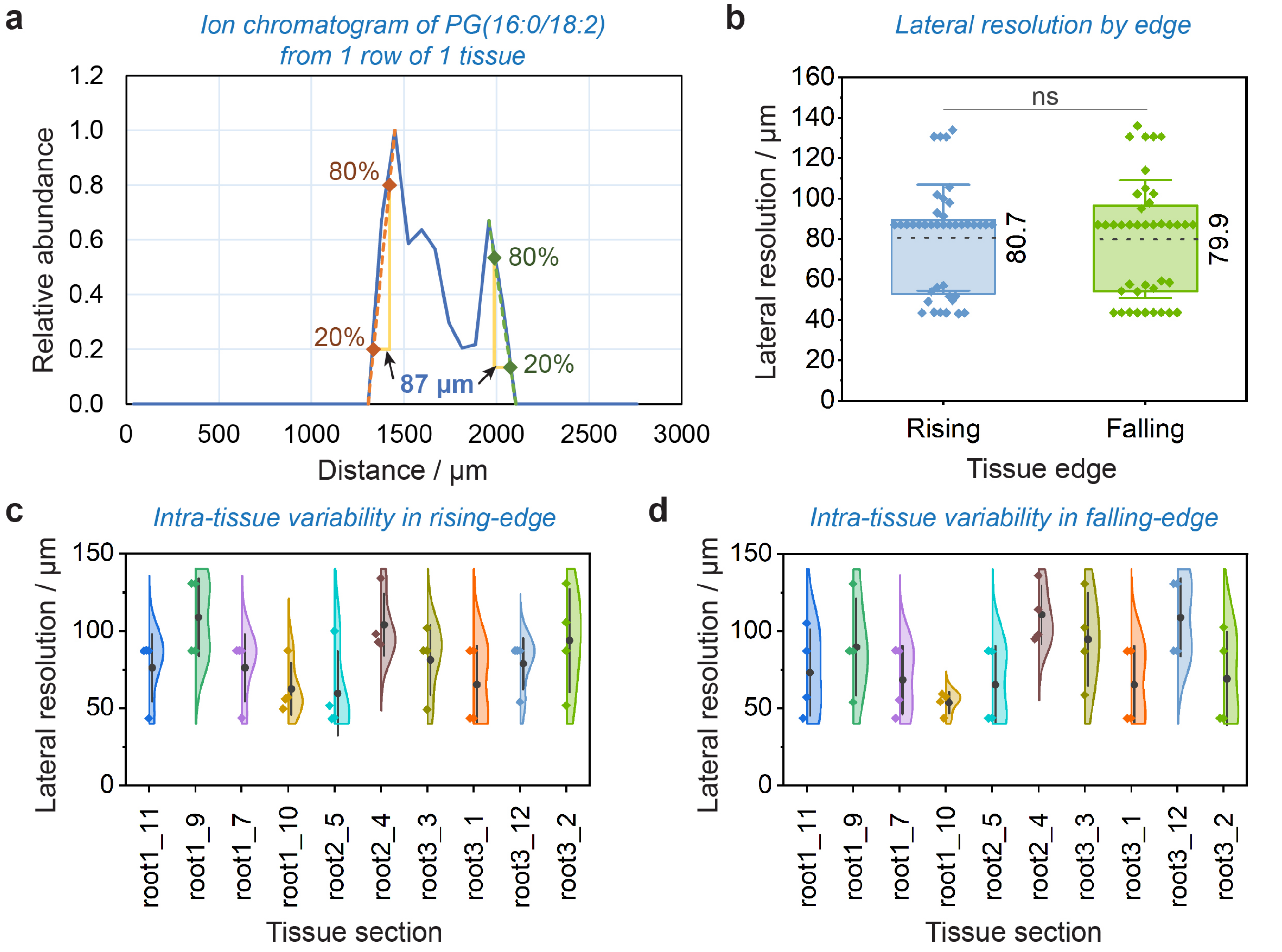

Supplementary Fig. 2 | Lateral resolution of MS images was determined using the 80-20% rule. a Demonstration of the 80-20% rule using the extracted ion chromatogram of PG(16:0/18:2) from one row of one maize tissue in the negative ionization mode. The distance over which the intensity rises from 20% to 80% of the maximum for each edge is the lateral resolution. **b** Lateral resolution for the rising- and falling-edges of the ten tissue sections. Dashed lines and labels show the mean values, while whiskers are one standard deviation. The box encompasses the 25th-75th percentile. Four rows were measured for each of the ten tissue sections (from 3 biologically independent samples), providing n = 40 rows for each edge. A two-tailed, two-sample Student's t-test showed no significant difference in the means at a significance level of 0.05 (P = 0.90). To justify the inclusion of four rows per tissue, we show the intra-tissue variability in the measured lateral resolution for the rising- (**c**) and falling-edge (**d**) of the ten measured tissues (n = 4 rows per tissue section). Dark gray circle and line represent the mean value and one standard deviation for each tissue section. Measured values are colored diamonds. The half-violin plots were constructed with bin widths of 20 µm. Source data are provided as a Source Data file.

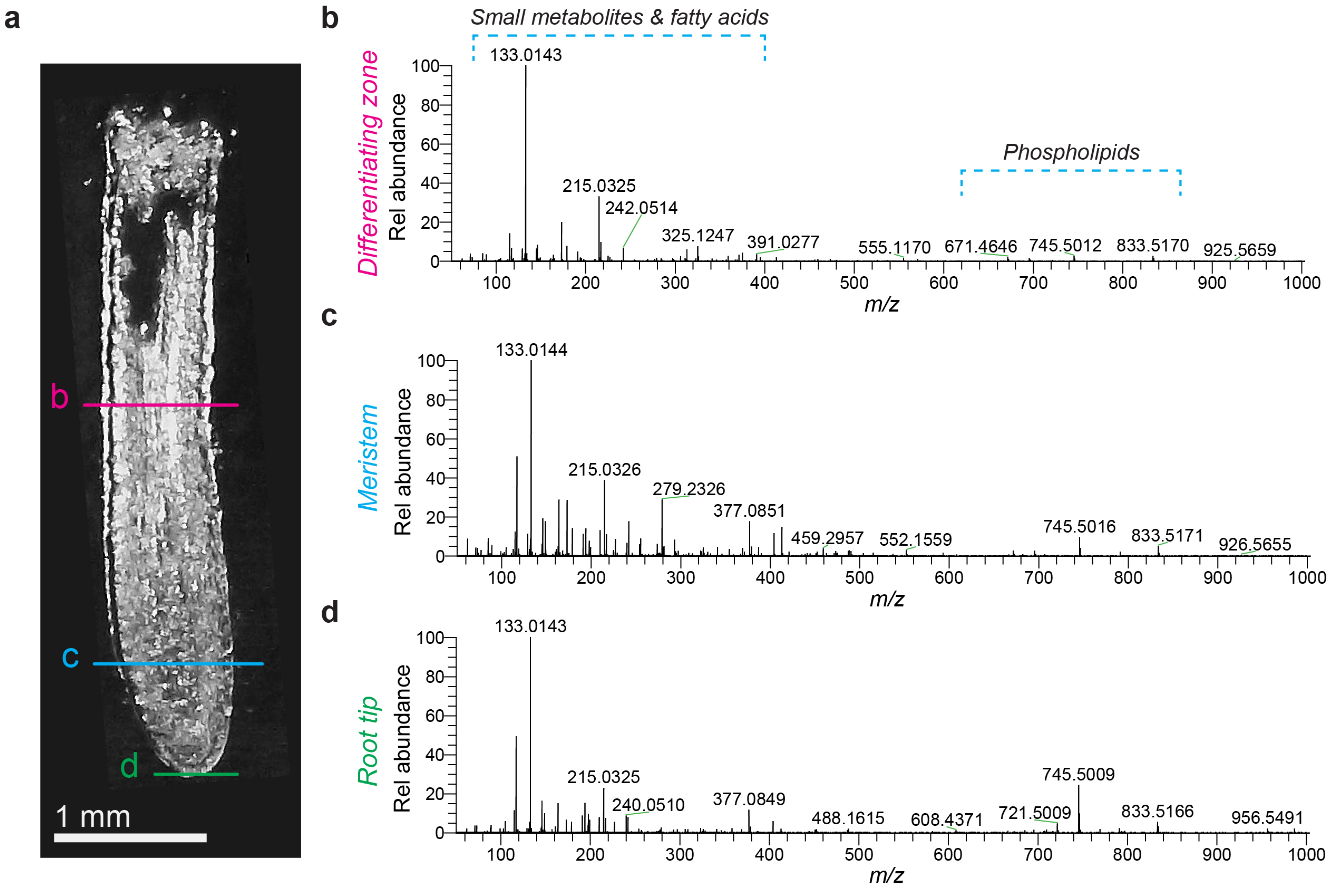

Supplementary Fig. 3 | Representative mass spectra from distinct developmental zones of the maize root. a Brightfield microscopy image of the root section from which a representative, background-subtracted spectrum of the differentiating region (**b**, pink), meristem zone (**c,** blue), and root tip (**d,** green) are shown. The lines in (**a**) indicate the tissue rows that were averaged to produce the negative-mode spectra shown in (**b-d**). The background was determined by averaging across eight scans of the adjacent glass slide in each row (**b-d**). This experiment was replicated ten times across three biological samples (Supplementary Table 1). Data from one representative tissue section are shown here. Source data are provided as a Source Data file; mass spectrometry data files are also accessible as described in the Data Availability statement.

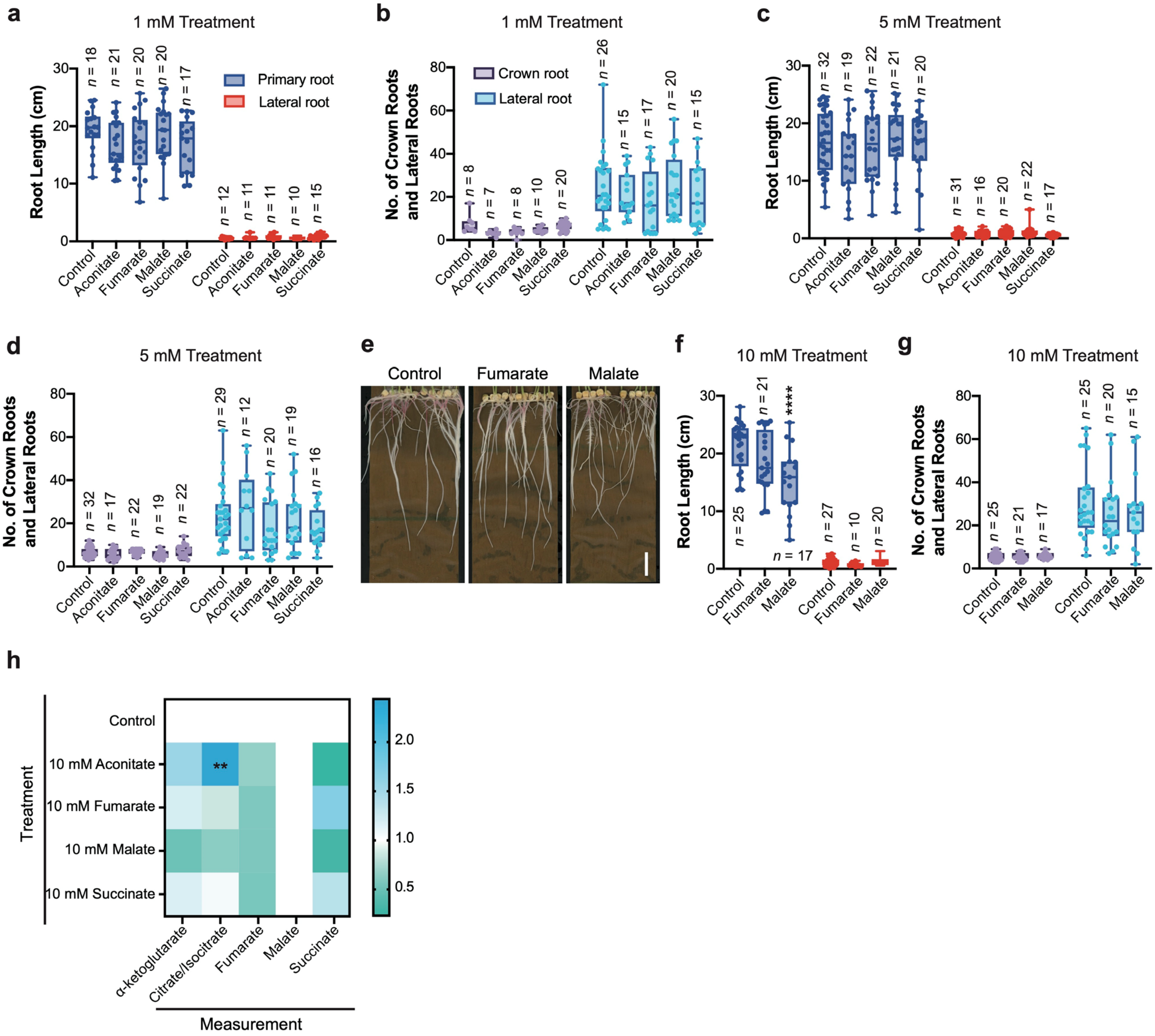

Supplementary Fig. 4 | Maize responds differentially to 10 mM treatments of TCA metabolites. a-**d** Root phenotypes in maize seedlings treated with 1 mM and 5 mM aconitate, fumarate, malate, and succinate, respectively. **e-g** Root phenotypes in maize seedlings treated with 10 mM fumarate and malate. Scale bar, 3 cm. Asterisks indicate statistical significance by one-way ANOVA (*P* = 0.00034, ****P* < 0.0001) in (**g**). **h** Analysis of TCA metabolites using HPLC-MS shows the effect of 10 mM metabolite treatments on the normalized level of five TCA metabolites. Data represent means ± s.d. (*n* = 3 biological replicates). Asterisks indicate statistical significance by two-way ANOVA (*P* = 0.0073, ***P* < 0.01). For the boxplots, each dot represents the datapoint of one biological replicate (exact *n* values provided in each plot); the central line indicates the median; the bounds of the box show the 25th and 75th percentiles; and the whiskers indicate the maximum and minimum values. Source data are provided as a Source Data file.

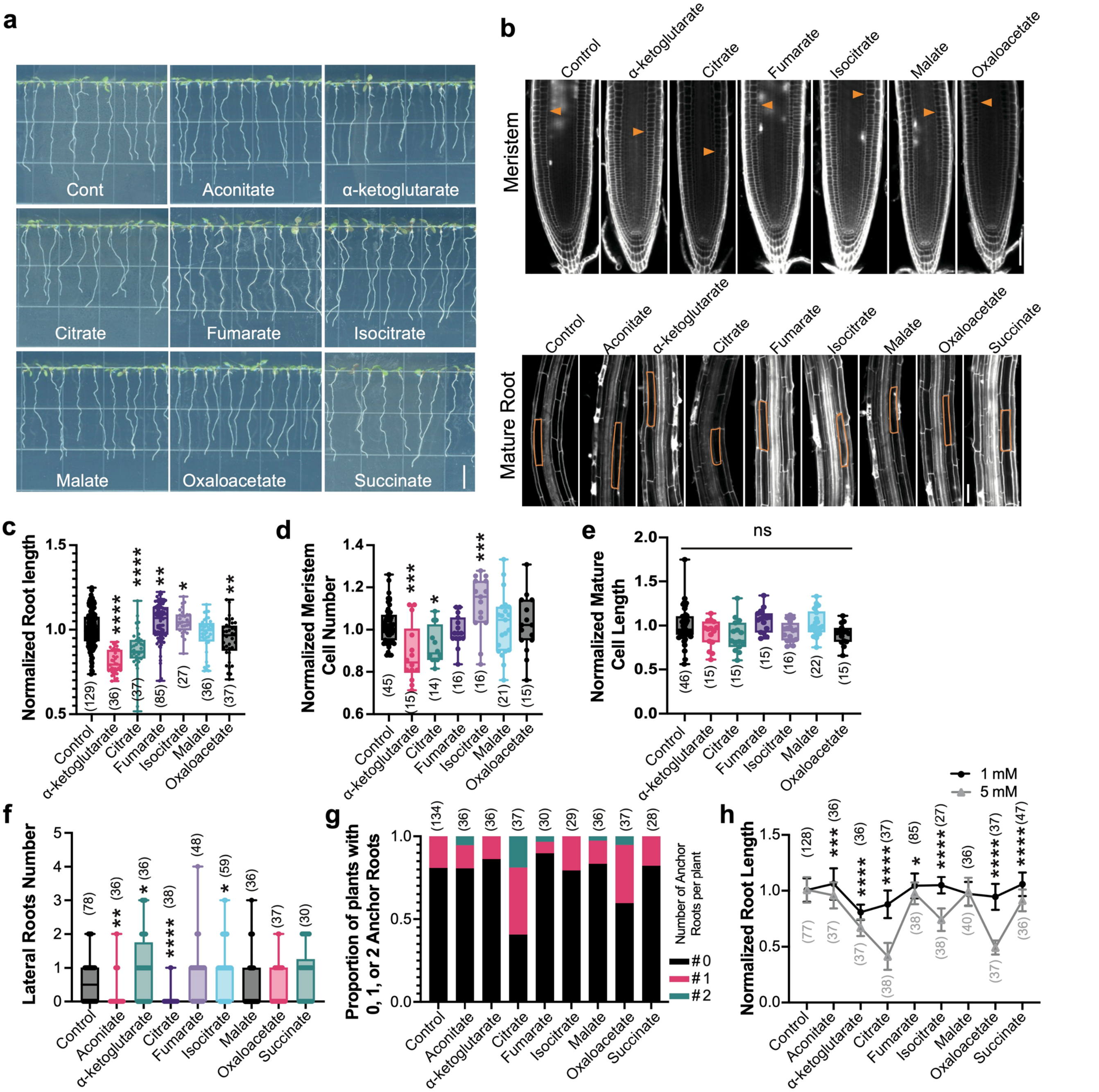

Supplementary Fig. 5 | Characterization of the response of the Arabidopsis root to 1 mM TCA metabolite treatments. a Exogenous 1 mM TCA metabolite treatments showing different effects on plant root development. Scale bar, 1 cm. **b** Confocal images of meristem and differentiation zone cells, respectively, under 1 mM TCA metabolite treatments. The orange arrows indicate the end of the meristem. Scale bar, 50 μm. **c-e** Quantification of the effects of 1 mM treatments on the primary root length (**c**), the number of meristematic cells (**d**), and the average cell length of the mature root (**e**), normalized to the control. For the boxplots, each dot represents the datapoint of one biological replicate; the central line indicates the median; the bounds of the box show the 25th and 75th percentiles; and the whiskers indicate the maximum and minimum values. *n* values are indicated in the graphs. Asterisks indicate statistical significance by two-tailed unpaired Student's *t*-test (*$P < 0.05$, **$P < 0.01$, ***$P < 0.001$, ****$P < 0.0001$) (**c-e**). **f** Average lateral root number (LRN) after 1 mM TCA metabolite treatments. Data represent means ± s.d. (*n* values are indicated in the graphs). Asterisks indicate statistical significance by one-way ANOVA (**$P < 0.01$). **g** The proportion of plants with 0, 1, or 2 anchor roots (ARs) after 1 mM TCA metabolite treatments. *n* values are indicated in the graphs. **h** Concentration-dependent growth of roots under exogenous TCA metabolite treatments. Data are presented as means ± s.d. (*n* values are indicated in black for 1 mM TCA metabolite treatments and in grey for 5 mM TCA metabolite treatments, respectively). Asterisks indicate statistical significance by two-tailed unpaired Student's *t*-test (*$P < 0.05$, **$P < 0.01$, ***$P < 0.001$, ****$P < 0.0001$). For exact *P* values see Source Data. Source data are provided as a Source Data file.

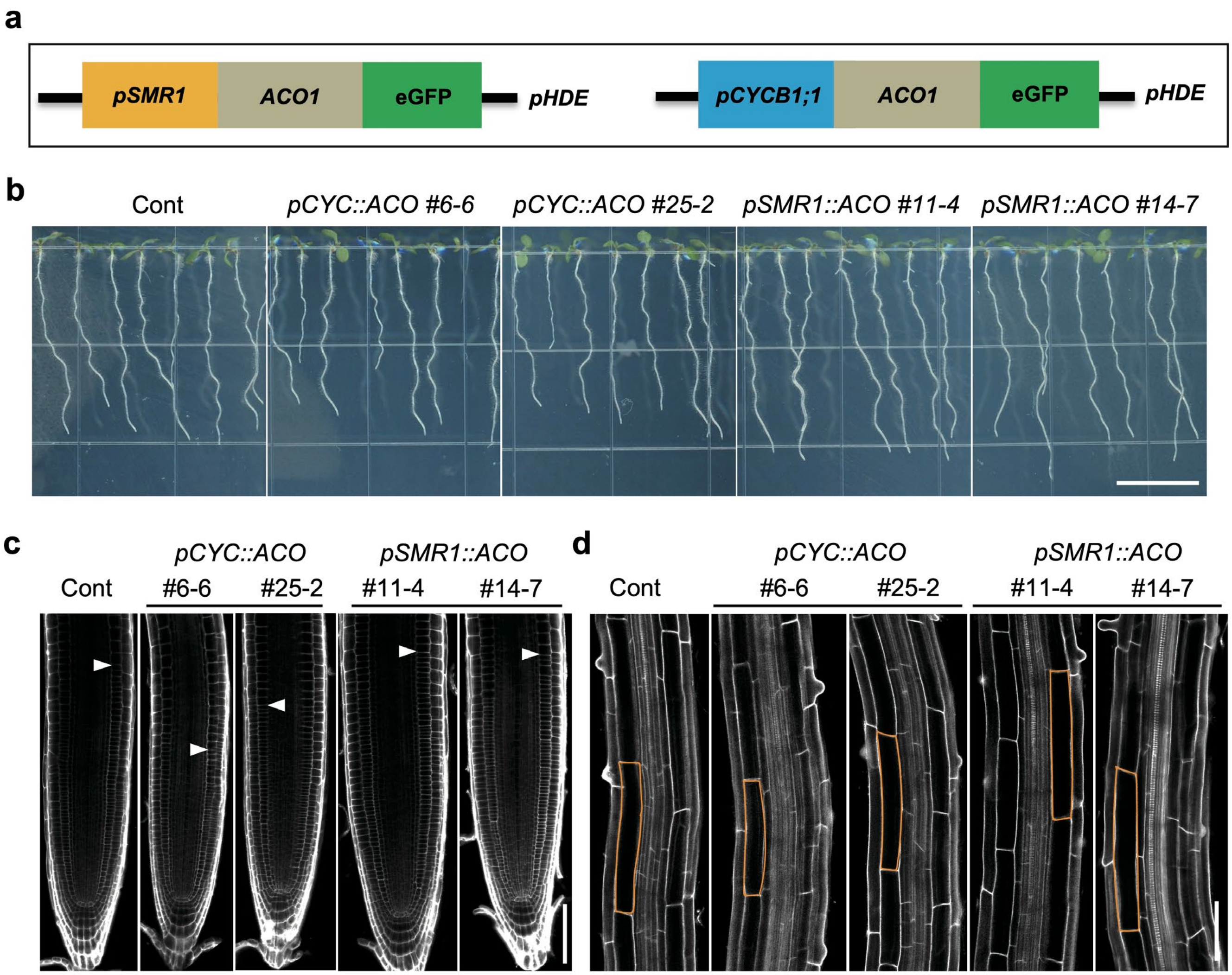

Supplementary Fig. 6 | Ectopic expression of TCA metabolites using a tissue-specific genetic engineering approach in Arabidopsis. a Schematic of constructs for ectopic expression of TCA cycle genes. **b** The phenotype of transgenic lines. Scale bar, 1 cm. The control is a representative negative transgenic line. **c, d** Confocal images of meristem cells and mature root cells, respectively**.** The white arrows indicate the end of the meristem zone. These images were obtained from Fig. 3i. At least three independent experiments were repeated. Scale bars, 100 μm (**c**) and 150 μm (**d**).

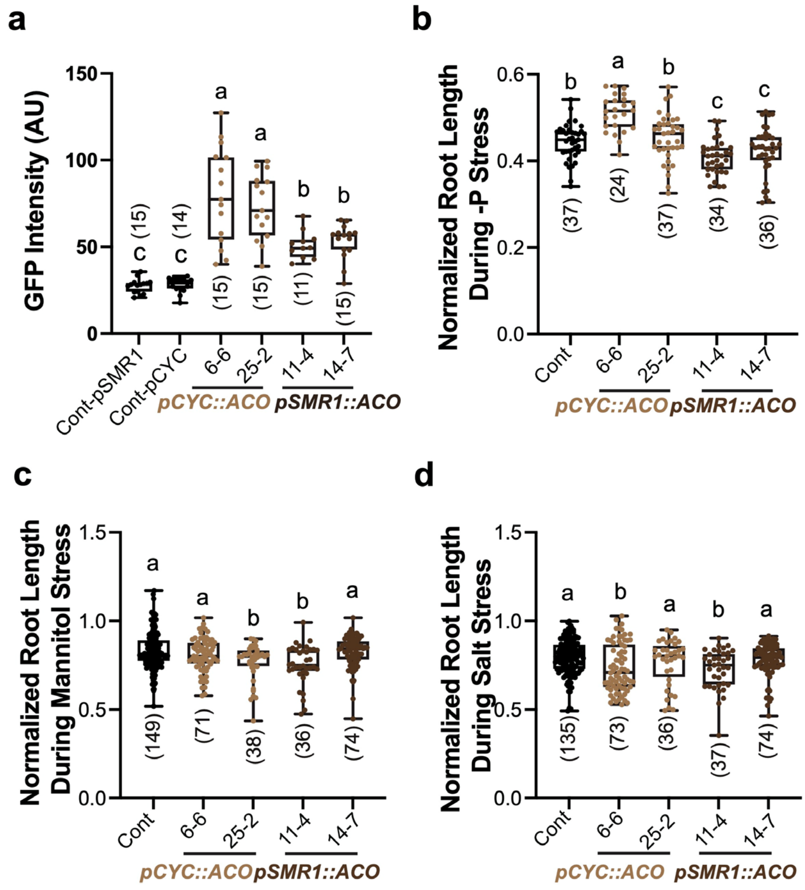

Supplementary Fig. 7 | Ectopic expression of TCA metabolites in transgenic Arabidopsis plants grown under different stress conditions. a GFP intensity of transgenic lines. **b-d** Normalized primary root length of transgenic lines in media without phosphate, media with 100 mM mannitol, and media with 50 mM NaCl. For the boxplots, the central line indicates the median; the bounds of the box show the 25th and 75th percentiles; and the whiskers indicate the maximum and minimum values. Data are presented as boxplots with each dot representing the datapoint of one biological replicate. Bars labeled with the same letters are not significantly different, whereas bars labeled with different letters have a statistically significant difference in the means at $P < 0.05$. n values are indicated in the graphs. All P values were determined by two-tailed unpaired Student's t-test. For exact P values see Source Data. Source data are provided as a Source Data file.

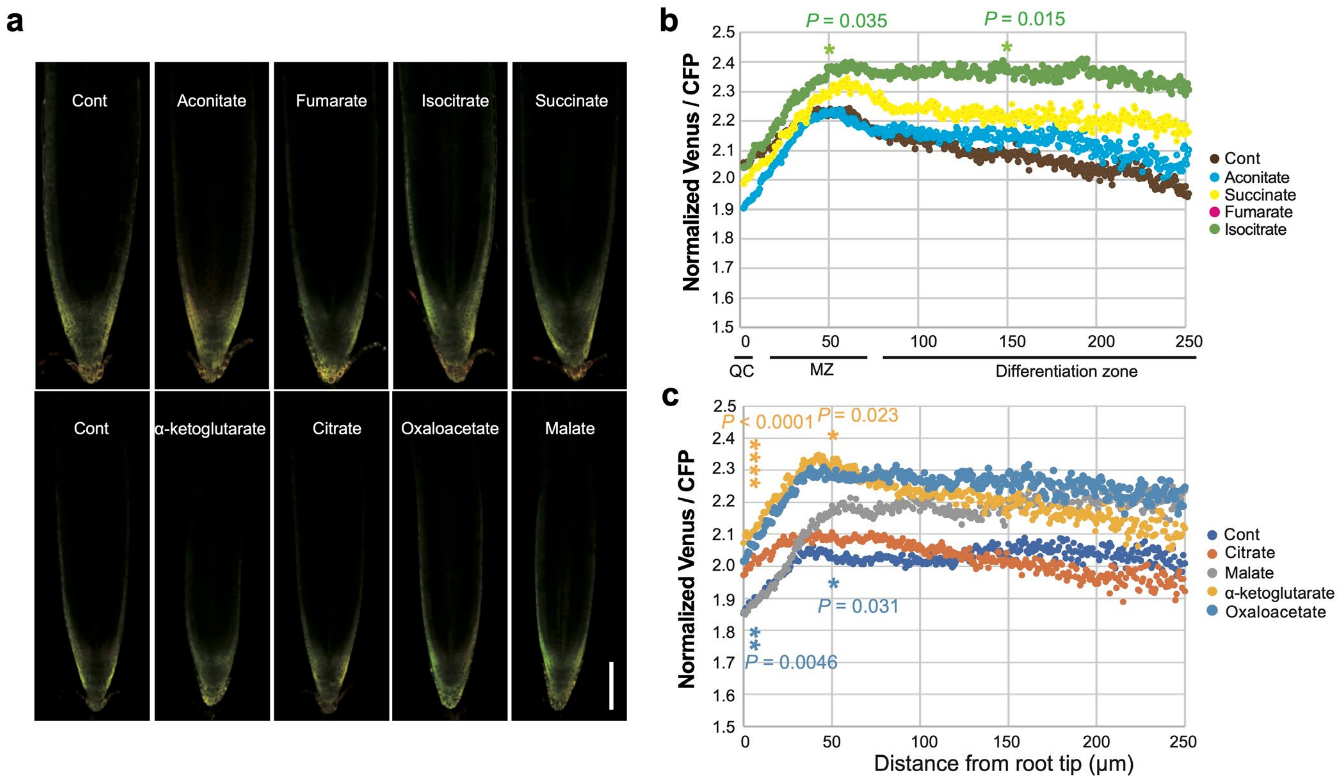

Supplementary Fig. 8 | Response of ATP in Arabidopsis root to 1 mM TCA metabolite treatments. a Merged images of a confocal fluorescence (Venus and CFP) image of ATP sensor under 1 mM TCA metabolite treatments. Scale bar, 100 μm. **b, c** Normalized Venus/CFP intensity of ATP sensor under 1 mM TCA metabolite treatments. Data represent means ± s.d. ($n = 15$ plants). Asterisks indicate statistical significance by two-tailed unpaired Student's *t*-test (*$P < 0.05$, **$P < 0.01$, ***$P < 0.001$, ****$P < 0.0001$, the exact *P* values are also indicated in the graphs). Different colors indicate different TCA metabolite treatments. Source data are provided as a Source Data file.

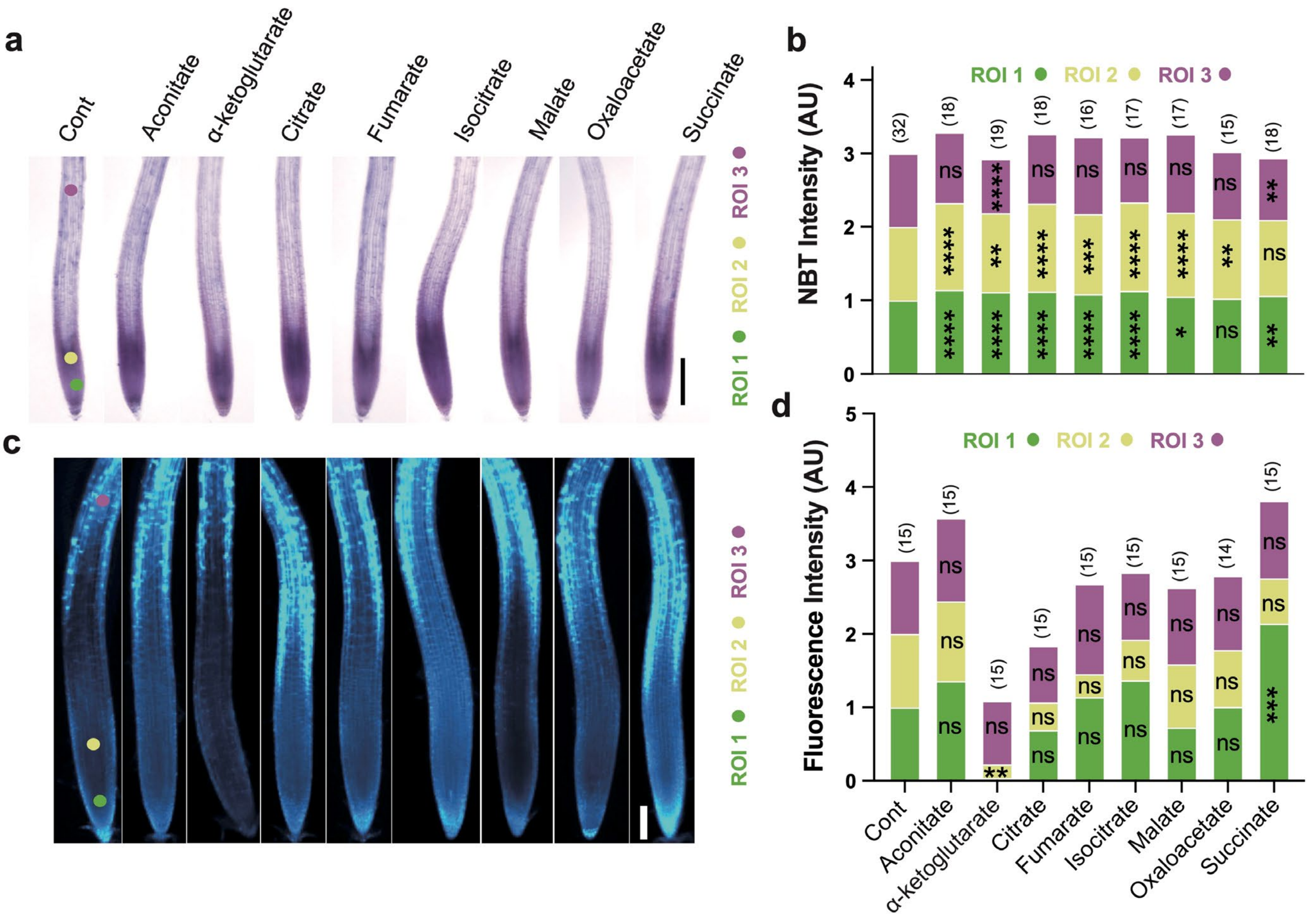

Supplementary Fig. 9 | ROS staining of 1 mM TCA metabolite treatments of Arabidopsis. a Images showing NBT staining. Scale bar, 2 mm. **b** Normalized intensity of NBT stain in three regions of interest (ROIs). **c** Images showing H_2DCFDA staining. Scale bar, 100 µm. **d** H_2DCFDA with 1 mM TCA metabolite treatments in three regions of interest (ROIs). Data represent means (*n* values are indicated in the graphs; s.d. values can be seen in the Source Data). Asterisks indicate statistically significant differences from the control by one-way ANOVA (*$P < 0.05$, **$P < 0.01$, ***$P < 0.001$, ****$P < 0.0001$). For exact *P* values see Source Data. Source data are provided as a Source Data file.

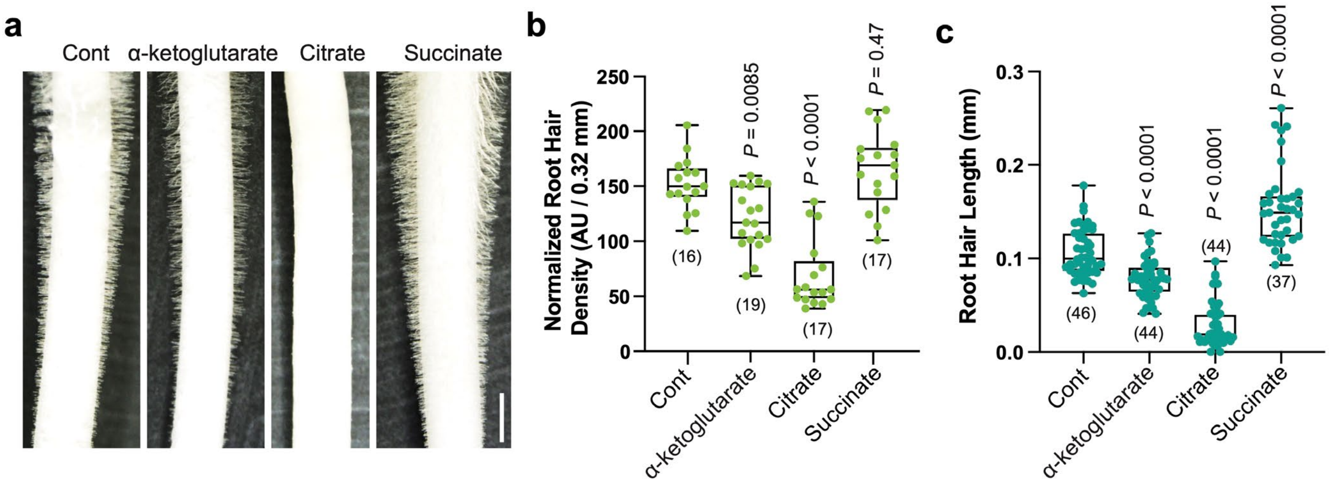

Supplementary Fig. 10 | Root hair phenotypes induced by 10 mM TCA metabolite treatments in maize. a Root hair phenotype of 10 mM α-ketoglutarate, citrate, and succinate treatment in maize. Scale bar, 0.15 mm. **b** Normalized root hair density under different treatments. Average density was calculated by measuring white level intensity in representative regions using the line tool from Fiji. **c** Root hair length under different treatments. For the boxplots, the central line indicates the median; the bounds of the box show the 25th and 75th percentiles; and the whiskers indicate the maximum and minimum values. Data are presented as boxplots with each dot representing the datapoint of one biological replicate. Asterisks indicate statistical significance by one-way ANOVA (all *n* and *P* values are indicated in the graph). Source data are provided as a Source Data file.

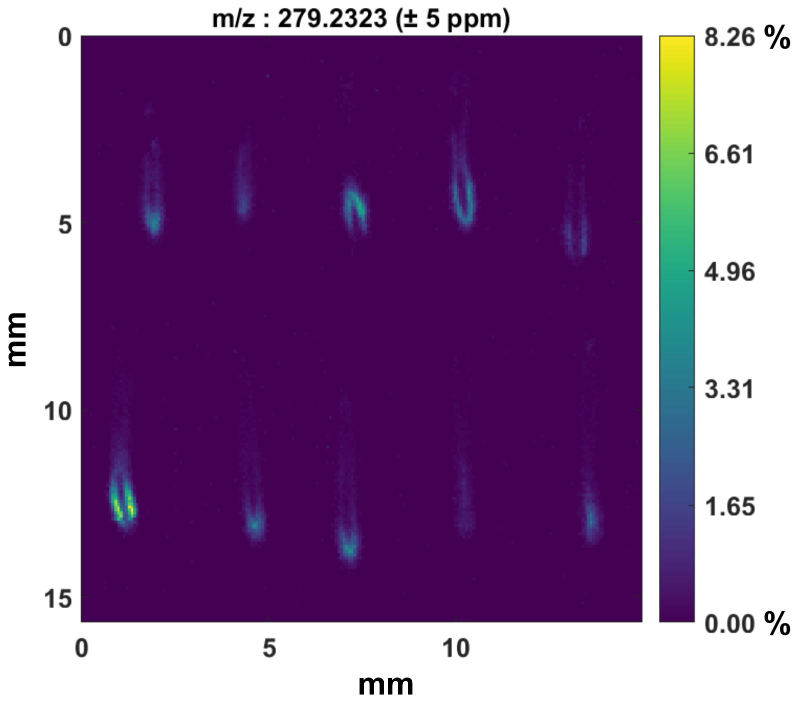

Supplementary Fig. 11 | DESI-MS images of fatty acid(18:2) (*m/z* 279.2323, [M−H]⁻) from all ten imaged B73 maize root sections (across 3 biological replicates). Images are normalized as a percentage of total ion current, and the maximum intensity is 8.263%.

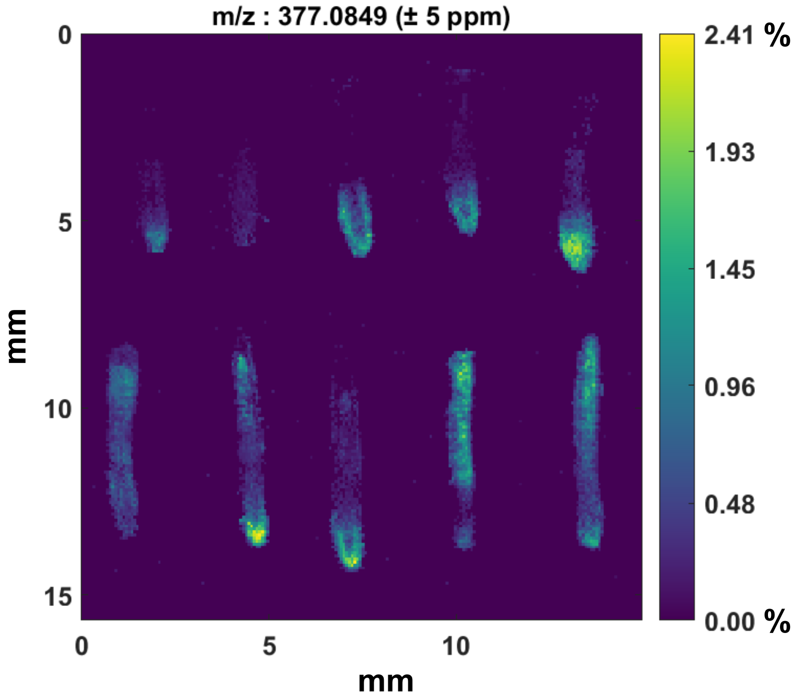

Supplementary Fig. 12 | DESI-MS images of hexose-hexose (*m/z* 377.0849, [M+Cl]⁻) from all ten imaged B73 maize root sections (across 3 biological replicates). Images are normalized as a percentage of total ion current, and the maximum intensity is 2.412%.

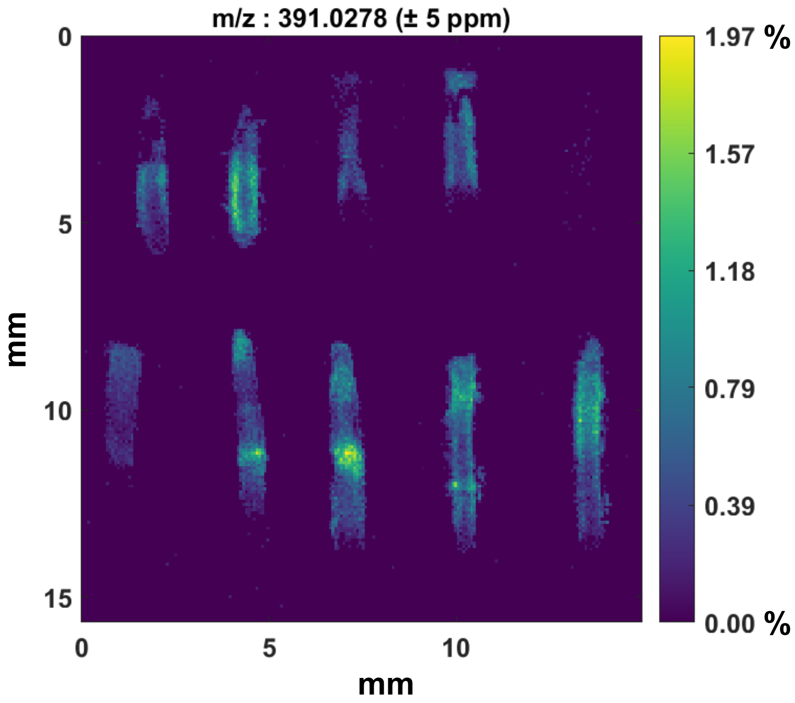

Supplementary Fig. 13 | DESI-MS images of unknown 1 (*m/z* 391.0278, negative ionization mode) from all ten imaged B73 maize root sections (across 3 biological replicates). Images are normalized as a percentage of total ion current, and the maximum intensity is 1.966%.

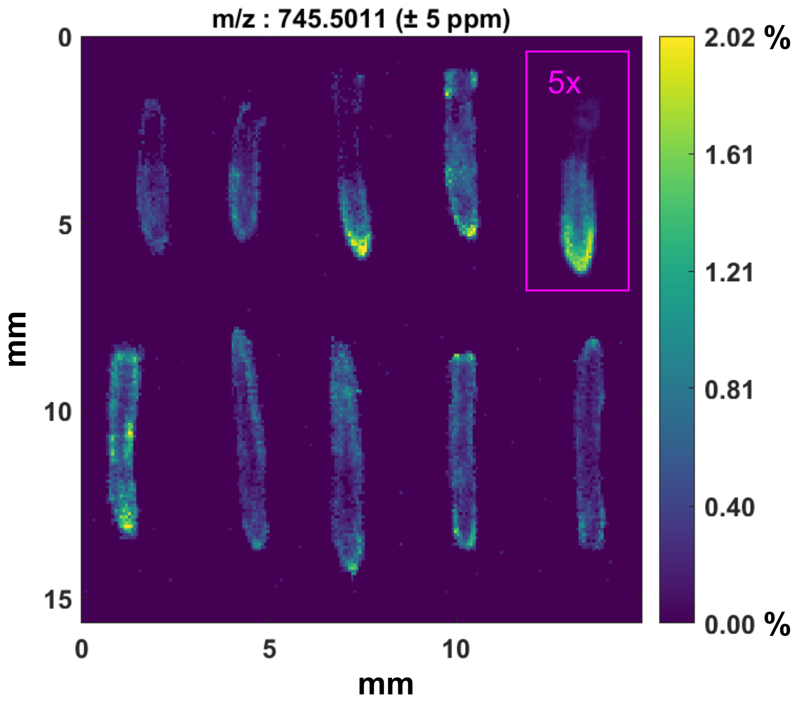

Supplementary Fig. 14 | DESI-MS images of phosphatidylglycerol (16:0/18:2) (*m/z* 745.5011, $[M-H]^-$) from all ten imaged B73 maize root sections (across 3 biological replicates). Images are normalized as a percentage of total ion current, and the maximum intensity is 2.016%, except for the root section outlined in pink. For that section only, the max intensity is 5x higher: 10.078%.

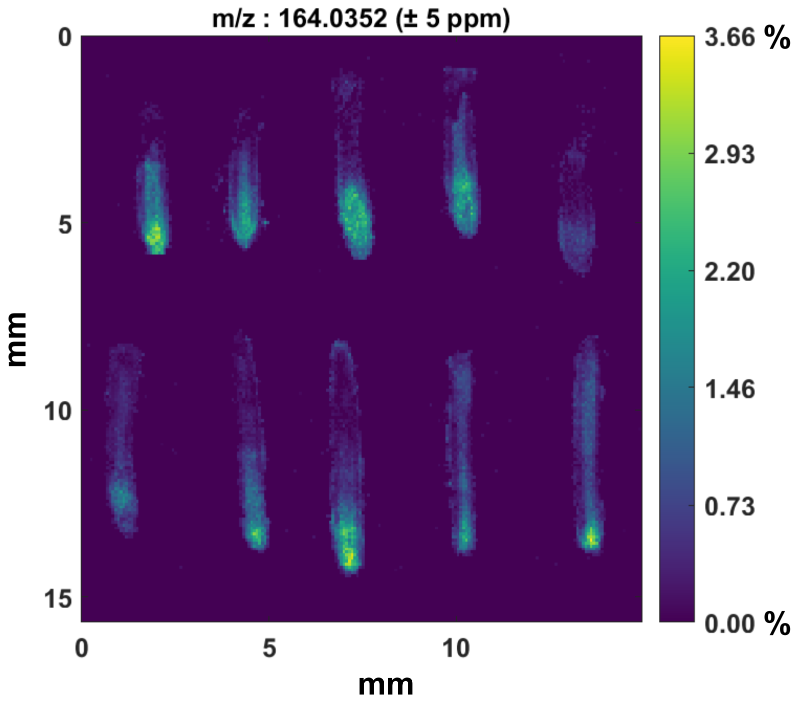

Supplementary Fig. 15 | DESI-MS images of MBOA (*m/z* 164.0352, [M−H]⁻) from all ten imaged B73 maize root sections (across 3 biological replicates). Images are normalized as a percentage of total ion current, and the maximum intensity is 3.662%.

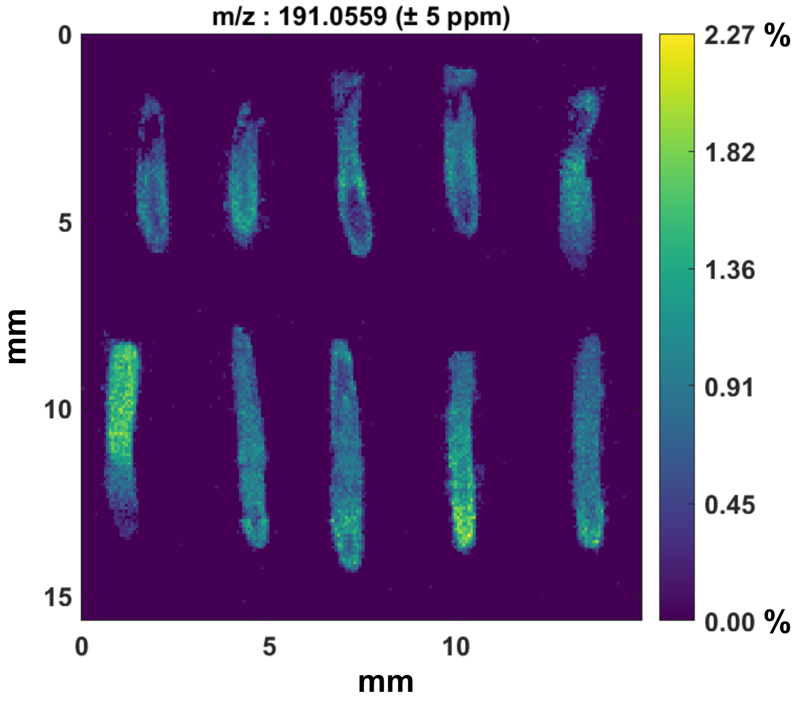

Supplementary Fig. 16 | DESI-MS images of quinate (*m/z* 191.0559, [M−H]⁻) from all ten imaged B73 maize root sections (across 3 biological replicates). Images are normalized normalized as a percentage of total ion current, and the maximum intensity is 2.27%.

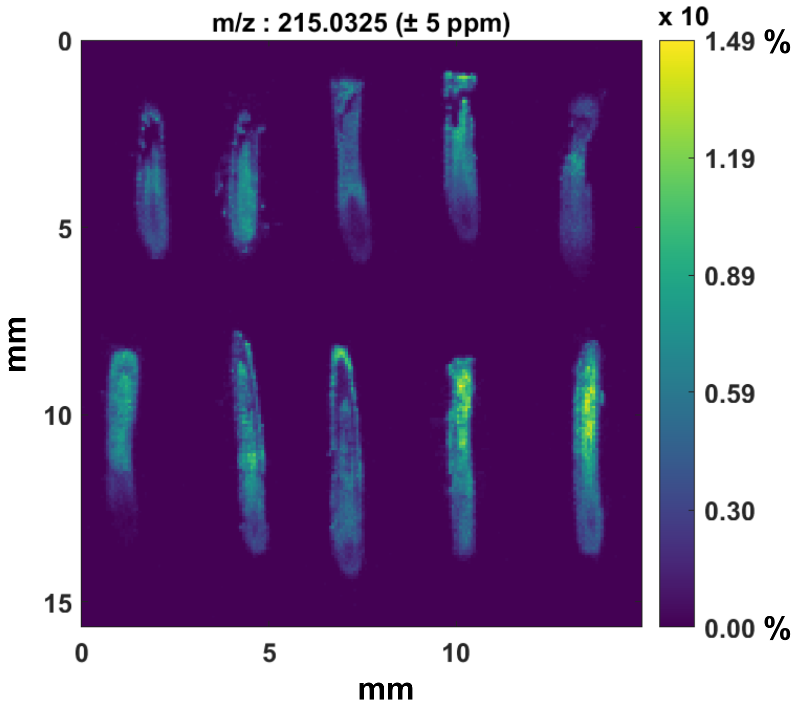

Supplementary Fig. 17 | DESI-MS images of hexose (*m/z* 215.0325, $[M+Cl]^-$) from all ten imaged B73 maize root sections (across 3 biological replicates). Images are normalized as a percentage of total ion current, and the maximum intensity is 14.853%.

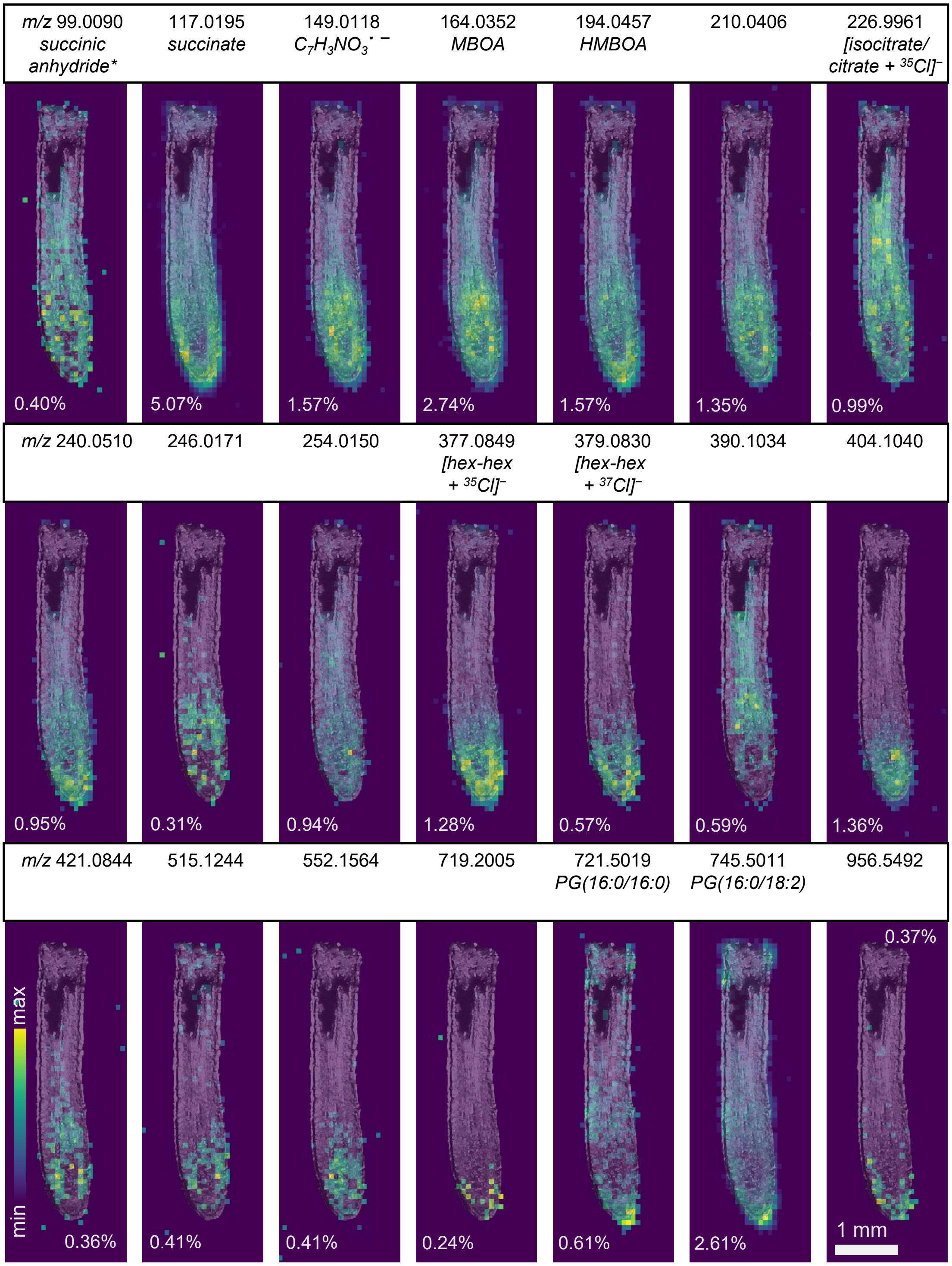

Supplementary Fig. 18 | DESI-MS images of tip- and stele-localized compounds with brightfield overlay. Some peaks have been identified, while others are unknown, highlighting the potential of this technique for discovery. The maximum peak intensity as a percentage of TIC is shown for each image. Identifications indicated with an asterisk are possible matches, unconfirmed by MS/MS. All other named compounds have MS/MS patterns included in the Supplementary Information. All images were obtained in the negative ionization mode, and named ions are deprotonated unless otherwise indicated. This experiment was replicated ten times across three biological samples (Supplementary Table 1). Data from one representative tissue section are shown here.

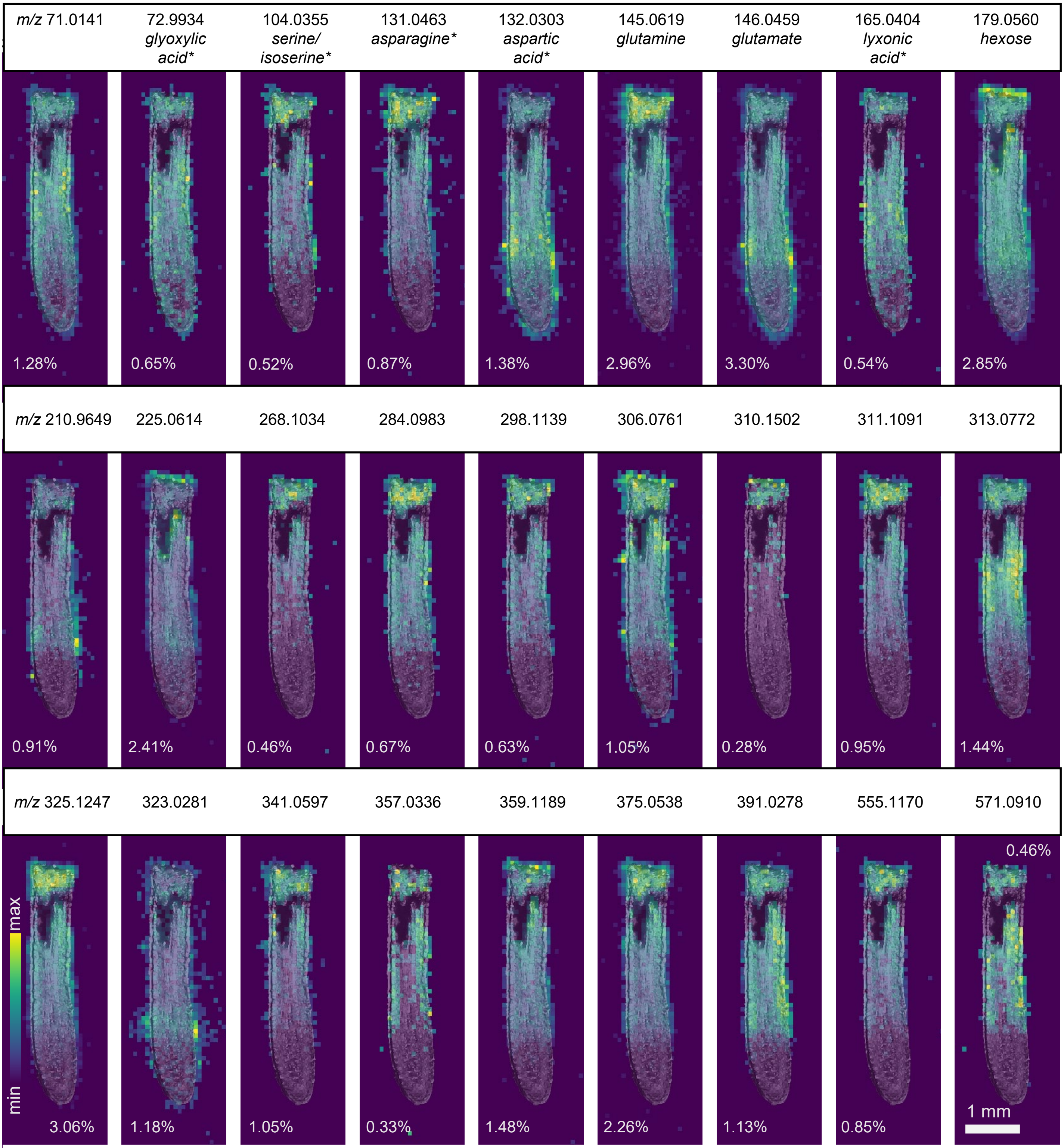

Supplementary Fig. 19 | DESI-MS images of additional compounds enriched in the elongation and differentiation zones with brightfield overlay. Some peaks have been identified, while others are unknown, highlighting the potential of this technique for discovery. The maximum peak intensity as a percentage of TIC is shown for each image. Identifications indicated with an asterisk are possible matches, unconfirmed by MS/MS. All other named compounds have MS/MS patterns included in the Supplementary Information. All images were obtained in the negative ionization mode, and named ions are deprotonated unless otherwise indicated. This experiment was replicated ten times across three biological samples (Supplementary Table 1). Data from one representative tissue section are shown here.

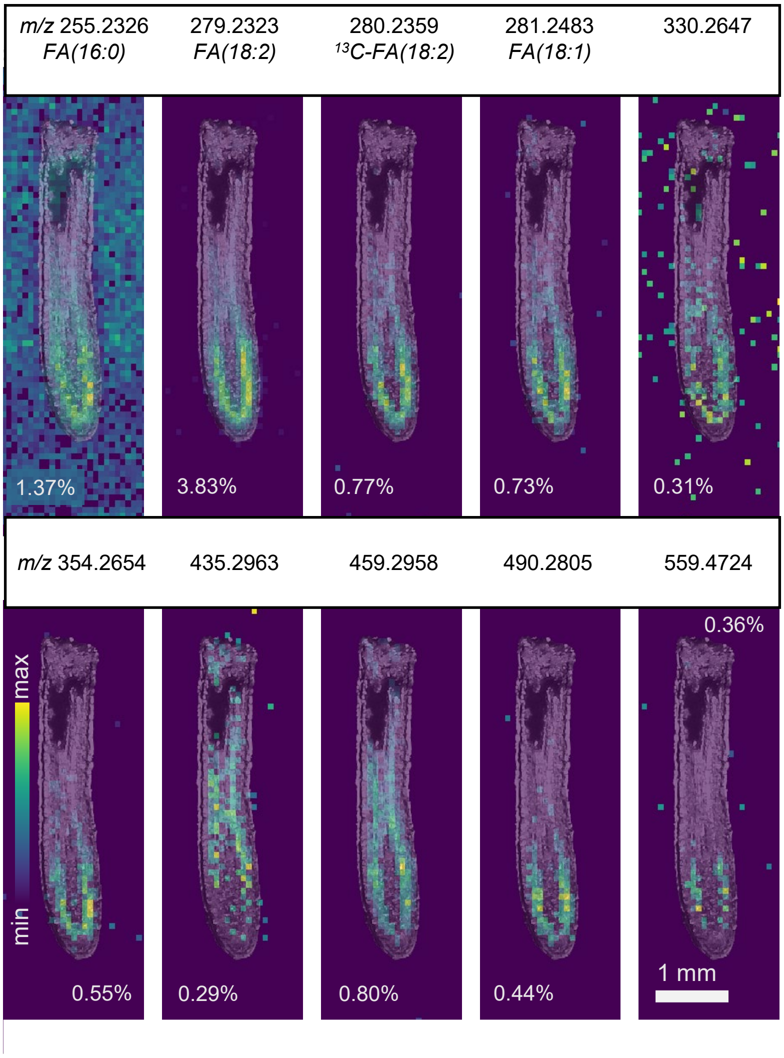

Supplementary Fig. 20 | DESI-MS images of compounds enriched in the endodermis/cortex with brightfield overlay. Some peaks have been identified, while others are unknown, highlighting the potential of this technique for discovery. The maximum peak intensity as a percentage of TIC is shown for each image. MS/MS patterns are included in the Supplementary Information for named peaks. All images were obtained in the negative ionization mode, and named ions are deprotonated. This experiment was replicated ten times across three biological samples (Supplementary Table 1). Data from one representative tissue section are shown here.

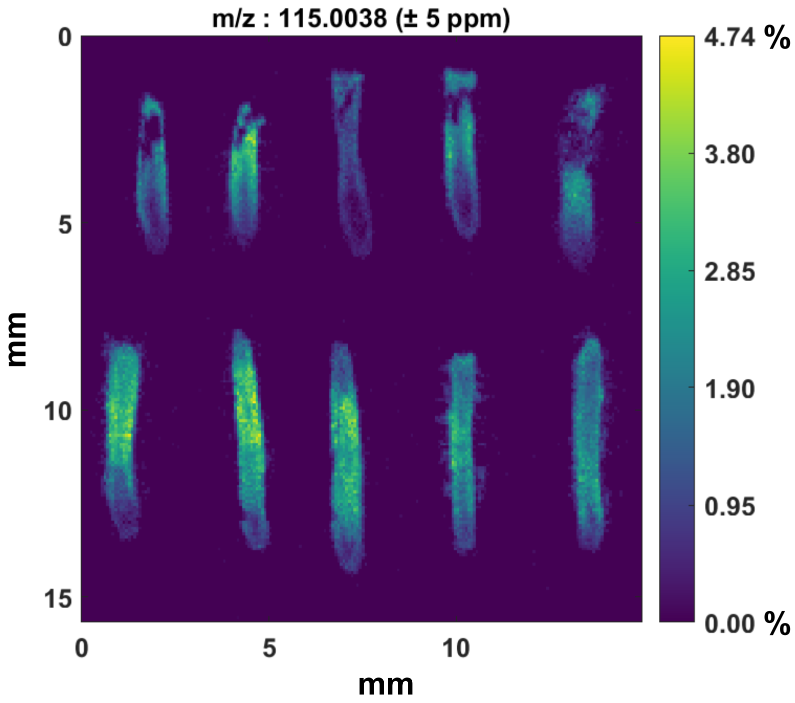

Supplementary Fig. 21 | DESI-MS images of fumarate (*m/z* 115.0038, [M−H]⁻) from all ten imaged B73 maize root sections (across 3 biological replicates). Images are normalized as a percentage of total ion current, and the maximum intensity is 4.744%.

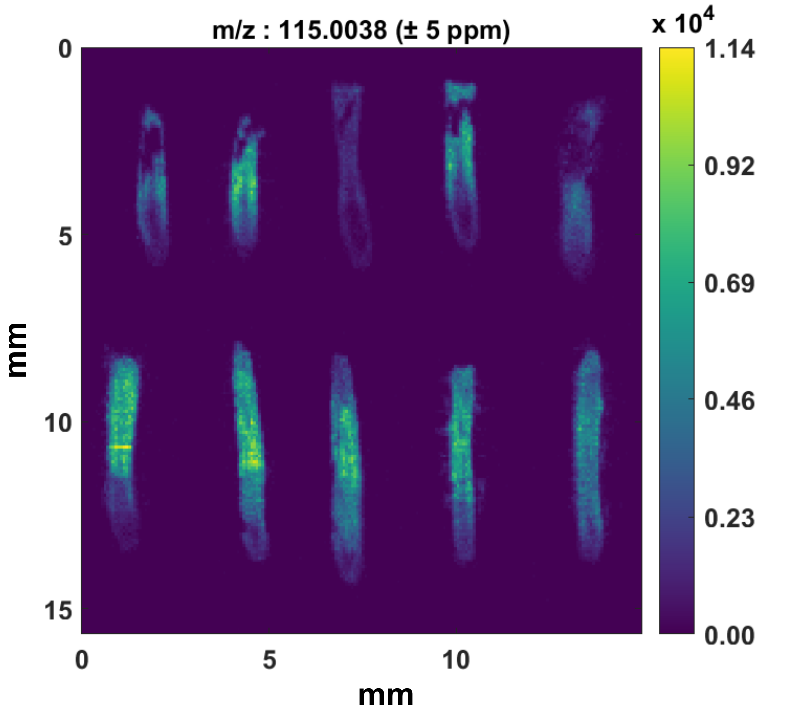

Supplementary Fig. 22 | DESI-MS images of fumarate (*m/z* 115.0038, [M−H]⁻) from all ten imaged B73 maize root sections (across 3 biological replicates). Images are unnormalized, and the maximum intensity is 11448.

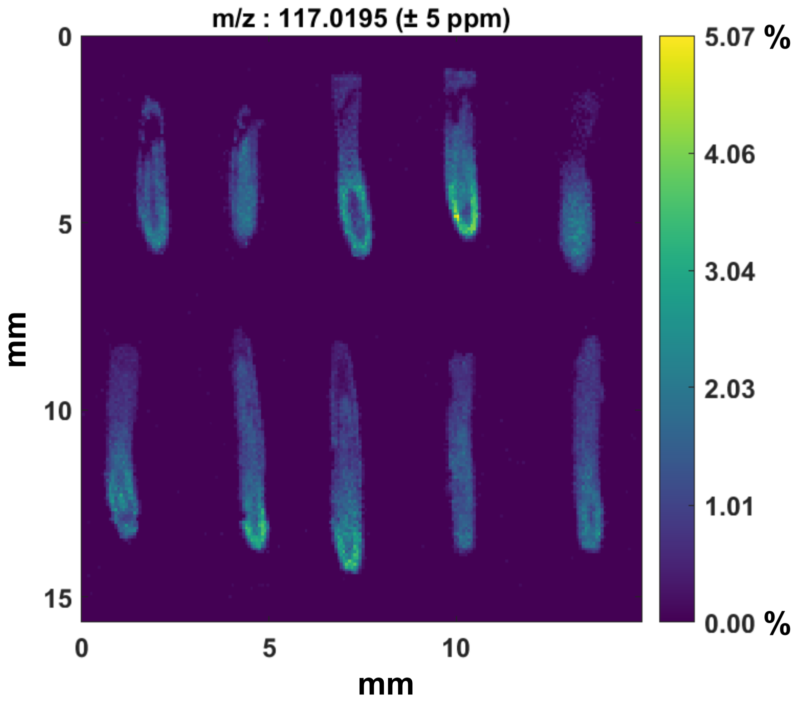

Supplementary Fig. 23 | DESI-MS images of succinate (*m/z* 117.0195, $[M-H]^-$) from all ten imaged B73 maize root sections (across 3 biological replicates). Images are normalized as a percentage of total ion current, and the maximum intensity is 5.07%.

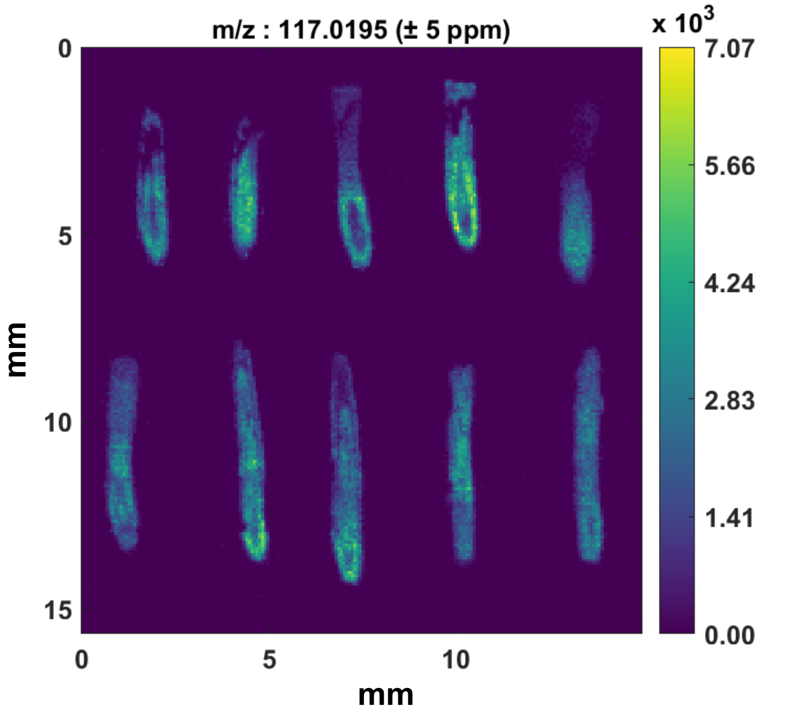

Supplementary Fig. 24 | DESI-MS images of succinate (*m/z* 117.0195, $[M-H]^-$) from all ten imaged B73 maize root sections (across 3 biological replicates). Images are unnormalized, and the maximum intensity is 7070.

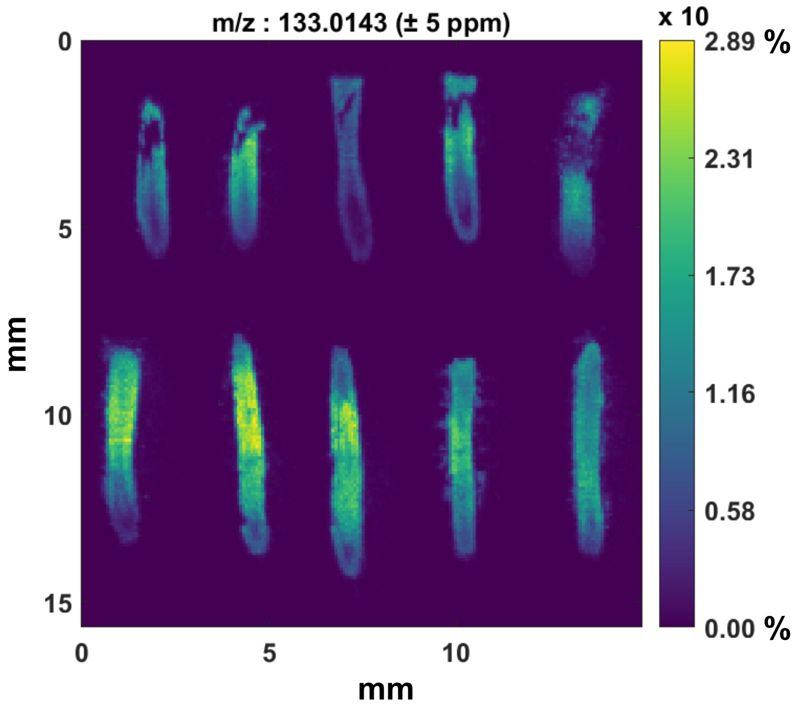

Supplementary Fig. 25 | DESI-MS images of malate (*m/z* 133.0143, [M−H]⁻) from all ten imaged B73 maize root sections (across 3 biological replicates). Images are normalized as a percentage of total ion current, and the maximum intensity is 28.897%.

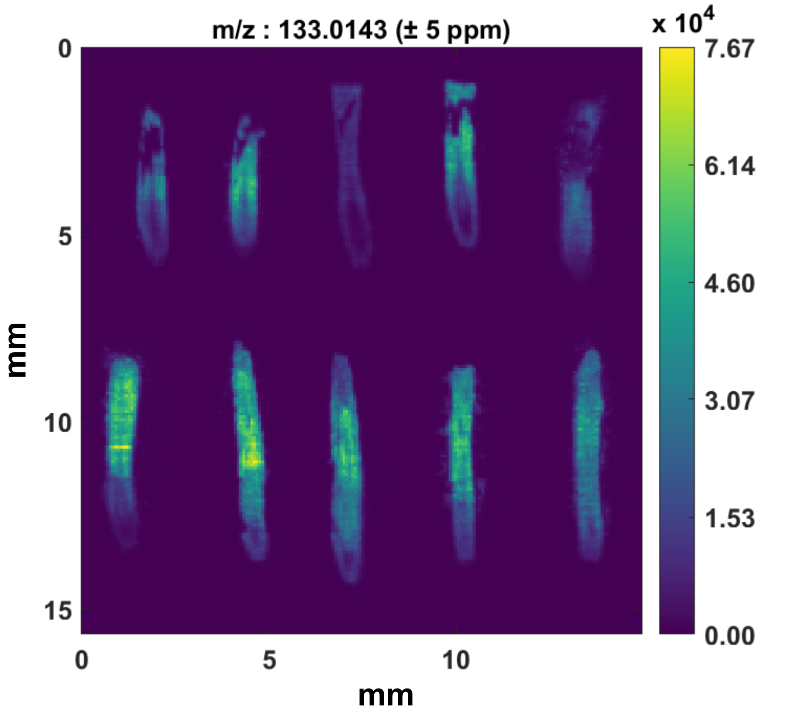

Supplementary Fig. 26 | DESI-MS images of malate (*m/z* 133.0143, [M−H]⁻) from all ten imaged B73 maize root sections (across 3 biological replicates). Images are unnormalized, and the maximum intensity is 76746.

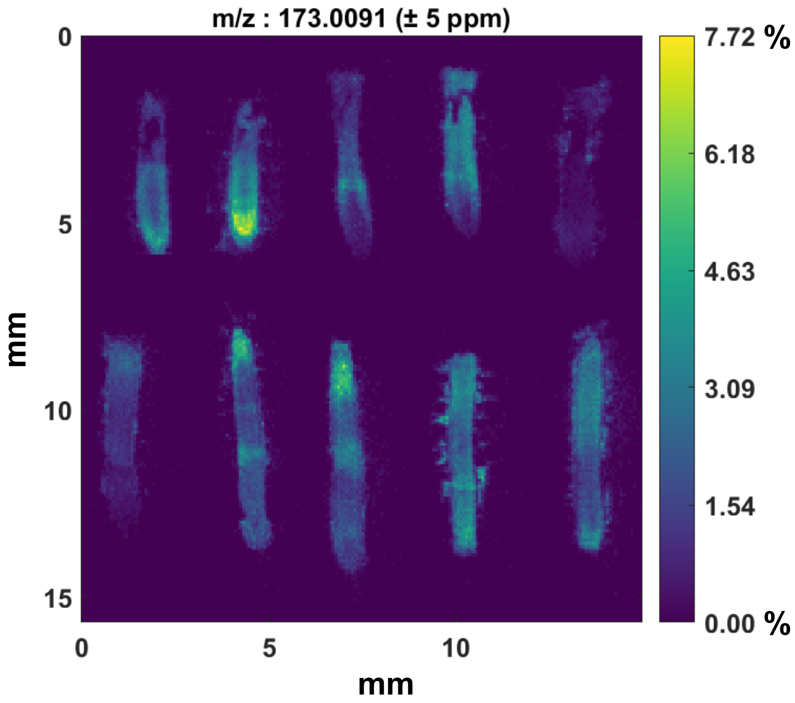

Supplementary Fig. 27 | DESI-MS images of aconitate (*m/z* 173.0091, $[M-H]^-$) from all ten imaged B73 maize root sections (across 3 biological replicates). Images are normalized as a percentage of total ion current, and the maximum intensity is 7.722%.

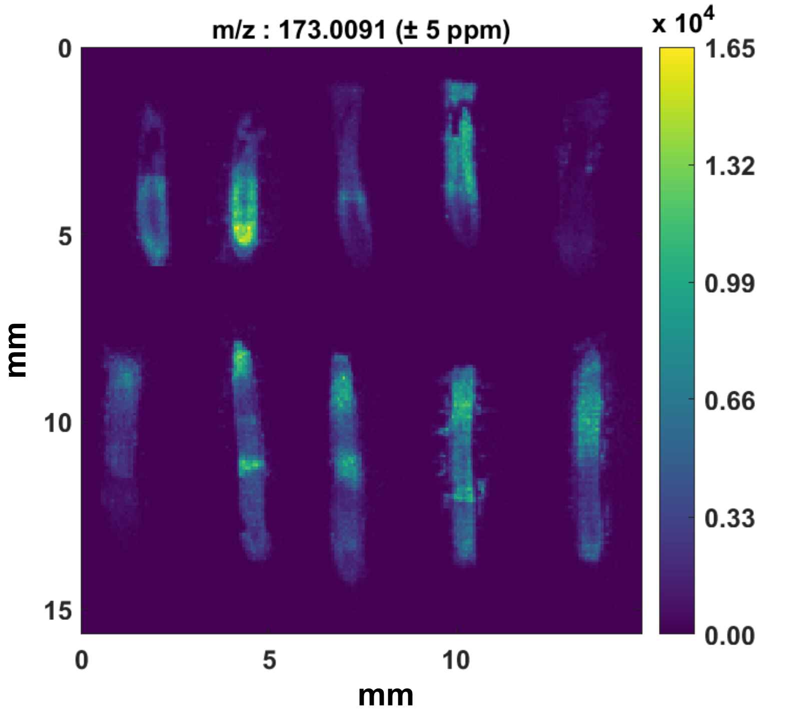

Supplementary Fig. 28 | DESI-MS images of aconitate (*m/z* 173.0091, $[M-H]^-$) from all ten imaged B73 maize root sections (across 3 biological replicates). Images are unnormalized, and the maximum intensity is 16478.

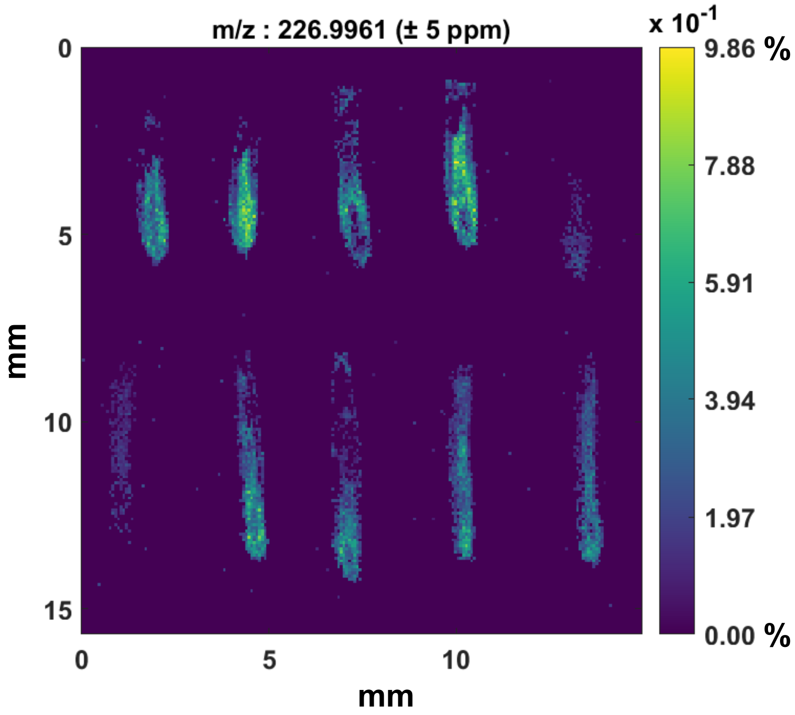

Supplementary Fig. 29 | DESI-MS images of citrate/isocitrate (*m/z* 226.9961, $[M+Cl]^-$) from all ten imaged B73 maize root sections (across 3 biological replicates). Images are normalized as a percentage of total ion current, and the maximum intensity is 0.986%.

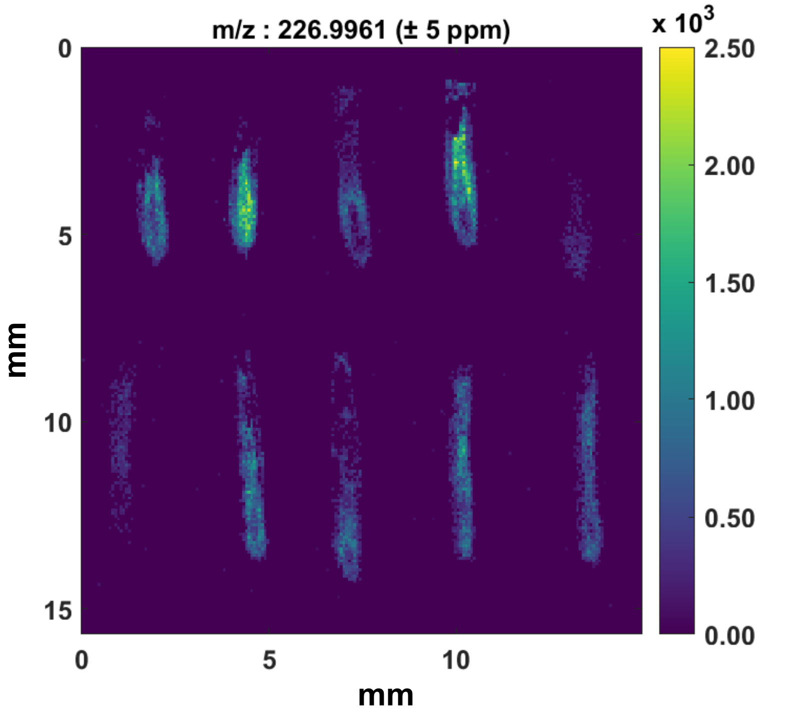

Supplementary Fig. 30 | DESI-MS images of citrate/isocitrate (*m/z* 226.9961, $[M+Cl]^-$) from all ten imaged B73 maize root sections (across 3 biological replicates). Images are unnormalized, and the maximum intensity is 2499.

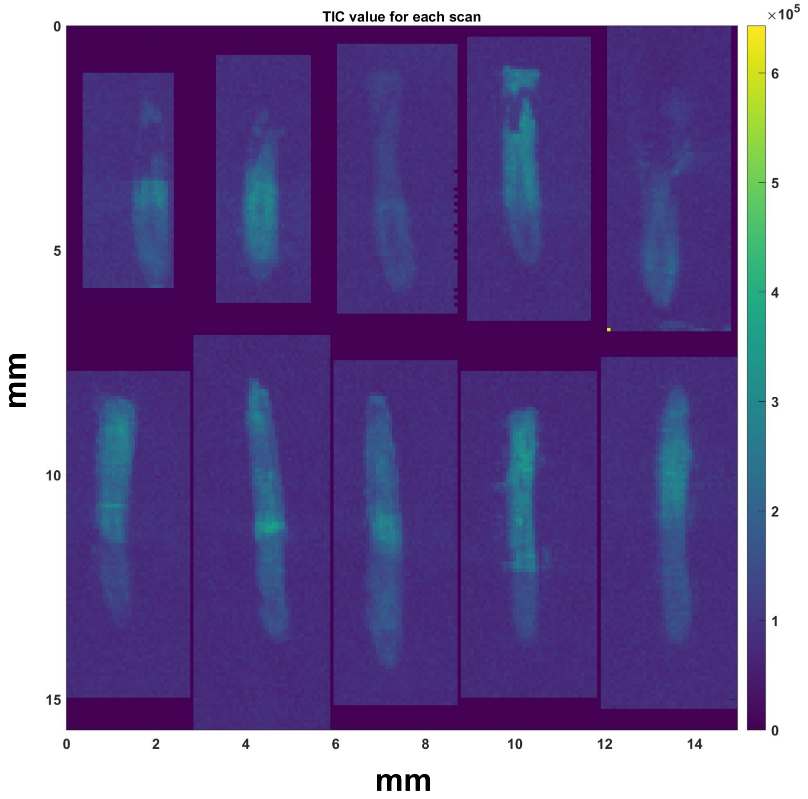

Supplementary Fig. 31 | MS images of the total ion current (TIC) by pixel for each of the ten imaged B73 maize root sections in the negative ionization mode. The maximum intensity is 643069.

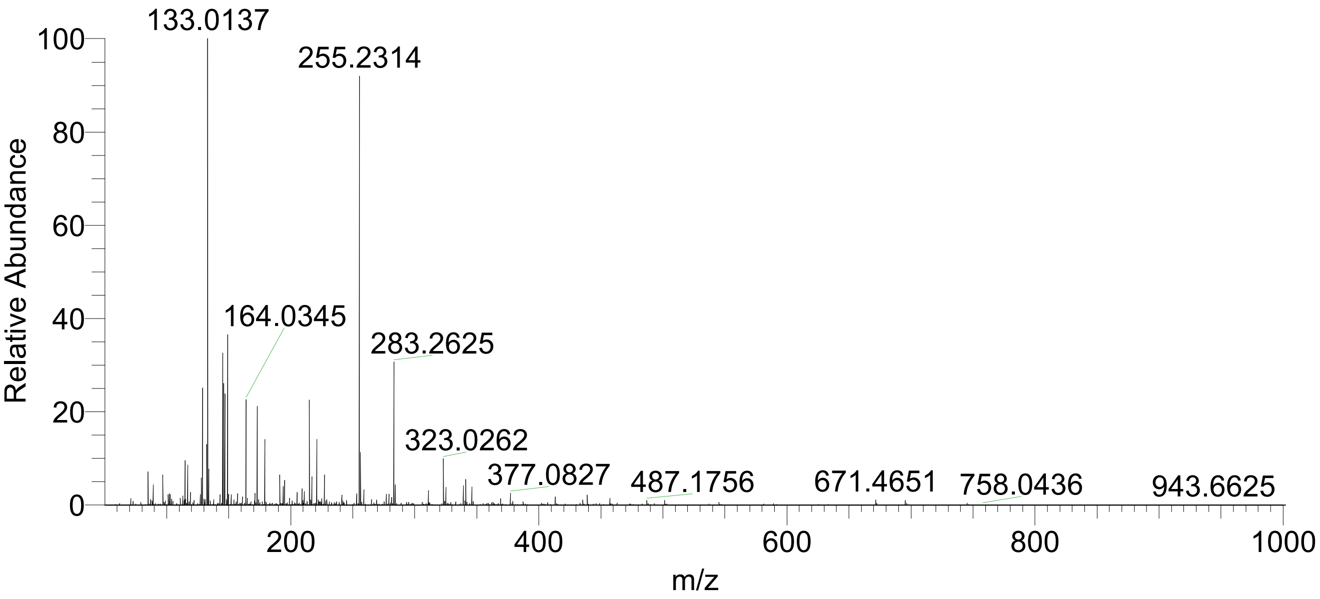

Supplementary Fig. 32 | Full negative-mode mass spectrum of B73 maize root extract.

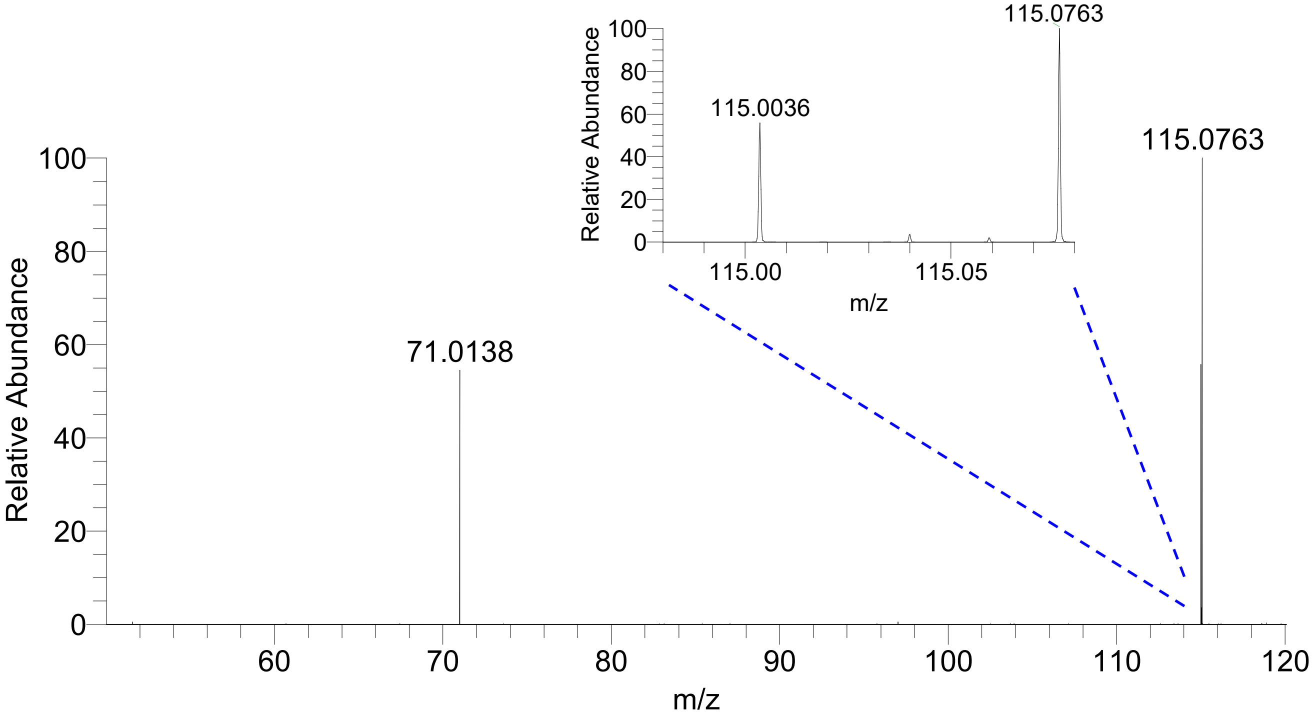

Supplementary Fig. 33 | MS/MS spectrum of fumarate (*m/z* 115.0036, [M−H]⁻) with NCE 20%. Inset zooms in on isolated parent peak window to show fumarate.

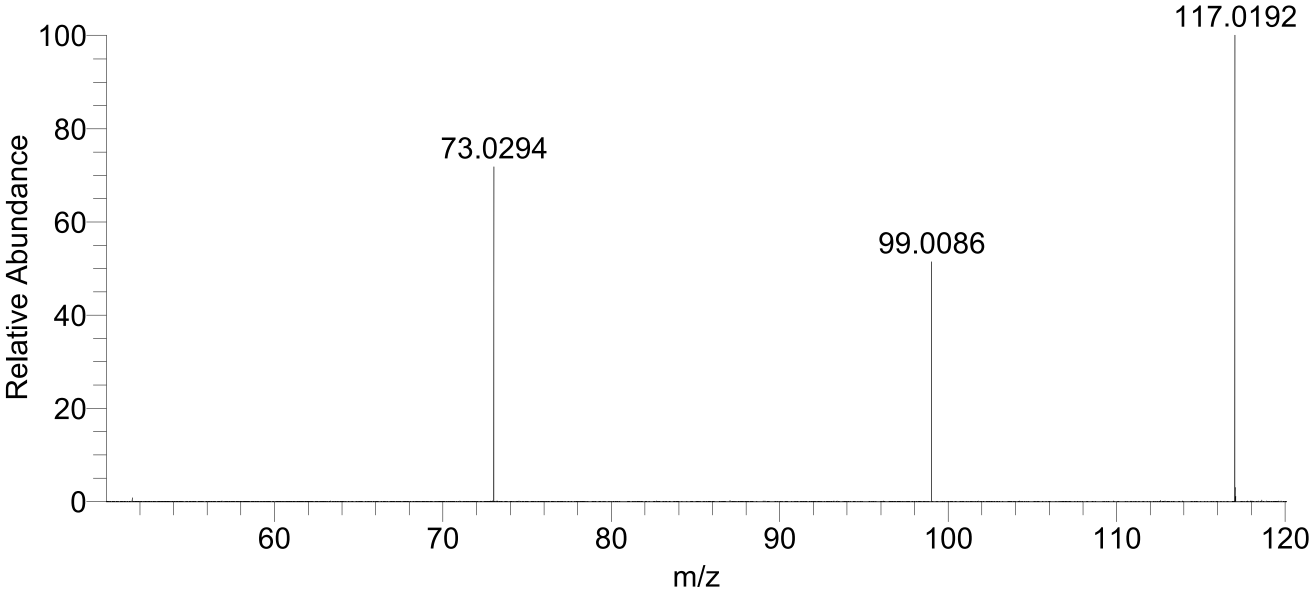

Supplementary Fig. 34 | MS/MS spectrum of succinate (*m/z* 117.0192, [M−H]⁻) with NCE 20%.

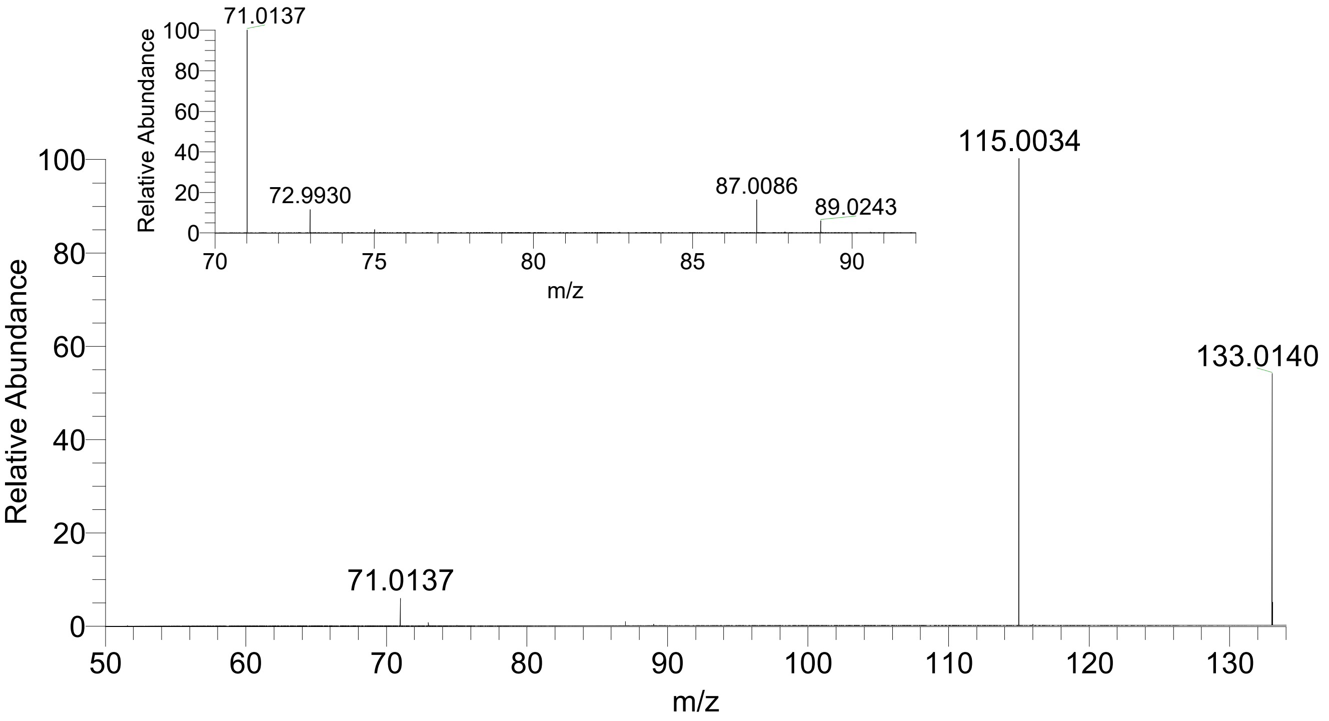

Supplementary Fig. 35 | MS/MS spectrum of malate (*m/z* 133.0140, [M−H]⁻) with NCE 20%. Inset expands *m/z* 70-92 to help visualize low abundance fragments.

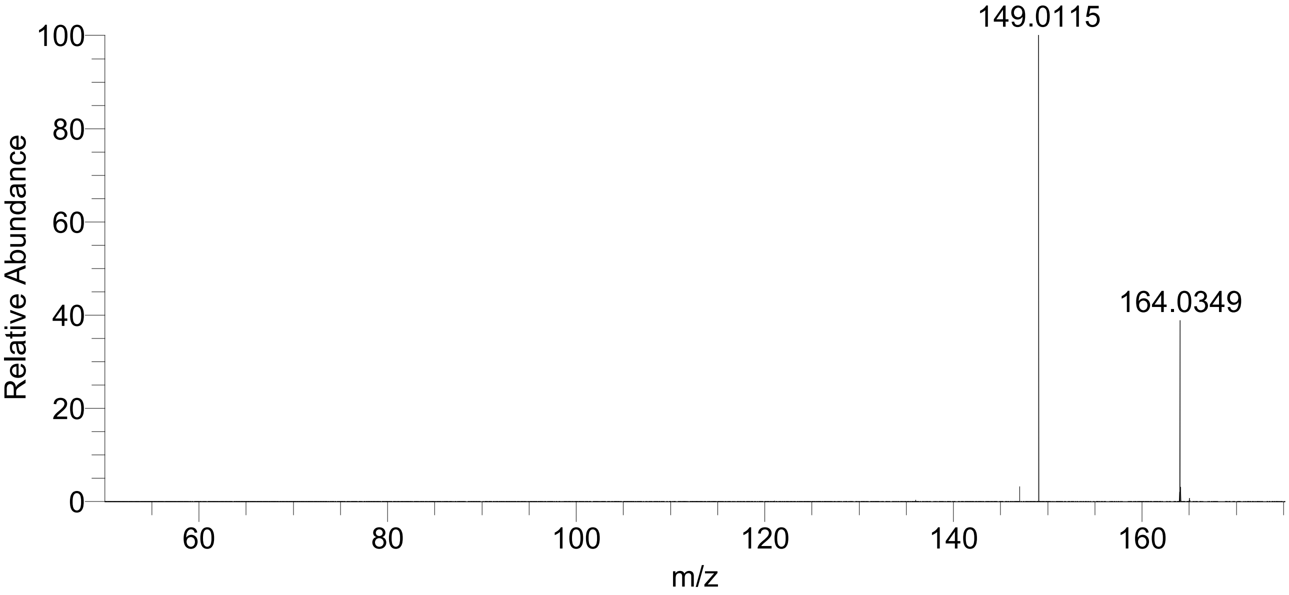

Supplementary Fig. 36 | MS/MS spectrum of MBOA (*m/z* 164.0349, [M−H]⁻) with NCE 25%.

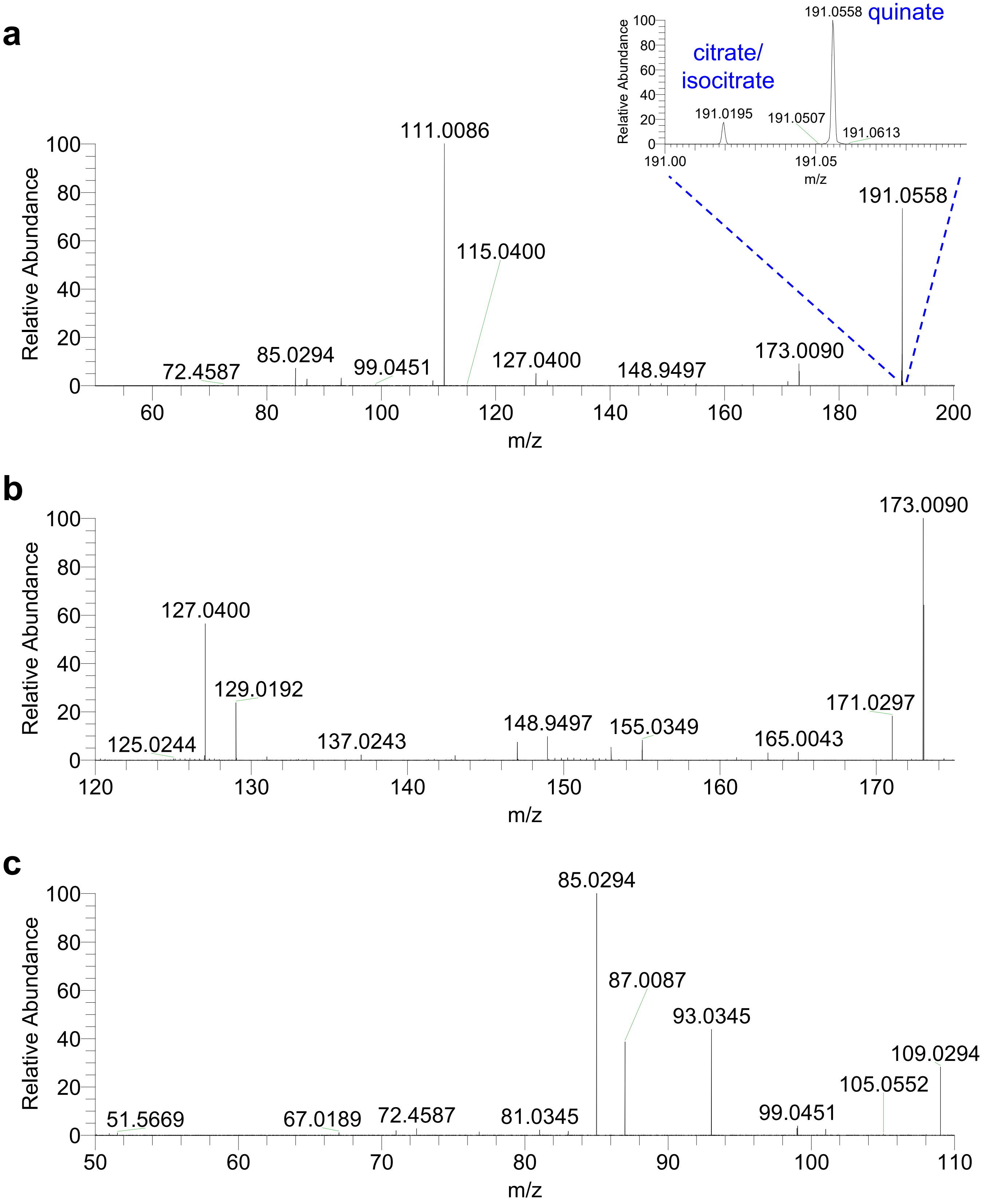

Supplementary Fig. 37 | a, MS/MS spectrum of quinate (*m/z* 191.0558, [M−H]⁻) and citrate/isocitrate (*m/z* 191.0195, [M−H]⁻) with NCE 25%. Inset expands to show both parent peaks, which could not be individually isolated. Zoom in on *m/z* 120-175 (**b**) and *m/z* 50-110 (**c**) helps to better visualize low abundance fragments.

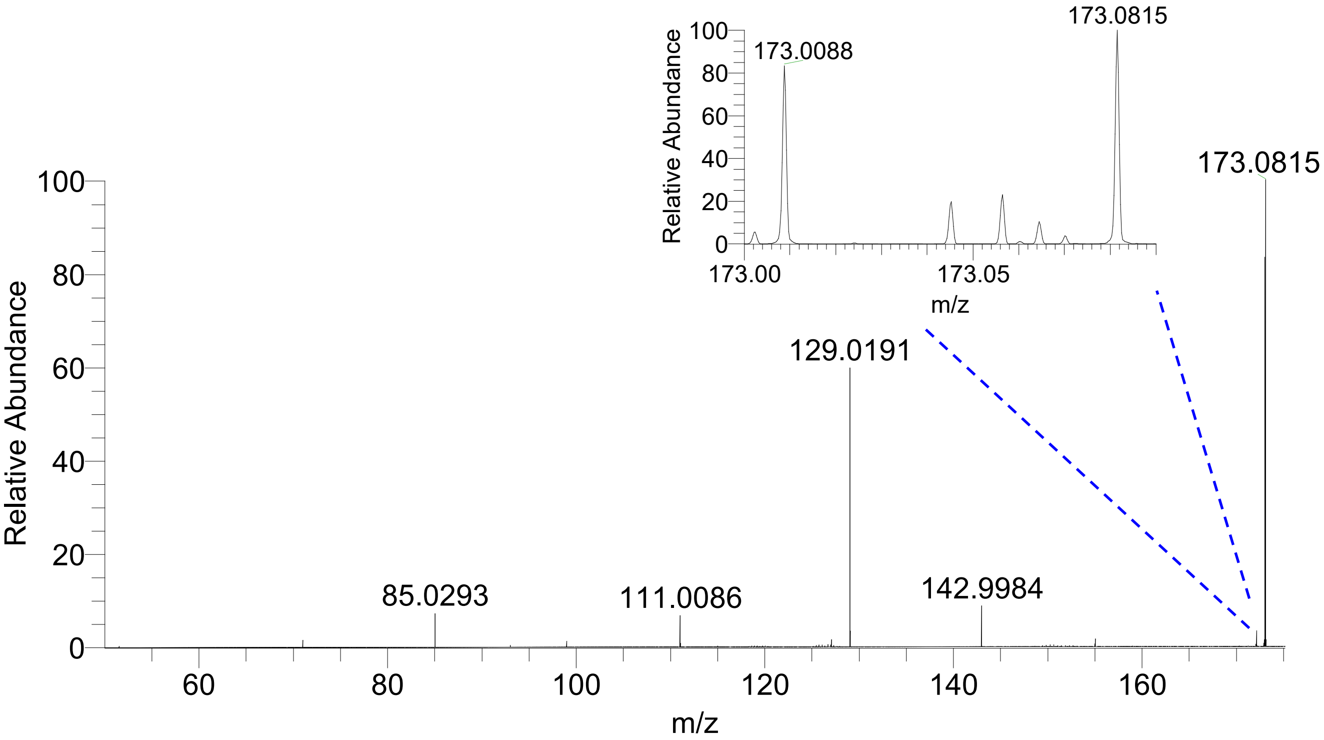

Supplementary Fig. 38 | MS/MS spectrum of aconitate (*m/z* 173.0088, [M−H]⁻) with NCE 15%. The isolation window also captured *m/z* 173.0815, an unknown, as shown by the inset.

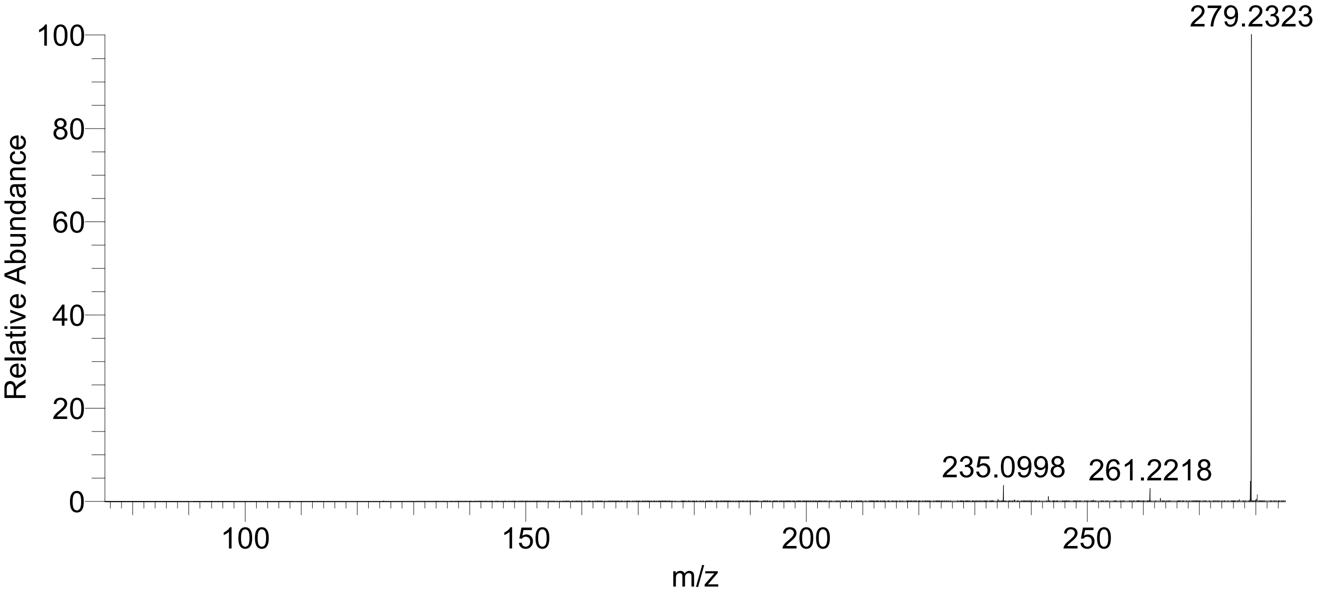

Supplementary Fig. 39 | MS/MS spectrum of FA(18:2) (*m/z* 279.2323, [M−H]⁻) with NCE 30%.

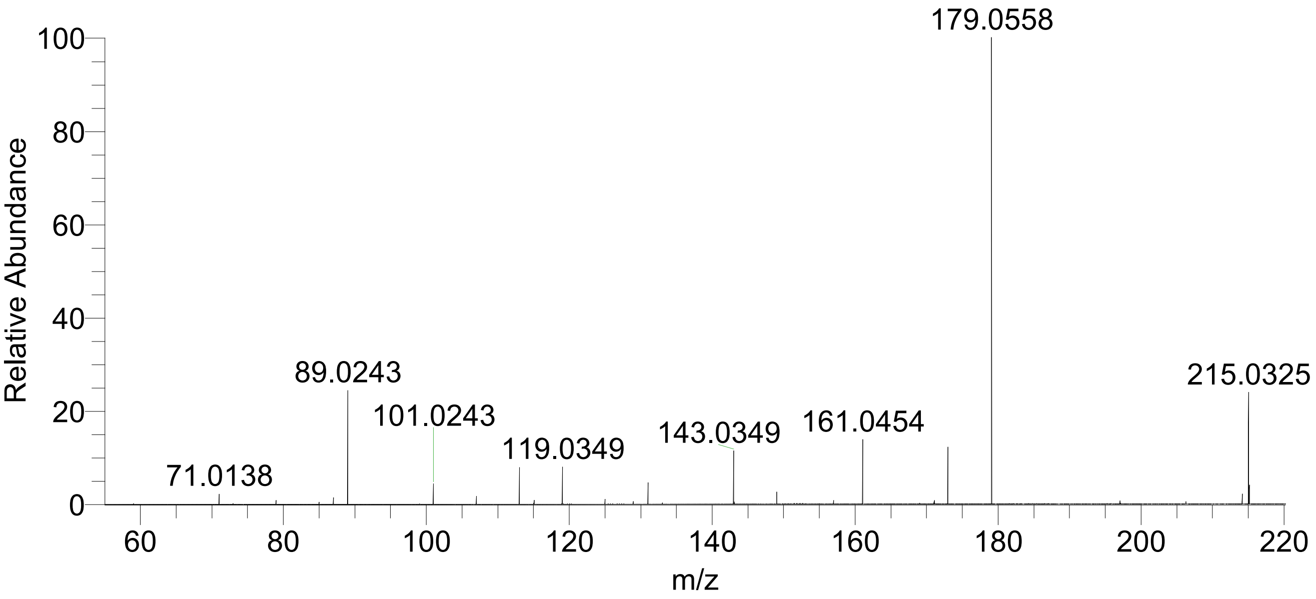

Supplementary Fig. 40 | MS/MS spectrum of hexose (*m/z* 215.0325) with NCE 20%. *m/z* 215.0325 represents the [M+Cl]⁻ peak, and *m/z* 179.0558 represents the [M−H]⁻ peak. The exact monosaccharide isomer cannot be definitively identified.

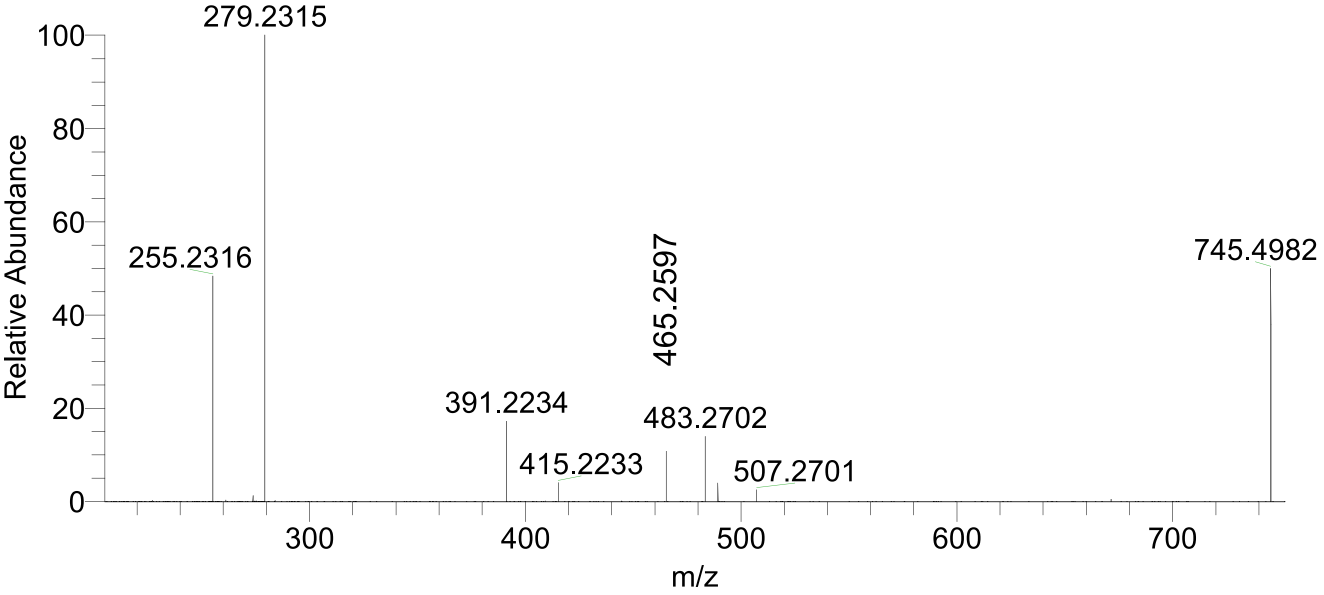

Supplementary Fig. 41 | MS/MS spectrum of PG(16:0/18:2) (*m/z* 745.4982, [M−H]⁻) with NCE 30%.

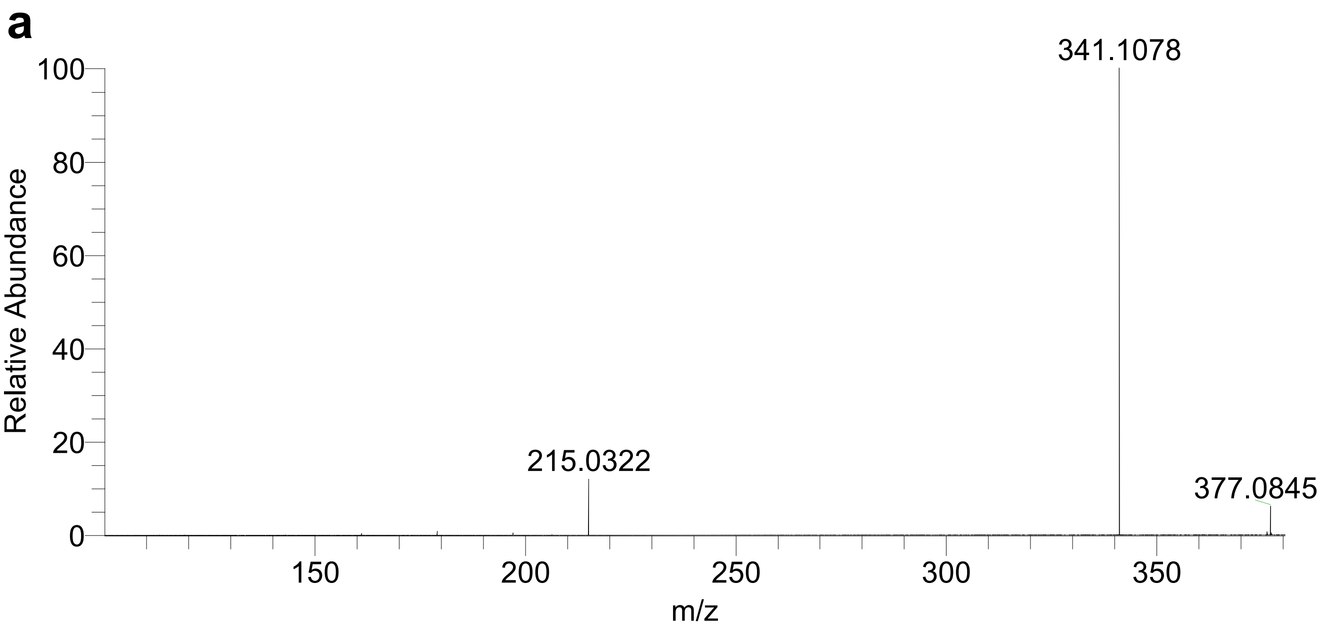

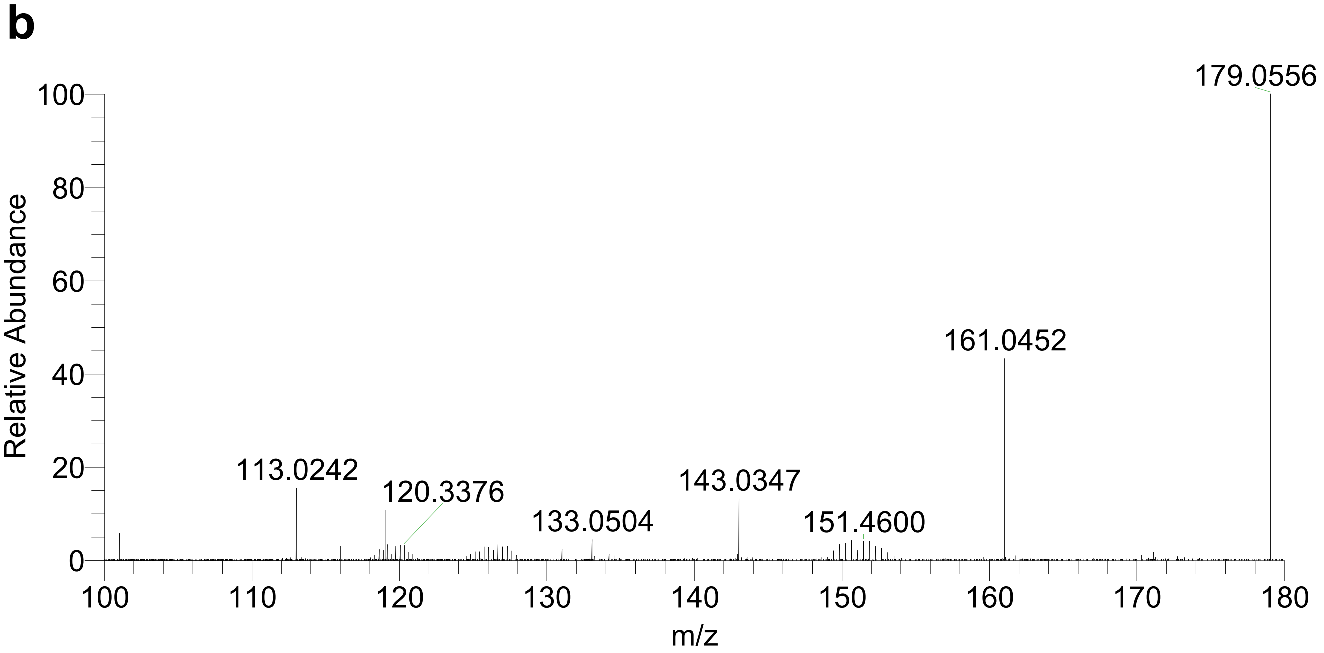

Supplementary Fig. 42 | a, MS/MS spectrum of hexose-hexose (*m/z* 377.0845) with NCE 22%. *m/z* 377.0845 represents the [M+Cl]⁻ peak, and *m/z* 341.1078 represents the [M−H]⁻ peak. **b,** Zoom in on *m/z* 100-180 better visualizes the low abundance peaks. The specific disaccharide isomer cannot be definitively identified.

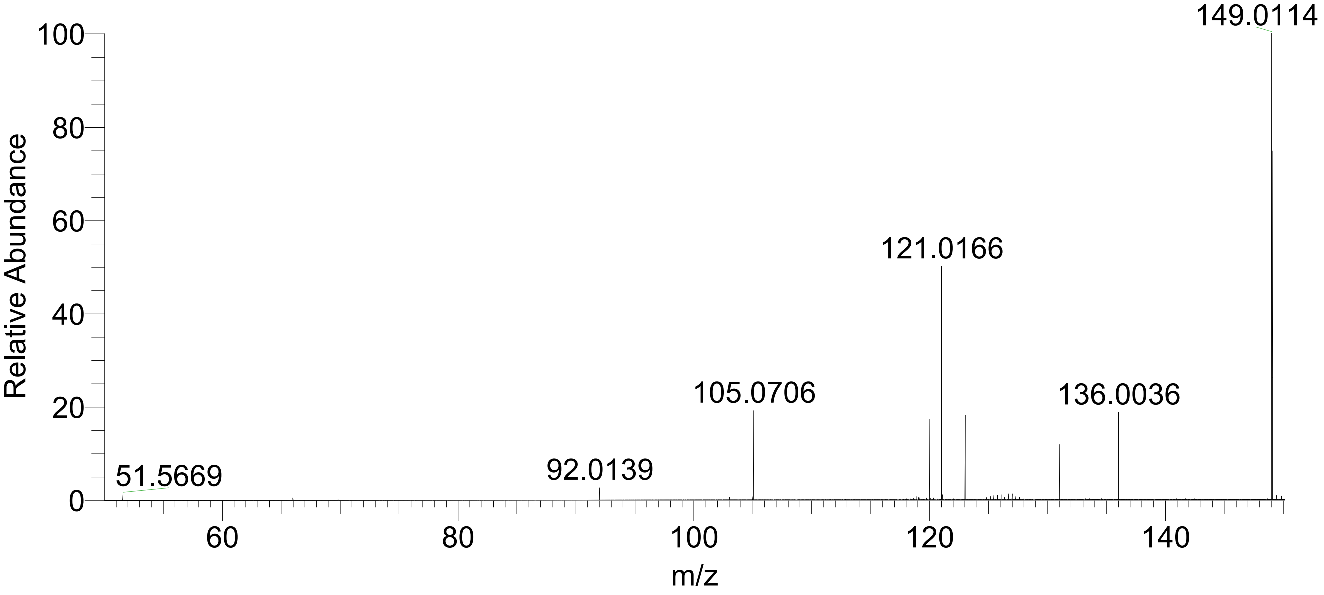

Supplementary Fig. 43 | MS/MS spectrum of $C_7H_3NO_3^{\cdot -}$, a benzoxazolinone fragment, (*m/z* 149.0114) with NCE 25%. Expected fragment is *m/z* 121.0166.

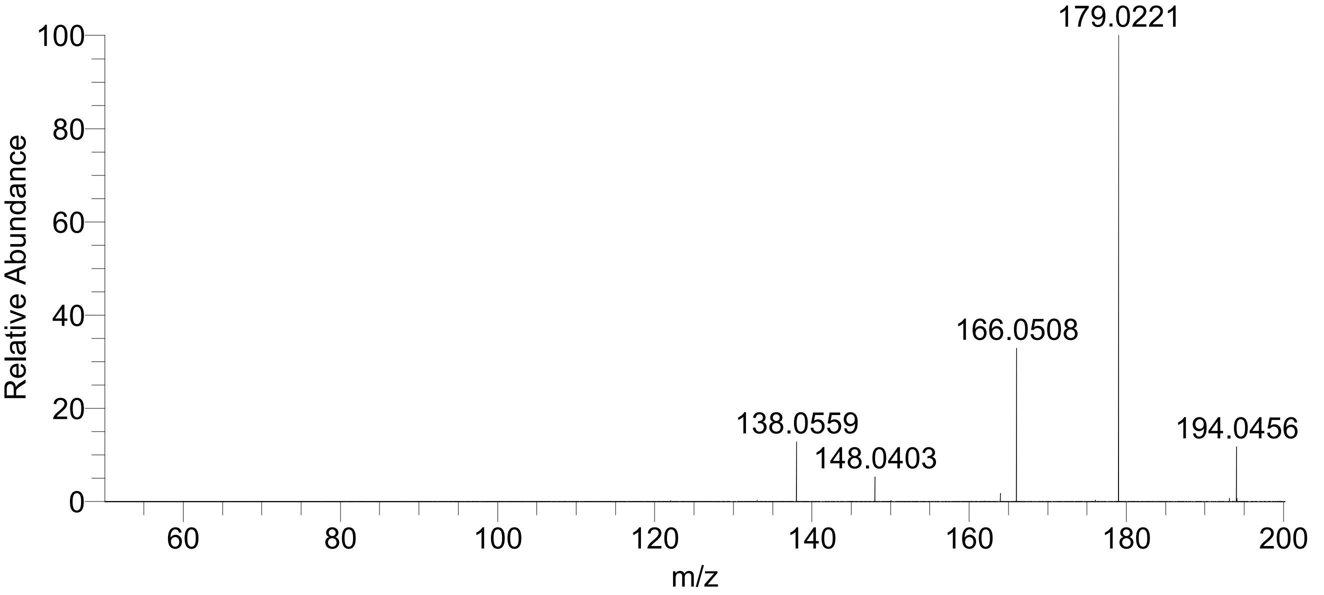

Supplementary Fig. 44 | MS/MS spectrum of HMBOA, 2-hydroxy-7-methoxy-1,4-benzoxazin-3-one, (*m/z* 194.0456, $[M-H]^-$) with NCE 25%.

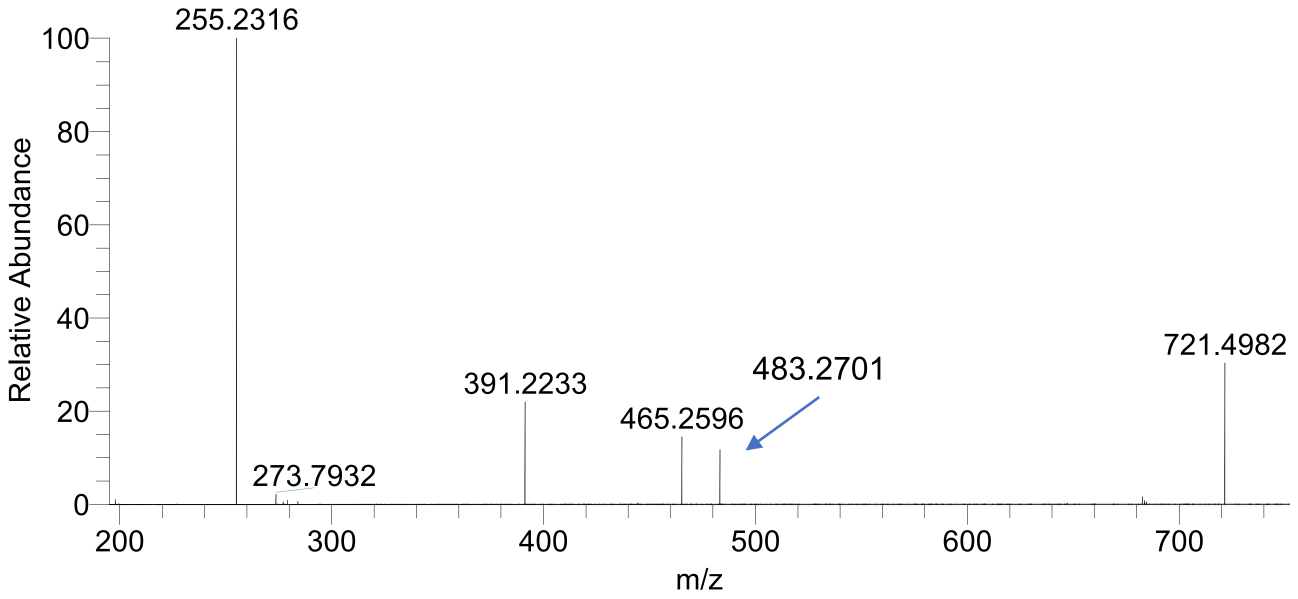

Supplementary Fig. 45 | MS/MS spectrum of PG(16:0/16:0) (*m/z* 721.4982, [M−H]$^-$) with NCE 28%.

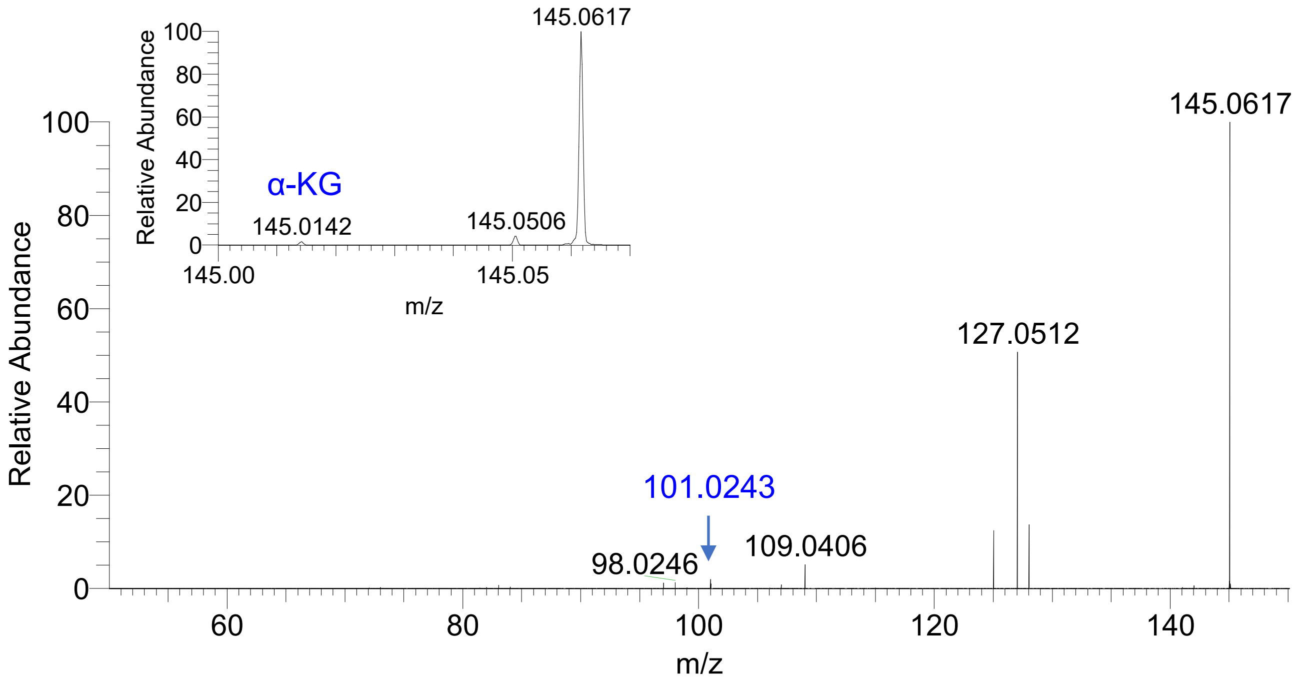

Supplementary Fig. 46 | MS/MS spectrum of glutamine (*m/z* 145.0617, [M−H]$^-$) with NCE 20%. In addition to glutamine, a small peak from α-ketoglutarate also co-isolated (*m/z* 145.0142, [M−H]$^-$) and produced the fragment at 101.0243.

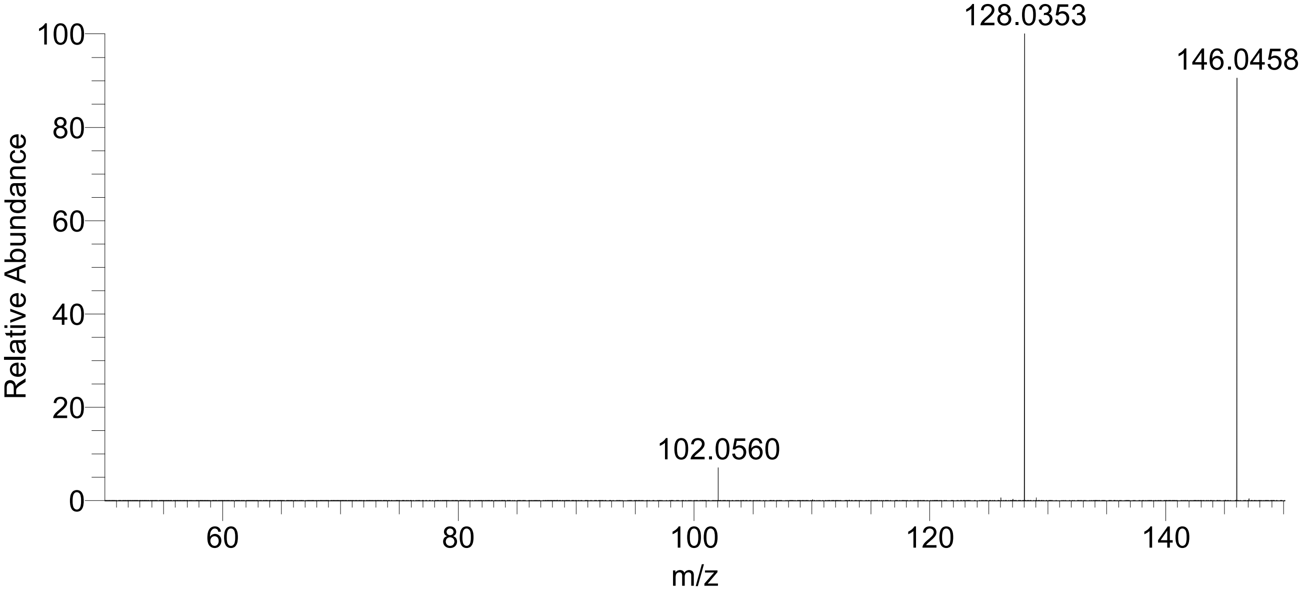

Supplementary Fig. 47 | MS/MS spectrum of glutamate (*m/z* 146.0458, [M−H]⁻) with NCE 20%.

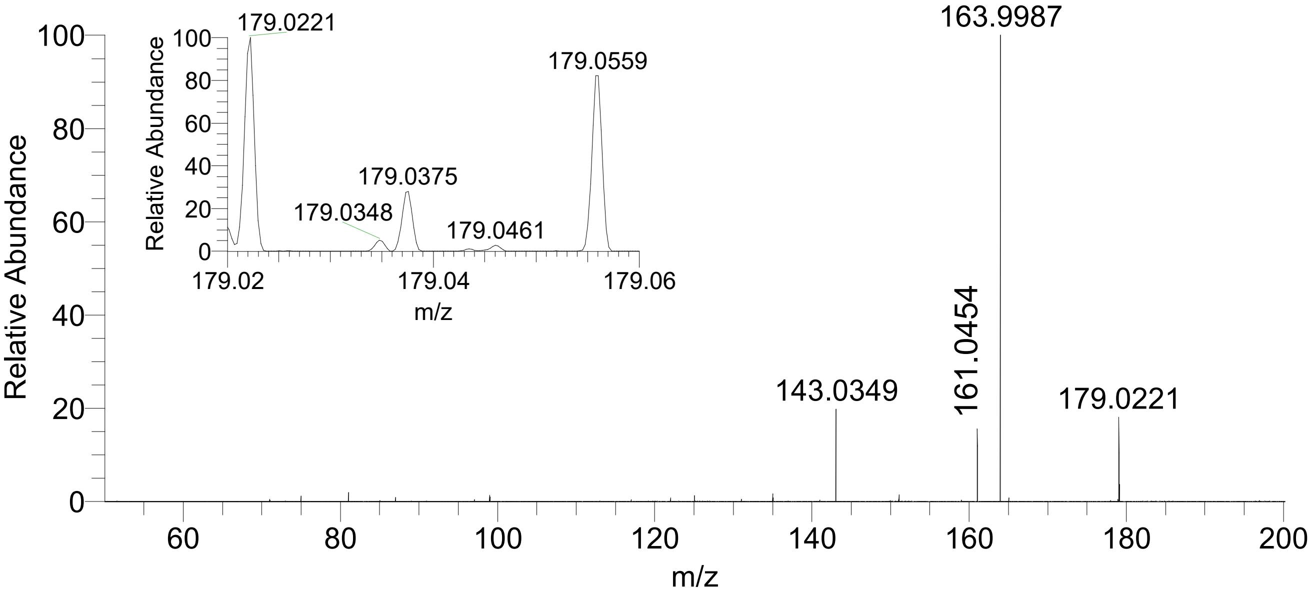

Supplementary Fig. 48 | MS/MS spectrum of a hexose (*m/z* 179.0559, [M−H]⁻) with NCE 22%. Fragments *m/z* 161.0454 and 143.0349 are consistent with predictions. The isolation window also captured *m/z* 179.0221, a fragment of HMBOA, as shown by inset.

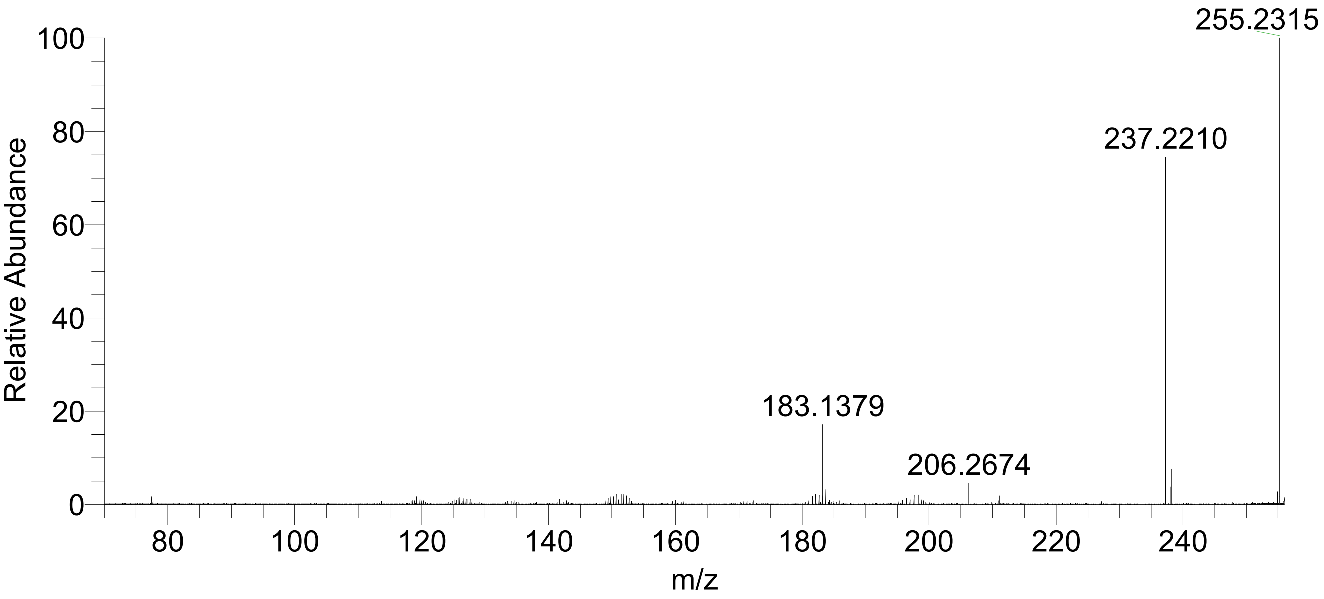

Supplementary Fig. 49 | MS/MS spectrum of FA(16:0) (*m/z* 255.2315, [M−H]⁻) with NCE 35%.

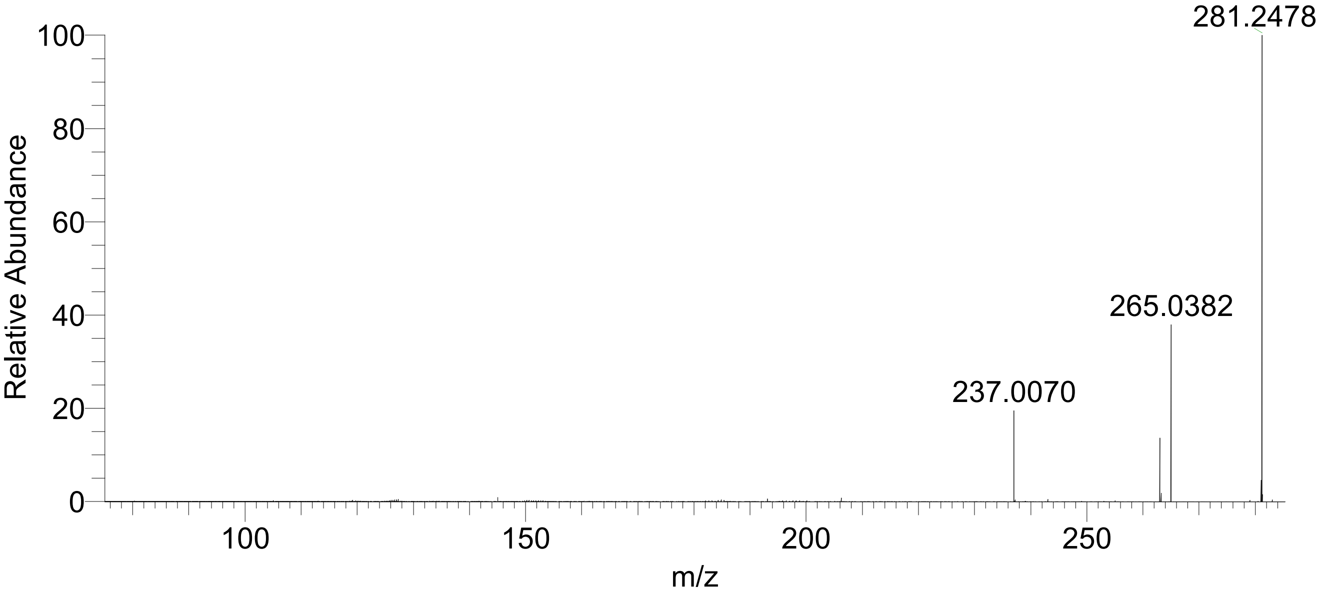

Supplementary Fig. 50 | MS/MS spectrum of a FA(18:1) (*m/z* 281.2478, [M−H]⁻) with NCE 35%.

Supplementary Table 1 | Description of replicates and ordering of imaged B73 maize roots

**Root Number** *(Biological Replicate)*	**Section Number** *(Technical Replicate)*	**Day Imaged**	**Imaging Order**	**Position in Grid of 10 Roots**[a]
3	one	1	4	1
3	two	1	3	2
3	twelve	2	10	3
3	three	1	2	4
2	four	2	8	5
2	five	2	9	6
1	seven	1	1	7
1	nine	1	5	8
1	ten	2	6	9
1	eleven	2	7	10

[a] In Supplementary Figs. 11-17 and 21-31, 1 is the top left image in the grid, and 10 is the bottom-right image in the grid. Numbering goes from left to right and top to bottom.

Supplementary Table 2 | Major fragments from MS/MS of maize B73 bulk tissue extract

Measured *m/z*	Main Fragment Ions	Tentative metabolite name	Exact *m/z*[a]	Mass error (ppm)[b]	Ion	Proposed molecular formula	Normalized CE (%)
115.0036	71.0138	fumarate	115.0037	-0.9	$[M-H]^-$	$C_4H_4O_4$	20
117.0192	99.0086<br>73.0294	succinate	117.0193	-0.9	$[M-H]^-$	$C_4H_6O_4$	20
133.0140	115.0034<br>89.0243<br>87.0086<br>72.9930<br>71.0137	malate	133.0142	-1.5	$[M-H]^-$	$C_4H_6O_5$	20
164.0349	149.0115<br>147.0450*	MBOA	164.0353	-2.4	$[M-H]^-$	$C_8H_7NO_3$	25
173.0088	155.0459<br>142.9984<br>129.0191<br>111.0086<br>85.0293<br>71.0137	aconitate	173.0092	-2.3	$[M-H]^-$	$C_6H_6O_6$	15
191.0195/<br>191.0558	173.0453<br>173.0090<br>171.0297<br>165.0043*<br>163.0609*<br>155.0349<br>154.9985<br>153.0192<br>148.9497*<br>147.0298<br>143.0349*<br>137.0243<br>129.0192<br>127.0400<br>111.0450<br>111.0086<br>109.0294<br>101.0243#<br>99.0451<br>99.0087<br>93.0345<br>87.0087#<br>85.0294#<br>81.0345<br>76.8301*<br>72.4587*	(iso)citrate/<br>quinate[c]	191.0197/<br>191.0561	-1.0/<br>-1.6	$[M-H]^-$	$C_6H_8O_7$/<br>$C_7H_{12}O_6$	25
215.0325	179.0558<br>172.9940<br>161.0454<br>149.0455<br>143.0349<br>131.0349<br>119.0349<br>113.0243<br>107.0349<br>101.0243<br>89.0243<br>87.0087<br>85.0294<br>78.9590<br>71.0138	hexose	215.0328	-1.4	$[M+Cl]^-$	$C_6H_{12}O_6$	20

Table 2 continued on next page

**Measured *m/z***	**Main Fragment Ions**	**Tentative metabolite name**	**Exact *m/z*[a]**	**Mass error (ppm)[b]**	**Ion**	**Proposed molecular formula**	**Normalized CE (%)**
279.2323	261.2218<br>235.0998	FA(18:2)	279.2330	-2.5	$[M-H]^-$	$C_{18}H_{32}O_2$	30
377.0845	341.1078<br>215.0322<br>179.0556<br>161.0452<br>143.0347<br>119.0348<br>113.0242<br>101.0242	hexose-hexose	377.0856	-2.9	$[M+Cl]^-$	$C_{12}H_{22}O_{11}$	22
745.4982	671.4621<br>507.2701<br>489.2596<br>483.2702<br>465.2597<br>415.2233<br>391.2234<br>279.2315<br>255.2316	PG(16:0/ 18:2)	745.5025	-5.8	$[M-H]^-$	$C_{40}H_{75}O_{10}P$	30

[a] Exact mass values obtained from METLIN database for relevant ion.
[b] mass error in ppm = (measured accurate mass – exact mass)/exact mass x 10^6
[c] Bolded peaks are expected in isocitrate and citrate fragmentation patterns; regular peaks are expected in the quinate fragmentation pattern; italicized peak is expected in citrate pattern; underlined peak is expected in isocitrate pattern; and # is used to indicate those peaks that are expected in quinate and citrate/isocitrate patterns, based on comparisons to database values.
* Peak does not align with expected MS/MS fragments for tentative attribution.

Supplementary Table 3 | Primers used in this study

Gene Fragment	Forward	Reverse
pHDE-SEQ	GCACATACAAATGGACGAACGG	CACAAACTTAAGCACACAACC
GFP-Xba1	CACTGATAGTTTAAACTAGTAATGGTGAGCAAG GGCGAGGA	TCACTTGTACAGCTCGTCCA
Hsp-Ter-GFP	TGGACGAGCTGTACAAGTGAATATGAAGATGAA GATGAAA	GCTAGCTTACTCAGTTAGGT CTTATCTTTAATCATATTCC
pHDE-pCYCB1;1	CTGTCAAACACTGATAGTTT CTTATTCTTTTACCTTTAAAGTTCCTCGAG	TGCTCACCATTACTAGTTT AAACTCGAGGGATCCTTCGcttagtgttctcttctctttct
pHDE-pSMR1	CTGTCAAACACTGATAGTTT acaagtgcatttttaatttgtagga	TGCTCACCATTACTAGTTT AAACTCGAGGGATCCTTCG ATCTAAACTTGTGTATgttttt
pHDE-Pme1-ACO1-CDS	CGAAGGATCCCTCGAGTTT ATGGCTTCCGAGAATCCTTTC	CCTTGCTCACCATTACTAGTTT TTGTTTGATCAAGTTCCTGATAACG
