# Supplementary material for: Chemical imaging reveals diverse functions of tricarboxylic acid metabolites in root growth and development
Source: Nat Commun. 2023 May 4;14:2567. doi: 10.1038/s41467-023-38150-z (PMC10160030; doi:10.1038/s41467-023-38150-z)
Supplement: Supplementary file 2 — Reporting Summary [file 41467_2023_38150_MOESM2_ESM.pdf]

nature portfolio

Corresponding author(s): Alexandra J. Dickinson
Richard N. Zare

Last updated by author(s): Apr 1, 2023

Reporting Summary

Nature Portfolio wishes to improve the reproducibility of the work that we publish. This form provides structure for consistency and transparency in reporting. For further information on Nature Portfolio policies, see our Editorial Policies and the Editorial Policy Checklist.

Statistics

For all statistical analyses, confirm that the following items are present in the figure legend, table legend, main text, or Methods section.

n/a	Confirmed	
☐	☒	The exact sample size (n) for each experimental group/condition, given as a discrete number and unit of measurement
☐	☒	A statement on whether measurements were taken from distinct samples or whether the same sample was measured repeatedly
☐	☒	The statistical test(s) used AND whether they are one- or two-sided<br>*Only common tests should be described solely by name; describe more complex techniques in the Methods section.*
☒	☐	A description of all covariates tested
☒	☐	A description of any assumptions or corrections, such as tests of normality and adjustment for multiple comparisons
☐	☒	A full description of the statistical parameters including central tendency (e.g. means) or other basic estimates (e.g. regression coefficient) AND variation (e.g. standard deviation) or associated estimates of uncertainty (e.g. confidence intervals)
☒	☐	For null hypothesis testing, the test statistic (e.g. F, t, r) with confidence intervals, effect sizes, degrees of freedom and P value noted<br>*Give P values as exact values whenever suitable.*
☒	☐	For Bayesian analysis, information on the choice of priors and Markov chain Monte Carlo settings
☒	☐	For hierarchical and complex designs, identification of the appropriate level for tests and full reporting of outcomes
☒	☐	Estimates of effect sizes (e.g. Cohen's d, Pearson's r), indicating how they were calculated

Our web collection on statistics for biologists contains articles on many of the points above.

Software and code

Policy information about availability of computer code

Data collection	For DESI-MSI, data were acquired using Xcalibur (version 2.5.5, Thermo Fisher) with an LTQ Orbitrap XL mass spectrometer (Thermo Fisher) coupled to a custom-built X-Y moving stage. Brightfield microscopy images were taken using a Pluggable USB 2.0 Digital Microscope with LED illumination and 250x magnification (Redmond, WA). For MS/MS, an Orbitrap Elite and an LTQ Orbitrap XL mass spectrometer (Thermo Fisher) were used to collect spectra. HPLC-MS/MS was performed on the Orbitrap Elite. Confocal images were acquired using the Leica Application Suite X software on a Leica SP8 (Leica) confocal microscope.

Data analysis

For DESI imaging, .raw files acquired in Xcalibur (version 2.5.5) were converted to .mzML files using MSConvert (64-bit GUI, a ProteoWizard tool). Each .mzML file equated to one row in the final image. The .mzML files were compiled into a single .imzML file per DESI image using imzMLConverter (version 1.3, included in the MSiReader package). All DESI images were visualized in MSiReader (version 1.03). Mass spectra of tissue rows were averaged and background was subtracted using Thermo Xcalibur Qual Browser (version 2.2, Thermo Fisher). The black background of brightfield microscopy images was removed prior to overlay on a DESI image. This was accomplished using Paint3D (Microsoft).

The resolution of the DESI-MS images was determined in Microsoft Excel 2019, using chromatograms exported from Thermo Xcalibur Qual Browser (described above). Resolution values were imported into OriginPro 2021 for further statistical analysis.

The average intensities of metabolites along the developmental axis were calculated from grayscale DESI images uploaded to FIJI (open source package, Image J).
The confocal images were further processed using Image J (version 2.3.0). Statistical analyses were performed using Prism 9 for Mac.

Additional details are available in the Methods.

For manuscripts utilizing custom algorithms or software that are central to the research but not yet described in published literature, software must be made available to editors and reviewers. We strongly encourage code deposition in a community repository (e.g. GitHub). See the Nature Portfolio guidelines for submitting code & software for further information.

Data

Policy information about availability of data

All manuscripts must include a data availability statement. This statement should provide the following information, where applicable:

- Accession codes, unique identifiers, or web links for publicly available datasets
- A description of any restrictions on data availability
- For clinical datasets or third party data, please ensure that the statement adheres to our policy

All mass spectrometry imaging files (raw and derived), associated ESI-MS/MS files, and HPLC-MS/MS files generated in this study have been deposited in the Figshare database under accession code https://doi.org/10.6084/m9.figshare.22350886. Source data are provided with this paper.

Human research participants

Policy information about studies involving human research participants and Sex and Gender in Research.

Reporting on sex and gender	Not applicable
Population characteristics	Not applicable
Recruitment	Not applicable
Ethics oversight	Not applicable

Note that full information on the approval of the study protocol must also be provided in the manuscript.

Field-specific reporting

Please select the one below that is the best fit for your research. If you are not sure, read the appropriate sections before making your selection.

☒ Life sciences ☐ Behavioural & social sciences ☐ Ecological, evolutionary & environmental sciences

For a reference copy of the document with all sections, see nature.com/documents/nr-reporting-summary-flat.pdf

Life sciences study design

All studies must disclose on these points even when the disclosure is negative.

Sample size	Sample sizes were chosen to make sure that the observed effects are significant by statistical analysis. Analyses were performed on at least three biological replicates.
Data exclusions	No data were excluded from the analysis.
Replication	The number of independent biological replicates is stated for each experiment.
Randomization	The transgenic plants of different constructs were randomly selected with no formal randomization techniques to minimize the position effect on the genome.
Blinding	Not applicable.

Reporting for specific materials, systems and methods

We require information from authors about some types of materials, experimental systems and methods used in many studies. Here, indicate whether each material, system or method listed is relevant to your study. If you are not sure if a list item applies to your research, read the appropriate section before selecting a response.

Materials & experimental systems

n/a	Involved in the study
☒	☐ Antibodies
☒	☐ Eukaryotic cell lines
☒	☐ Palaeontology and archaeology
☒	☐ Animals and other organisms
☒	☐ Clinical data
☒	☐ Dual use research of concern

Methods

n/a	Involved in the study
☒	☐ ChIP-seq
☒	☐ Flow cytometry
☒	☐ MRI-based neuroimaging
